# A sustainable strategy for the straightforward preparation of 2*H*-azirines and highly functionalized *NH*-aziridines from vinyl azides using a single solvent flow-batch approach

**DOI:** 10.3762/bjoc.17.20

**Published:** 2021-01-20

**Authors:** Michael Andresini, Leonardo Degannaro, Renzo Luisi

**Affiliations:** 1 Flow Chemistry and Microreactor Technology FLAME-Lab, Department of Pharmacy – Drug Sciences, University of Bari “A. Moro”, Via E. Orabona 4, Bari, 70125, Italy

**Keywords:** aziridines, 2*H*-azirines, flow chemistry, green chemistry, organolithium compounds

## Abstract

The reported flow-batch approach enables the easy preparation of 2*H*-azirines and their stereoselective transformation into highly functionalized *NH*-aziridines, starting from vinyl azides and organolithium compounds. The protocol has been developed using cyclopentyl methyl ether (CPME) as an environmentally benign solvent, resulting into a sustainable, safe and potentially automatable method for the synthesis of interesting strained compounds.

## Introduction

Since their conception in the early 1990s, Green Chemistry Principles (GCP) have been applied with increasing effort towards the design of efficient production processes [1–3]. As a result, a number of sustainable synthetic strategies has been recently developed, lowering the environmental impact and reducing the chemical hazards associated with the preparation of highly valuable compounds [4]. Among the elements that affect the sustainability of a synthetic method, the choice of the solvent is crucial [5]. In fact, chemical solvents represent most of the total amount of chemical species used in manufacturing processes, and therefore, strongly affect waste disposal requirements and process related risks [6]. Recently, a variety of sustainable solvents has been therefore identified, and their use have been combined with those of enabling technologies. In this scenario, the development of continuous flow synthetic methodologies has found its fortune in the past two decades [7–9]. Several chemical hazards can be effectively controlled through the use of microfluidic systems, because of the utilization of a reduced confined space and the exquisite control over heating and mass transfer [10]. In fact, the utilization of the microfluidic technology results in smaller temperature gradients due to the large surface–volume ratio of microreactors, and may prevent the formation of undesirable byproducts that are not avoidable under the traditional batch conditions. As a consequence, efforts have been devoted to the development of synthetic processes combining GCP and enabling technologies [11].

Within the synthetic chemistry context, the preparation of aziridines still generates interest, mostly because of their importance as source for drug prototypes and drug discovery leads [12]. Interestingly, important advances have been recently addressed in the synthesis of *NH*-aziridines directly from olefins [13–14]. Aziridines are otherwise accessible from a variety of acyclic precursors [15–17], even stereoselectively [18–20], and through derivatization of 2*H*-azirines. The reactions using 2*H*-azirines as electrophiles proceed with several nitrogen, oxygen and sulfur nucleophiles, enabling to access aziridines with great structural variability [21]. The reaction of azirines with Grignard and organolithium reagents has been poorly investigated, and only without using green and renewable solvents [22–23]. In turn, 2*H*-azirines can be smoothly obtained through intramolecular cyclization of vinyl azides, or by other strategies involving oximes, imines and oxazoles [24].

**Scheme 1:**
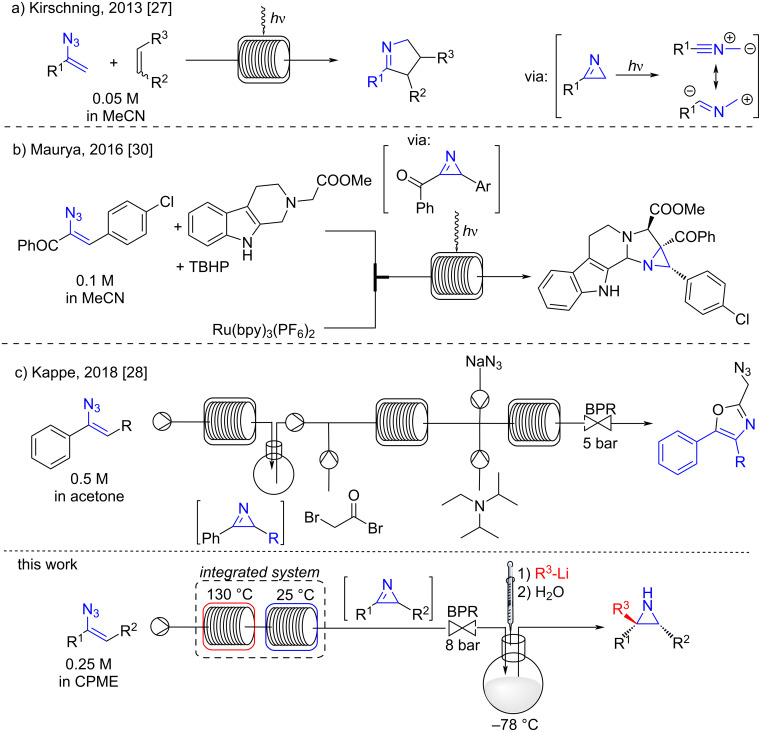
Flow generation and transformation of 2*H*-azirines.

One appealing strategy for the preparation of 2*H*-azirines involves the use of readily available vinyl azides [25–30]. However, the batch cyclization of vinyl azides into the corresponding 2*H*-azirines could generate some risks, due to the explosive nature of organic azides, and possible overpressure issues caused by nitrogen generation at high temperatures. Consequently, scalability and control of this processes represents a real challenge. The exploitation of microfluidic technologies has therefore resulted in safer procedures for the preparation of 2*H*-azirines, offering valuable alternatives for production purposes. In 2013, Kirschning harnessed the photoinduced electrocyclization of vinyl azides in a microfluidic photoreactor yielding 2*H*-azirines as precursors of 1,3-dipolarophiles (Scheme 1a) [27]. Similarly, Maurya developed a microfluidic photoreactor for the synthesis of a fused β-carboline from an α-ketovinyl azide and a 1,2,3,4-tetrahydro-β-carboline (Scheme 1b) [30]. More recently, Kappe reported the generation of 2*H*-azirines under continuous flow conditions, and their transformation into functionalized oxazoles using acetone as the solvent (Scheme 1c) [28]. Inspired by these recent reports, we became interested in the development of an eco-friendly strategy for the safe preparation of highly functionalized *NH*-aziridines from acyclic precursors. Herein, we report a sustainable mixed flow-batch approach that enables the direct preparation of functionalized *NH*-aziridines from vinyl azides.

## Results and Discussion

At the earliest stage of our research, we focused on the choice of the most suitable solvent for azide cyclization and organolithium addition reactions. Most of the previously reported flow procedures involved acetonitrile, dichloromethane and acetone as solvents, however, incompatible with the utilization of reactive alkali organometals. An exception is made for toluene, used by Kirschning for the photoinduced azirine formation [27]. Therefore, we investigated the thermally induced cyclisation of 1-(1-azidovinyl)-4-methylbenzene (**1a**) in refluxing 2-MeTHF and cyclopentyl methyl ether (CPME) as green solvent candidates, and compared the results with the reaction conducted in toluene (Table 1).

**Table 1:** Thermally induced cyclization of 1-(1-azidovinyl)-4-methylbenzene (**1a**) under batch conditions.

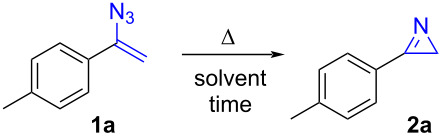		

Solvent	Temperature	Time^a^

toluene	110 °C	1.5 h
2-MeTHF	80 °C	4.0 h
CPME	106 °C	45 min

^a^Time needed for complete consumption of **1a**.

The reaction proceeded rapidly in CPME, while the use of 2-MeTHF resulted in longer reaction times if compared with toluene. We therefore selected CPME as the most suitable solvent for our purposes. Interestingly, besides being characterized by low toxicity, CPME has a very low affinity to water, making it suitable for moisture sensitive reactions, without previous distillation [31–33]. Subsequently, the process was examined under continuous flow conditions employing a special integrated coil reactor with two different operating temperatures (see Supporting Information File 1). A solution of 1-(1-azidovinyl)-4-methylbenzene (**1a**, 0.25 M in CPME) was introduced, via a high pressure syringe pump, into the coil reactor maintained at the temperature of 130 °C, and the residence time varied by adjusting the flow rate (Table 2). The reaction yield was calculated by ^1^H NMR analysis of the crude. In details, conversion of **1a** in **2a** increased from 33% to >99% by adjusting the residence time from 4 min to 16 min, respectively. The complete transformation of vinyl azide **1a** was therefore achieved above the boiling point of CPME (106 °C), as enabled by the utilization of a microfluidic reactor. From a technical point of view, the pressure generated during the course of the reaction, due to nitrogen evolution, could be managed by using a high pressure pump, a stainless-steel reactor and a back-pressure regulator at 8 bar.

**Table 2:** Flow cyclization of 1-(1-azidovinyl)-4-methylbenzene (**1a**).

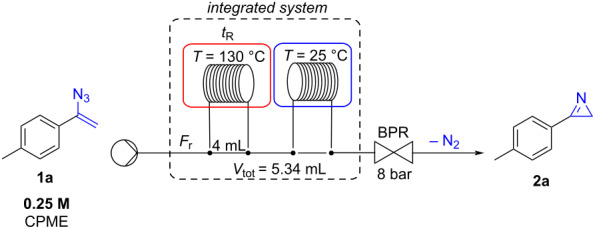			

Entry	Flow rate (*F*_r_)	Residence time (*t*_R_)	Yield of **2a**

1	1.00 mL/min	4 min	33%
2	0.50 mL/min	8 min	65%
3	0.25 mL/min	16 min	>99%

**Scheme 2:**
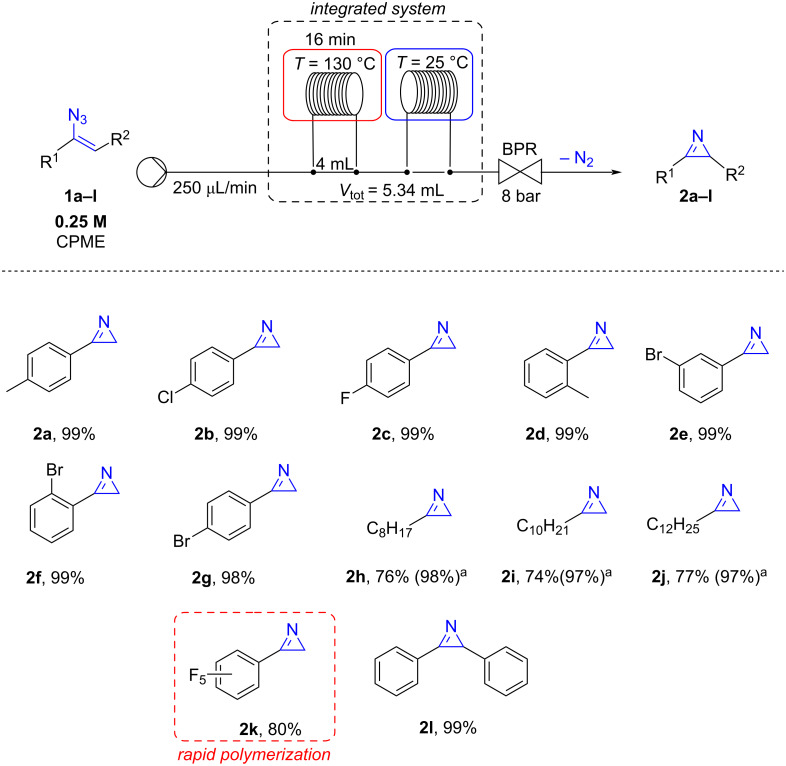
Flow synthesis of 2*H*-azirines from vinyl azides. ^a^The solution of vinyl azide was re-introduced twice into the flow system to achieve full conversion.

Under the optimized conditions, the scope of the reaction was explored on vinyl azides **1a**–**l** that were transformed into the corresponding 2*H*-azirines **2a**–**l** (Scheme 2). The methodology was found to be efficient with vinyl azides carrying aryls substituted with chlorine (**2b**), fluorine (**2c**), and bromide (**2e**–**g**). In details, *ortho*-, *meta*-, and *para*-bromophenyl derivatives were quantitatively transformed into the corresponding 2*H*-azirines **2e–g** without substantial differences. Similarly, 3-(*o*-tolyl)-2*H*-azirine (**2d**) and 2,3-diphenyl-2*H*-azirine (**2l**) were also obtained in excellent yields. When 1-(1-azidovinyl)-2,3,4,5,6-pentafluorobenzene (**1k**) was reacted under optimal flow conditions, a mixture of **1k** and 2*H*-azirine **2k** (20:80 ratio) was recovered. Unfortunately, it was not possible to isolate 3-(perfluorophenyl)-2*H*-azirine (**2k**) due to its rapid polymerization in the crude mixture.

Interestingly, aliphatic vinyl azides **1h–j** were found less reactive, and the corresponding azirines **2h–j** were obtained in just 74–77% yield, in mixtures with unreacted starting materials. However, when the reaction crudes were re-introduced into the flow reactor under the same conditions, azirines **2h–j** were obtained quantitatively (**2h**, 98%; **2i**, 97%; **2j**, 97%).

**Scheme 3:**
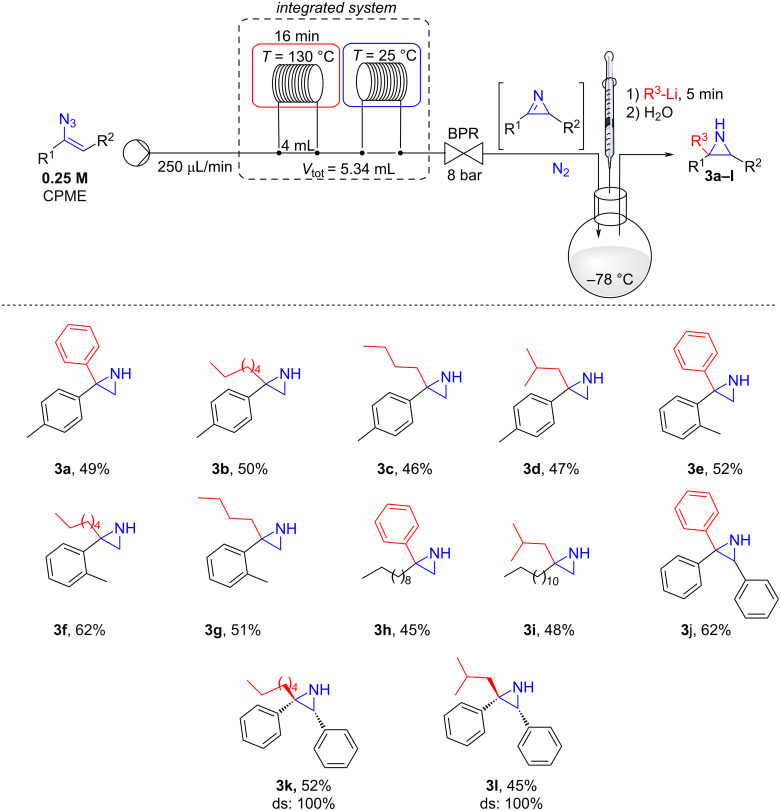
Mixed flow-batch approach for the preparation of functionalized *NH*-aziridines from vinyl azides.

Pursuing in our aim to develop a green approach to prepare substituted *NH*-aziridines from vinyl azides in a single procedure, the solution of **2a** from the microfluidic system was collected in a round bottom flask cooled at −78 °C, and reacted with 1.2 equivalents of phenyllithium (PhLi) [34–35]. The mixture was stirred at the same temperature for 5 minutes, before quenching with water. To our delight, product **3a** was isolated in 49% yield. Next, the reactions of several commercially available organolithium compounds were examined. As shown in Scheme 3, the reaction of **2a**, generated in flow from **1a**, proceeded smoothly also with hexyllithium (HexLi), *n*-butyllithium (*n-*BuLi) and isobutyllithium (iBuLi) affording the corresponding *NH*-aziridines **3b–d** in good yields. Subsequently, other vinyl azides were tested for this flow-batch two-step procedure (Scheme 3).

Starting from vinyl azide **1d**, the corresponding 3-(*o*-tolyl)-2*H*-azirine (**2d**) was generated under flow conditions, and reacted with PhLi, HexLi and BuLi, furnishing *NH*-azirdines **3e**–**g**. Aliphatic vinyl azides **1i** and **1j** were also subjected to this mixed flow-batch protocol, generating *NH*-aziridines **3h**,**i** in good yields (Scheme 3). The reaction was found to be efficient when PhLi was added to the collected solution of 2,3-diphenyl-2*H*-azirine (**2m**), affording 2,2,3-triphenylaziridine (**3j**) in 62% yield. Moreover, the reaction was found fully diastereoselective when HexLi or iBuLi were added to **2m**. In fact, only products deriving from the attack of the organolithium on the less hindered face (i.e., *anti* with respect to the phenyl substituent at C3), leading to **3k** (52%), and **3l** (45%), were observed. The relative stereochemistry was assigned by NOESY experiments (see Supporting Information File 1). Unfortunately, when the protocol was applied to 1-(1-azidovinyl)-2,3,4,5,6-pentafluorobenzene (**1k**), only a complex mixture was recovered likely because of the instability of the corresponding 2*H*-azirine **2k** (vide infra).

## Conclusion

In summary, we have developed a sustainable mixed flow-batch approach for the synthesis of functionalized *NH*-aziridines starting from vinyl azides using a single green solvent for two reaction steps. Several vinyl azides have been quantitatively transformed into the corresponding 2*H*-azirines in a microfluidic reactor, overcoming the hazards associated with this transformation under batch conditions. A small library of functionalized aryl and alkyl-substituted *NH*-aziridines has been created under operationally simple conditions. Notably, the addition reaction was found to proceed stereoselectively when organolithium compounds were added to 2,3-diphenyl-2*H*-azirine. This is a fast, safe, green and convenient method to access this interesting structural motif without requiring protection/deprotection steps or long synthetic pathways.

## Supporting Information

File 1 Description of general methods, general procedures, characterization data for all compounds and copies of ^1^H,^13^C,^19^F, NOESY spectra.
