# Supplementary material for: A sustainable strategy for the straightforward preparation of 2H-azirines and highly functionalized NH-aziridines from vinyl azides using a single solvent flow-batch approach
Source: Beilstein J Org Chem. 2021 Jan 20;17:203–9. doi: 10.3762/bjoc.17.20 (PMC7849244; doi:10.3762/bjoc.17.20)
Supplement: File 1 — Description of general methods, general procedures, characterization data for all compounds and copies of 1H,13C,19F, NOESY spectra. [file Beilstein_J_Org_Chem-17-203-s001.pdf]

BEILSTEIN JOURNAL OF ORGANIC CHEMISTRY

# Supporting Information

for

# A sustainable strategy for the straightforward preparation of 2*H*-azirines and highly functionalized *NH*-aziridines from vinyl azides using a single solvent flow-batch approach

Michael Andresini, Leonardo Degannaro and Renzo Luisi

*Beilstein J. Org. Chem.* **2021,** *17,* 203–209. doi:10.3762/bjoc.17.20

## Description of general methods, general procedures, characterization data for all compounds and copies of $^{1}H$,$^{13}C$,$^{19}F$, NOESY spectra

License and Terms: This is a supporting information file under the terms of the Creative Commons Attribution License (https://creativecommons.org/licenses/by/4.0). Please note that the reuse, redistribution and reproduction in particular requires that the author(s) and source are credited and that individual graphics may be subject to special legal provisions.
The license is subject to the *Beilstein Journal of Organic Chemistry* terms and conditions: (https://www.beilstein-journals.org/bjoc/terms)

# Table of Contents


**1.** General methods **S2**

**2.** General Procedure A for the preparation of vinyl azides **S3**

Characterization data for compounds **1a–l** **S3**

**3.** General Procedure B for the preparation of 2*H*-azirines **S7**

Characterization data for compounds **2a–l** **S7**

**4.** General Procedure C for the preparation of *NH*-aziridines **S11**

**5.** Characterization data for compounds **3a–l** **S12**

**6.** Copies of $^{1}H$, $^{13}C$, $^{19}F$, NOESY NMR spectra **S17**

**7.** References **S47**

## 1. General methods

All reagents were purchased from Sigma-Aldrich, TCI, Alfa Aesar and Fluorochem, and used without previous purification. Tin-layer chromatography (TLC) was performed on a 0.25 mm precoated silica gel thick plates 60F254 (Merck); the spots were visualized under UV light (λ = 254 nm) and/or $KMnO_{4\ (aq)}$. Flash chromatography was performed using 230–400 mesh silica and a mobile phase as indicated for each entry, according to standard techniques. HRMS spectra were recorded on Agilent 6530 accurate mass Q-TOF instrument using electrospray ion source (ESI), operating in positive and negative ion mode, as described for each entry. Infrared spectra ($ν_{max}$, FT-IR/ATR) were recorded in reciprocal centimeters ($cm^{-1}$) using a PerkinElmer 283 Spectrometer (FT-IR) or a PerkinElmer Spectrum Two Spectrometer with a 2 × 2 mm diamond crystal (ATR). Nuclear magnetic resonance spectra were recorded using an Agilent 500 spectrometer (500 MHz for $^1$H, 125 MHz for $^{13}$C, 470 MHz for $^{19}$F), and a Varian Mercury 300 spectrometer (300 MHz for $^1$H, 75 MHz for $^{13}$C, 282 MHz for $^{19}$F). The peak of the (residual) solvent signal was used as an internal standard, related to TMS, with δ 7.26 ppm ($^1$H in $CDCl_3$), δ 77.00 ppm ($^{13}$C in $CDCl_3$). For $^{19}$F spectra, absolute referencing was used. Spin-spin coupling constants (*J*) are given in Hz. Assignment of the resonances was performed by combined application of standard NMR techniques (HSQC, COSY). Assignment of relative stereochemistry for compounds **3k** and **3l** was performed by NOESY experiments.

**Flow equipment**: Solutions of the reagents were introduced into the flow microreactor system using a Harvard PHD 2000 syringe pump, equipped with gastight syringe purchased from SGE (Harvard PHD 2000). A Volcano reactor (Syrris, stainless steel tubular reactor, 4mL) was employed. Connections were obtained using stainless steel and PTFE microtubes with an inner diameter of 500μL. Microtubes were connected to the reactor by with stainless steel fittings (GL Sciences, 1/16 OUW).

## 2. General Procedure A for the preparation of vinyl azides

Vinil azides **1a–l** were prepared according to the reported procedures with slight modification, starting from alkenes.[1,2]

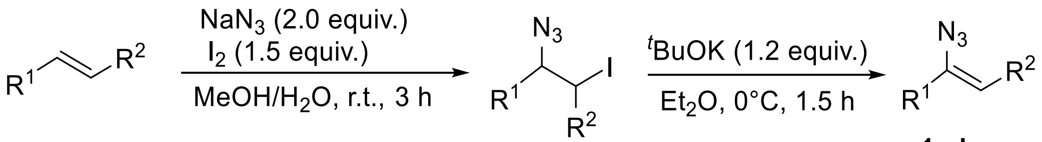


**1a-l**

To a solution of alkene (8.5 mmol, 1.0 equiv) in 18 mL of solvent ($MeOH/H_2O$ = 5:1), sodium azide (17.0 mmol, 2.0 equiv) was added in one portion at 25 °C, then iodine (12.8 mmol, 1.5 equiv) was added, and the solution was stirred for 3 h. Subsequently, $CH_2Cl_2$ (90 mL) and $H_2O$ (50 mL) were added, the organic layer separated and washed with an aqueous solution of $Na_2S_2O_5$ (5%) until the organic phase appeared colourless. The organic layer was washed twice with $H_2O$ (2 × 35 mL), dried over $Na_2SO_4$, the solvent was evaporated under reduced pressure and the product was immediately used for the next synthetic step without further purification. To a solution of the obtained beta-iodo azide in $Et_2O$ (17 mL), *t*-BuOK (10.2 mmol, 1.2 equiv) was added at 0°C and the reaction mixture was stirred at the same temperature for 1.5 h. Subsequently, the mixture was filtered through a pad of diatomaceous earth, and the solvent evaporated under reduced pressure. Purification by column chromatography afforded vinyl azides **1a–l** as reported for each entry.

**Characterization data for vinyl azides 1a–l**

**1-(1-Azidovinyl)-4-methylbenzene (1a)**

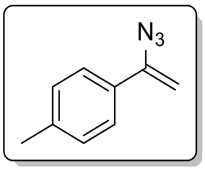


Prepared according General Procedure A. The product was purified by column chromatography ($SiO_2$, $R_f$ 0.9, Hexane) to afford vinyl azide **1a** as a pale yellow oil (812 mg, 60%). $^1$H NMR (300 MHz, $CDCl_3$, ppm) δ 7.51 (d, $J$ = 8.0 Hz, 2H, Ar–H), 7.21 (d, $J$ = 8.0 Hz, 2H, Ar–H), 5.43 (d, $J$ = 2.2 Hz, 1H), 4.96 (d, $J$ = 2.2 Hz, 1H), 2.41 (s, 3H, $CH_3$). Analytical data (NMR) in agreement with those reported in the literature.[3]

**1-(1-Azidovinyl)-4-chlorobenzene (1b)**

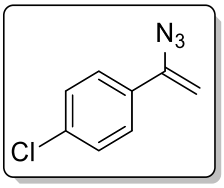


Prepared according General Procedure A. The product was purified by column chromatography ($SiO_2$, $R_f$ 0.9, Hexane) to afford vinyl azide **1b** as a pale yellow oil (1068

mg, 70%). $^1H$ NMR (500 MHz, $CDCl_3$, ppm) δ 7.50–7.48 (m, 2H, Ar–H), 7.34–7.31 (m, 2H, Ar–H), 5.43 (d, *J* = 2.6 Hz, 1H), 4.97 (d, *J* = 2.6 Hz, 1H). Analytical data (NMR) in agreement with those reported in the literature.[3]

**1-(1-Azidovinyl)-4-fluorobenzene (1c)**

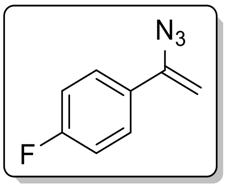


Prepared according General Procedure A. The product was purified by column chromatography ($SiO_2$, $R_f$ 0.9, Hexane) to afford vinyl azide **1c** as a pale yellow oil (651 mg, 47%). $^1H$ NMR (500 MHz, $CDCl_3$, ppm) δ 7.57–7.50 (m, 2H, Ar–H), 7.08–7.00 (m, 2H, Ar–H), 5.37 (d, *J* = 2.6 Hz, 1H), 4.94 (d, *J* = 2.6 Hz, 1H). $^{19}F$ NMR (282 MHz, $CDCl_3$, ppm) δ -112.31 (ddd, *J* = 13.6, 8.5, 5.3 Hz). Analytical data (NMR) in agreement with those reported in the literature.[3]

**1-(1-Azidovinyl)-2-methylbenzene (1d)**

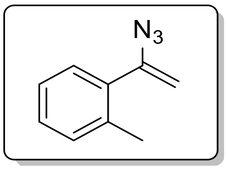


Prepared according General Procedure A. The product was purified by column chromatography ($SiO_2$, $R_f$ 0.9, Hexane) to afford vinyl azide **1f** as a pale yellow oil (730 mg, 54%). $^1H$ NMR (300 MHz, $CDCl_3$, ppm) δ 7.28–7.18 (m, 4H, Ar–H), 5.05 (s, 1H), 4.74 (s, 1H), 2.39 (s, 3H, $CH_3$). Analytical data (NMR) in agreement with those reported in the literature.[3]

**1-(1-Azidovinyl)-3-bromobenzene (1e)**

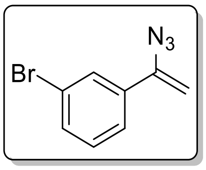


Prepared according General Procedure A. The product was purified by column chromatography ($SiO_2$, $R_f$ 0.9, Hexane) to afford vinyl azide **1d** as a brown oil (952 mg, 50%). $^1H$ NMR (300 MHz, $CDCl_3$, ppm) δ 7.72–7.70 (m, 1H, Ar–H), 7.50–7.45 (m, 2H, Ar–H), 7.22 (t, *J* = 7.9 Hz, 1H, Ar–H), 5.45 (d, *J* = 2.7 Hz, 1H), 4.98 (d, *J* = 2.7 Hz, 1H). Analytical data (NMR) in agreement with those reported in the literature.[4]

**1-(1-Azidovinyl)-2-bromobenzene (1f)**

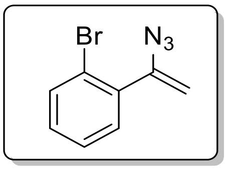


Prepared according General Procedure A. The product was purified by column chromatography ($SiO_2$, $R_f$ 0.9, Hexane) to afford vinyl azide **1f** as a brown oil (1257 mg, 66%). $^1$H NMR (300 MHz, $CDCl_3$, ppm) δ 7.62 (d, $J$ = 7.7 Hz, 1H, Ar–H), 7.36–7.32 (m, 2H, Ar–H), 7.26–7.23 (m, 1H, Ar–H), 5.12 (d, $J$ = 1.2 Hz, 1H), 4.85 (d, $J$ = 1.2 Hz, 1H). Analytical data (NMR) in agreement with those reported in the literature.[3]

**1-(1-Azidovinyl)-4-bromobenzene (1g)**

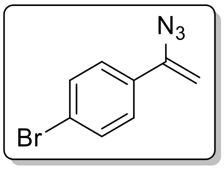


Prepared according General Procedure A. The product was purified by column chromatography ($SiO_2$, $R_f$ 0.9, Hexane) to afford vinyl azide **1g** as a brown oil (1714 mg, 90%). $^1$H NMR (300 MHz, $CDCl_3$, ppm) δ 7.49–7.47 (m, 2H, Ar–H), 7.44–7.42 (m, 2H, Ar–H), 5.44 (d, $J$ = 2.4 Hz, 1H), 4.98 (d, $J$ = 2.4 Hz, 1H). Analytical data (NMR) in agreement with those reported in the literature.[3]

**2-Azidodec-1-ene (1h)**

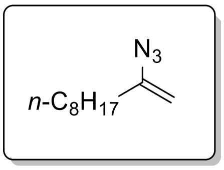


Prepared according General Procedure A. The product was purified by column chromatography ($SiO_2$, $R_f$ 0.95, Hexane) to afford azide **1i** as a colourless oil (478 mg, 31%).

$^1$H NMR (300 MHz, $CDCl_3$, ppm) δ 4.62 (broad signal, 2H, C=$CH_2$), 2.07 (t, $J$ = 7.5 Hz, 2H), 1.51–1.42 (m, 2H), 1.37–1.26 (m, 10H), 0.88 (t, $J$ = 6.8 Hz, 3H). Analytical data (NMR) in agreement with those reported in the literature.[5]

**2-Azidododec-1-ene (1i)**

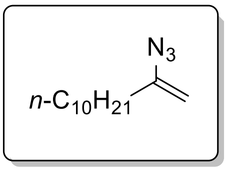


Prepared according General Procedure A. The product was purified by column chromatography ($SiO_2$, $R_f$ 0.95, Hexane) to afford azide **1j** as a colourless oil (961 mg, 54%). $^1$H NMR (300 MHz, $CDCl_3$, ppm) δ 4.62 (broad signal, 2H, C=$CH_2$), 2.06 (t, $J$ = 7.5 Hz, 2H), 1.49–1.44 (m, 2H), 1.38–1.23 (m, 14H), 0.88 (t, $J$ = 6.8 Hz, 3H). Analytical data (NMR) in agreement with those reported in the literature.[6]

**2-Azidotetradec-1-ene (1j)**

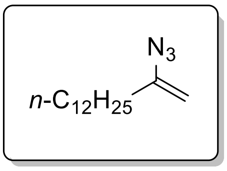


Prepared according General Procedure A. The product was purified by column chromatography ($SiO_2$, $R_f$ 0.95, Hexane) to afford azide **1k** as a colourless oil (1190 mg, 59%). $^1H$ NMR (300 MHz, $CDCl_3$, ppm) δ 4.62 (d, $J$ = 0.8 Hz, 2H, C=$CH_2$), 2.06 (t, $J$ = 7.5 Hz, 2H), 1.49–1.43 (m, 2H), 1.36–1.24 (broad signal, 18H), 0.88 (t, $J$ = 6.6 Hz, 3H). $^{13}C$ NMR (75 MHz, $CDCl_3$, ppm) δ 147.0 ($N_3C$=$CH_2$), 98.1 ($N_3C$=$CH_2$), 33.8 ($CH_2$), 32.1 ($CH_2$), 29.8 (3 x $CH_2$), 29.7 ($CH_2$), 29.5 (2 x $CH_2$), 29.0 ($CH_2$), 27,5 ($CH_2$), 22.8 ($CH_2$), 14.2 ($CH_3$). IR (ATR, neat)/$cm^{-1}$ 2955, 2853, 2104, 1626, 1466, 1274. HRMS (ESITOF) m/z $(M–H)^-$ calcd for $C_{14}H_{27}N_3$ 236,2132; found 236,2125.

**1-(1-Azidovinyl)-2,3,4,5,6-pentafluorobenzene (1k)**

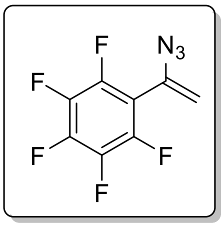


Prepared according General Procedure A. The product was purified by column chromatography ($SiO_2$, $R_f$ 0.9, Hexane) to afford vinyl azide **1l** as a pale yellow oil (700 mg, 35%). $^1H$ NMR (300 MHz, $CDCl_3$, ppm) δ 5.40 (d, $J$ = 2.3 Hz, 1H), 5.12 (d, $J$ = 2.3 Hz, 1H). $^{19}F$ NMR (282 MHz, $CDCl_3$, ppm) δ -138.69 – -141.44 (m), -152.16 (tt, $J$ = 21.0, 2.4 Hz), –158.59 – -162.86 (m). $^{13}C$ NMR (125 MHz, $CDCl_3$, ppm) δ 144.6 (m, C–F), 141.8 (m, C–F), 137.8 (m, C–F), 132.0 ($N_3C$=$CH_2$), 110.5 (m, Ar–$C_q$), 108.3 ($N_3C$=$CH_2$). IR (ATR, neat)/$cm^{-1}$ 2151, 2102, 1493, 1327, 989, 707. HRMS (ESITOF) m/z $(M–H)^-$ calcd for $C_8HF_5N_3$ 234,0096; found 234,0110.

**(*Z*)-(1-Azidoethene-1,2-diyl)dibenzene (1l)**

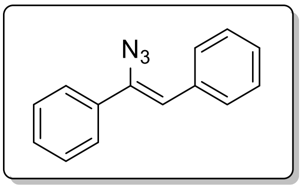


Prepared according General Procedure A. The product was purified by column chromatography ($SiO_2$, $R_f$ 0.9, Hexane) to afford azide **1m** as a colourless oil (564 mg, 30%). $^1H$ NMR (300 MHz, $CDCl_3$, ppm) δ 7.72 (d, $J$ = 7.8 Hz, 2H), 7.54–7.42 (m, 7H), 7.28–7.26 (m, 1H), 6.03 (s, 1H). Analytical data (NMR) in agreement with those reported in the literature.[6]

## 3. General Procedure B

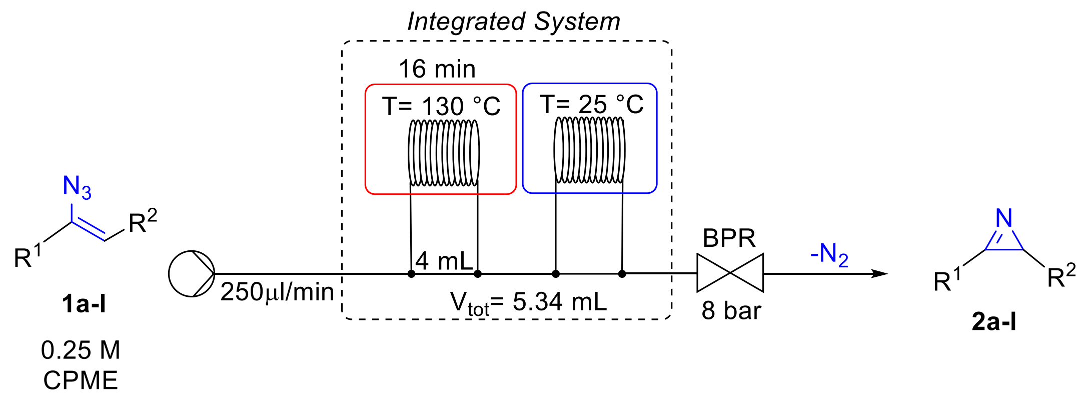


The process can be executed using a PHD ULTRA™ Syringe Pump (Harvard Apparatus), a Volcano reactor (4 mL, Syrris) and a back–pressure regulator of 8 bar. A solution of vinyl azide (1.0 mmol) in CPME (4 mL, 0.25 M) was introduced by syringe pump into the pre–heated reactor (130 °C, probe feedback control) with a flow rate of 250 µL/min. Subsequently, fresh solvent (CPME) was fluxed in the reactor upon the same conditions, and the outgoing solution was collected in a round bottom flask. The solvent was evaporated under reduced pressure and the products were obtained after chromatography or without any further purification as indicated for each entry.

### Characterization data for azirines 2a–l

### 3-(*p*-Tolyl)-2*H*-azirine (2a)

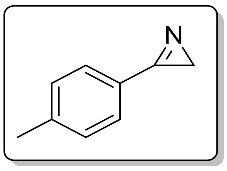


Prepared according General Procedure B using vinyl azide **1a** (159 mg). The product was obtained without any further purification as a yellow oil (131 mg, 99%). $^{1}$H NMR (300 MHz, $CDCl_3$, ppm) δ 7.80 (d, *J* = 8.0 Hz, 2H, Ar–H), 7.36 (d, *J* = 8.0 Hz, 2H, Ar–H), 2.46 (s, 3H, Ar–$CH_3$), 1.76 (s, 2H, $NCH_2$). Analytical data (NMR) in agreement with those reported in the literature.[7]

### 3-(4-Chlorophenyl)-2*H*-azirine (2b)

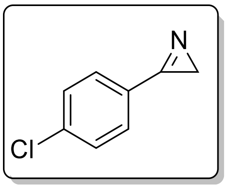


Prepared according General Procedure B using vinyl azide **1b** (180 mg). The product was obtained without further purification as a yellow oil (150 mg, 99%). $^{1}$H NMR (300 MHz, $CDCl_3$, ppm) δ 7.86–7.83 (m, 2H, Ar–H), 7.56–7.53 (m, 2H, Ar–H), 1.75 (s, 2H, $NCH_2$). Analytical data (NMR) in agreement with those reported in the literature.[7]

**3-(4-Fluorophenyl)-2*H*-azirine (2c)**

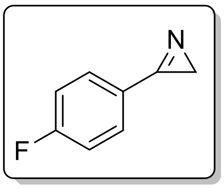

Prepared according General Procedure B using vinyl azide **1c** (163 mg). The product was obtained without further purification as a yellow oil (134 mg, 99%). $^1$H NMR (300 MHz, $CDCl_3$, ppm) δ 7.94–7.90 (m, 2H, Ar–H), 7.28–7.24 (m overlapping $CDCl_3$, 2H, Ar–H), 1.80 (s, 2H, $NCH_2$). $^{19}$F NMR (470 MHz, $CDCl_3$, ppm) δ -104.79 (m, 1F) Analytical data (NMR) in agreement with those reported in the literature.[7]

**3-(*o*-Tolyl)-2*H*-azirine (2d)**

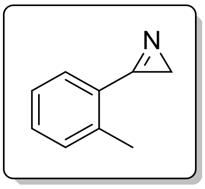

Prepared according General Procedure B using vinyl azide **1d** (159 mg). The product was obtained without further purification as a yellow oil (131 mg, 99%). $^1$H NMR (500 MHz, $CDCl_3$, ppm) δ 7.75 (d, *J* = 7.5 Hz, 1H, Ar–H), 7.47 (t, *J* = 7.5 Hz, 1H, Ar–H), 7.39 (t, *J* = 7.5 Hz, 1H, Ar–H), 7.35 (d, *J* = 7.6 Hz, 1H, Ar–H) 2.70 (s, 3H, Ar–$CH_3$), 1.69 (s, 2H, $NCH_2$). $^{13}$C NMR (125 MHz, $CDCl_3$, ppm) δ 165.1 (C=N), 140.9 (Ar), 132.5 (Ar), 132.3 (Ar), 130.9 (Ar), 126.3 (Ar), 124.0 (Ar), 19.9 ($NCH_2$), 17.9 (Ar–$CH_3$). IR (ATR, neat)/cm$^{-1}$ 3042, 2976, 2924, 1734, 1488, 981, 759, 669. HRMS (ESITOF) m/z $(M+H)^+$ calcd for $C_9H_{10}N$ 132,0813; found 132,0807.

**3-(3-Bromophenyl)-2*H*-azirine (2e)**

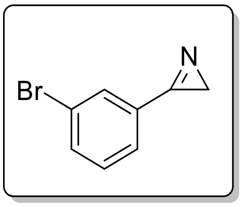

Prepared according General Procedure B using vinyl azide **1e** (224 mg). The product was obtained without further purification as a brown oil (195 mg, 99%). $^1$H NMR (300 MHz, $CDCl_3$, ppm) δ 8.05 (t, *J* = 1.7 Hz, 1H, Ar–H), 7.90–7.81 (m, 1H, Ar–H), 7.72 (ddd, *J* = 8.0, 1.7, 1.0 Hz, 1H, Ar–H), 7.44 (t, *J* = 8.0 Hz, 1H, Ar–H), 1.82 (s, 2H, C=$NCH_2$). $^{13}$C NMR (125 0MHz, $CDCl_3$, ppm) δ 165.5 (C=N), 135.9 (Ar), 132.5 (Ar), 130.77 (Ar), 128.1 (Ar), 127.6 (Ar), 123.2 (Ar), 20.4 ($NCH_2$). IR (film)/cm$^{-1}$ 3052, 2978, 2101, 1742, 1566, 1291, 993, 787. HRMS (ESITOF) m/z $(M+H)^+$ calcd for $C_8H_7BrN$ 195,9762; found 195,9753.

**3-(2-Bromophenyl)-2*H*-azirine (2f)**

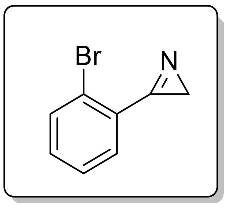

Prepared according General Procedure B using vinyl azide **1f** (224 mg). The product was obtained without further purification as a brown oil (195 mg, 99%). $^1$H NMR (300 MHz, $CDCl_3$, ppm) δ 7.84(dd, *J* = 8.0, 2.0 Hz, 1H, Ar–H), 7.73 (dd, *J* = 8.0, 1.0 Hz, 1H, Ar–H), 7.50 (td, *J* = 8.0, 1.0 Hz, 1H), 7.43 (td, *J* = 8.0, 2.0 Hz, 1H), 1.88 (s, 2H, C=N$CH_2$). Analytical data (NMR) in agreement with those reported in the literature.[8]

**3-(4-Bromophenyl)-2*H*-azirine (2g)**

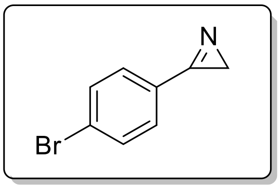

Prepared according General Procedure B using vinyl azide **1g** (224 mg). The product was obtained without further purification as a brown oil (192 mg, 98%). $^1$H NMR (500 MHz, $CDCl_3$, ppm) δ 7.77 (d, *J* = 8.4 Hz, 2H, 2 x Ar–H), 7.71 (d, *J* = 8.4 Hz, 2H, 2 x Ar–H), 1.81 (s, 2H, C=N$CH_2$). Analytical data (NMR) in agreement with those reported in the literature.[7]

**3-Octyl-2*H*-azirine (2h)**

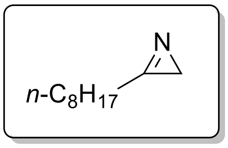

Prepared according General Procedure B (twice) using vinyl azide **1i** (181 mg). The product was obtained without further purification as a yellow oil (150 mg, 98%). $^1$H NMR (500 MHz, $CDCl_3$, ppm) δ $^1$H NMR (300 MHz, $CDCl_3$, ppm) δ 2.78 (t, *J* = 7.3 Hz, 2H, C$H_2$C=N), 1.74 (q, *J* = 7.3 Hz, 2H), 1.43–1.39 (m, 2H), 1.36 (s, 2H, C=NC$H_2$), 1.35–1.21 (broad signal, 8H), 0.88 (t, *J* = 6.9 Hz, 3H). $^{13}$C NMR (125 MHz, $CDCl_3$, ppm) δ 169.8 ($CH_2$*C*=N), 31.8, 29.2 (2 x $CH_2$), 29.1, 28.4, 24.1, 22.6, 18.8, 14.0 ($CH_3$). IR (ATR, neat)/cm$^{-1}$ 2955, 2925, 2856, 1466, 1260, 725. HRMS (ESITOF) m/z $(M+H)^+$ calcd for $C_{10}H_{20}N$ 154,1596; found 154,1591.

**3-Decyl-2*H*-azirine (2i)**

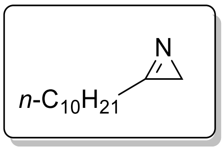

Prepared according General Procedure B using vinyl azide **1j** (209 mg). The product was obtained without further purification as a brown oil (175 mg, 97%). $^1$H NMR (500 MHz, $CDCl_3$, ppm) δ $^1$H NMR (300 MHz, $CDCl_3$, ppm) δ 2.78 (t, *J* = 7.3 Hz, 2H, C$H_2$C=N), 1.74 (q, *J* = 7.3 Hz, 2H), 1.43–1.20 (broad signal, 16H), 0.88 (t, *J* = 6.9 Hz, 3H). $^{13}$C NMR (125

MHz, $CDCl_3$, ppm) δ 169.9 ($CH_2$*C*=N), 32.0, 29.7, 29.6, 29.4 (3 × $CH_2$), 28.6, 24.3, 22.8, 18.9, 14.3 ($CH_3$). IR (ATR, neat)/$cm^{-1}$ 2956, 2926, 2856, 1672, 1460, 1378, 1261, 1035. HRMS (ESITOF) m/z $(M+H)^+$ calcd for $C_{12}H_{24}N$ 182,1909; found 182,1909.

**3-Dodecyl-2*H*-azirine (2j)**

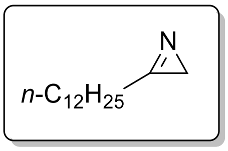


Prepared according General Procedure B using vinyl azide **1k** (237 mg). The product was obtained without further purification as a brown oil (203 mg, 97%). $^1H$ NMR (300 MHz, $CDCl_3$, ppm) δ 2.78 (t, *J* = 7.3 Hz, 2H, C*H*$_2$C=N), 1.74 (q, *J* = 7.3 Hz, 2H), 1.36–1.18 (broad signal, 20H), 0.88 (t, *J* = 6.9 Hz, 3H). $^{13}C$ NMR (75 MHz, $CDCl_3$, ppm) δ 169.9 ($CH_2$*C*=N), 34.0, 32.1, 29.8, 29.7, 29.6, 29.5, 29.4, 29.1, 28.6, 24.3, 22.8, 18.9, 14.2. IR (ATR, neat)/$cm^{-1}$ 2954, 2924, 2854, 1458, 986. HRMS (ESITOF) m/z $(M+H)^+$ calcd for $C_{14}H_{28}N$ 210,2222; found 210,2218.

**3-(Perfluorophenyl)-2*H*-azirine (2k)**

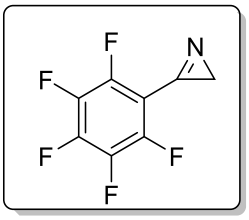


Prepared according General Procedure B. The product was obtained in mixture with vinyl azide **1l** (**1l**:**2l** = 20:80). $^1H$ NMR (500 MHz, $CDCl_3$, ppm) δ 1.85 (s, 2H, $NCH_2$). $^{19}F$ NMR (282 MHz, $CDCl_3$, ppm) δ -136.90 – -137.06 (m, 2F), -144.63 (tt, *J* = 20.6, 5.1 Hz, 1F), -159.71 – -159.97 (m, 2F).

**2,3-Diphenyl-2*H*-azirine (2l)**

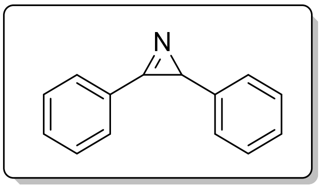


Prepared according General Procedure B. The product was obtained without any further purification as a yellow oil (192 mg, 99%). $^1H$ NMR (300 MHz, $CDCl_3$, ppm) δ 7.72–7.69 (m, 2H, Ar–H), 7.63–7.51 (m, 3H, Ar–H), 7.36 (t, *J* = 7.7 Hz, 1H), 7.30–7.27 (m, 2H, Ar–H), 7.17–7.15 (m, 2H, Ar–H), 3.33 (s, 1H, C=NCHPh). Analytical data (NMR) in agreement with those reported in the literature.[7]

## 4. General Procedure C

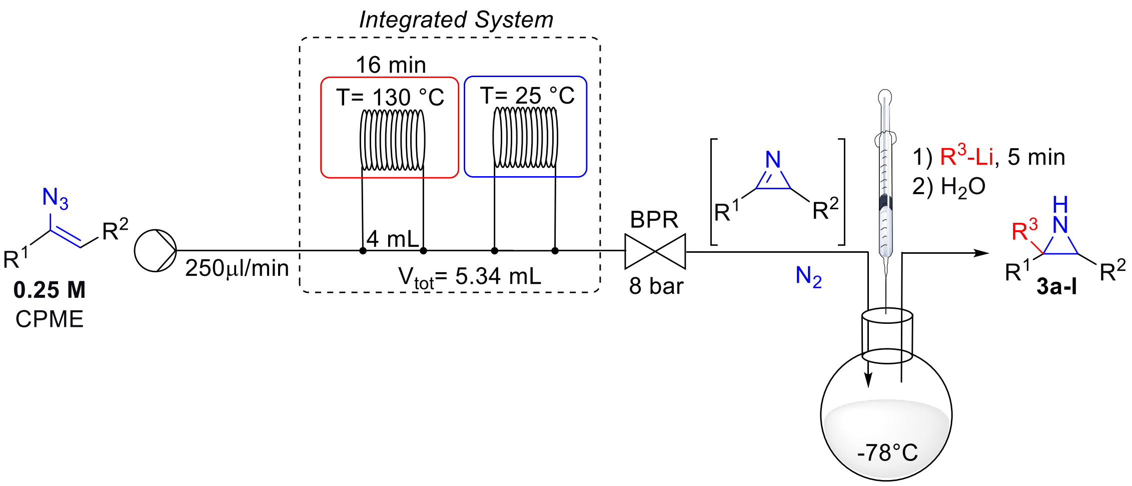


The process can be executed using a PHD ULTRA™ Syringe Pump (Harvard Apparatus), a Volcano reactor (4 mL, Syrris) and a back–pressure regulator of 8 bar. A solution of vinyl azide (1.0 mmol) in CPME (4 mL, 0.25 M) was introduced by syringe pump into the pre–heated reactor (130 °C, probe feedback control) with a flow rate of 250 µL/min. Subsequently, fresh solvent (CPME) was fluxed in the reactor upon the same conditions. The outgoing solution was collected in a closed round bottom flask with nitrogen atmosphere for 16 min, since the formation of nitrogen was observed. The stirred solution was cooled to –78 °C and organolithium (1.2 equiv) was added in one portion. After 5 min, $H_2O$ (100 µL) was added, and the reaction mixture was stirred at room temperature. The solution was filtered on a $Na_2SO_4$ pad, the solvent was evaporated under reduced pressure, and the products were isolated through silica gel chromatography as described for each entry.

### Combined flow-batch system

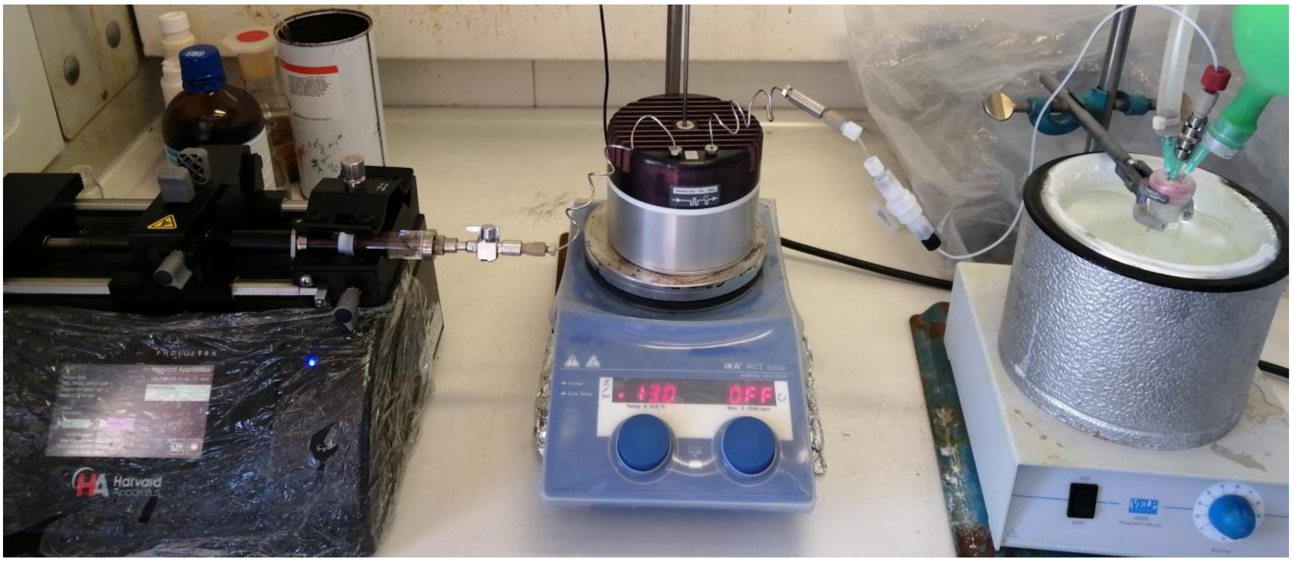

**Characterization data for aziridines 3a–l**

**2-Phenyl-2-(*p*-tolyl)aziridine (3a)**

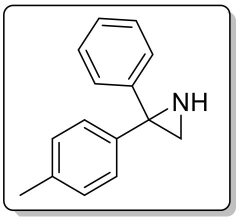


Prepared according General Procedure C using vinyl azide **1a**. The product was purified by column chromatography ($SiO_2$, $R_f$ 0.30, Hexane/Ethyl Acetate/ Triethylamine 80:19:1) to afford aziridine **3a** as a brown waxy solid (102 mg, 49%). $^1$H NMR (500 MHz, $CDCl_3$, ppm) δ 7.37–7.28 (m, 5H, Ar–H), 7.24 (d, *J* = 8.0 Hz, 2H, Ar–H), 7.13 (d, *J* = 8.0 Hz, 2H, Ar–H), 2.38 and 2.34 (2 x s, 2H, 2 x C=NC*H*H), 2.34 (s, 3H, $CH_3$). $^{13}$C NMR (125 MHz, $CDCl_3$, ppm) δ 143.0 (Ar–$C_q$), 139.8 (Ar–$C_q$), 137.0 (Ar–$C_q$), 129.2 (Ar), 127.8 (2 x Ar), 127.8 (Ar), 127.2 (Ar), 43.9 ($C_q$), 35.5 ($NCH_2$), 21.2 (Ar–$CH_3$). IR (ATR, neat)/cm$^{-1}$ 3293, 3026, 2920, 1657, 1446, 808, 698. HRMS (ESITOF) m/z $(M+H)^+$ calcd for $C_{15}H_{16}N$ 210,1283; found 210,1286.

**2-Hexyl-2-(*p*-tolyl)aziridine (3b)**

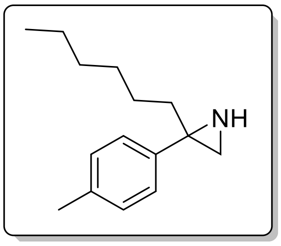


Prepared according General Procedure C using vinyl azide **1a**. The product was purified by column chromatography ($SiO_2$, $R_f$ 0.45 Hexane/Ethyl Acetate/Triethylamine 70:29:1) to afford aziridine **3b** as a brown waxy solid (109 mg, 50%). $^1$H NMR (500 MHz, $CDCl_3$, ppm) δ 7.25 (d, *J* = 8.0 Hz, 2H, Ar–H), 7.12 (d, *J* = 8.0 Hz, 2H, Ar–H), 2.33 (s, 3H, Ar–$CH_3$), 1.91 and 1.85 (2 x s, 2H, 2 x C=NC*H*H), 1.81–1.75 (m, 1H), 1.72–1.67(m, 1H), 1.29–1.21 (m, 8H), 0.84 (t, *J* = 6.9 Hz, 3H, $CH_2CH_3$). $^{13}$C NMR (125 MHz, $CDCl_3$, ppm) δ 139.7 (Ar–$C_q$), 136.7 (Ar–$C_q$), 129.1 (2 x Ar), 127.6 (2 x Ar), 41.7 ($C_q$), 39.6 ($CH_2C{=}NCH_2$), 33.1 ($CH_2C{=}NCH_2$), 31.9 ($CH_2$), 29.5 ($CH_2$), 26.3 ($CH_2$), 22.7($CH_2$), 21.2 (Ar–$CH_3$), 14.2 ($CH_3$). IR (ATR, neat)/cm$^{-1}$ 3291, 2954, 2856, 1676, 1464, 815. HRMS (ESITOF) m/z $(M+H)^+$ calcd for $C_{15}H_{24}N$ 218,1909; found 218,1914.

**2-Butyl-2-(*p*-tolyl)aziridine (3c)**

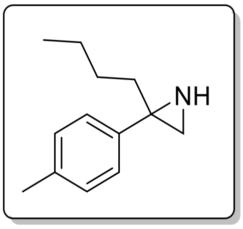

Prepared according General Procedure C using vinyl azide **1a**. The product was purified by column chromatography ($SiO_2$, $R_f$ 0.30 Hexane/Ethyl Acetate/Triethylamine 70:29:1) to afford aziridine **3c** as a brown waxy solid (87 mg, 46%). $^1$H NMR (500 MHz, $CDCl_3$, ppm) δ 7.26(d, *J* = 7.6 Hz, 2H, Ar–H), 7.12 (d, *J* = 7.6 Hz, 2H, Ar–H), 2.33 (s, 3H, Ar–$CH_3$), 1.92 and 1.85 (2 x s, 2H, 2 x C=NC*H*H), 1.80–1.77 (m, 1H), 1.73–1.69(m, 1H), 1.28–1.26 (m, 4H), 0.84 (t, *J* = 6.9 Hz, 3H, $CH_2CH_3$). $^{13}$C NMR (125 MHz, $CDCl_3$, ppm) δ 139.7 (Ar–$C_q$), 136.7 (Ar–$C_q$), 129.1 (2 x Ar), 127.6 (2 x Ar), 41.7 ($C_q$), 39.3 (*C*$H_2$C=N$CH_2$), 33.0 ($CH_2$C=N*C*$H_2$), 28.5 ($CH_2$), 22.9 ($CH_2$), 21.2 (Ar–$CH_3$), 14.2 ($CH_3$). IR (ATR, neat)/$cm^{-1}$ 3296, 2956, 2928, 2859, 1517, 1458, 817, 561. HRMS (ESITOF) m/z $(M+H)^+$ calcd for $C_{13}H_{20}N$ 190,1596; found 190,1596.

**2-Isobutyl-2-(*p*-tolyl)aziridine (3d)**

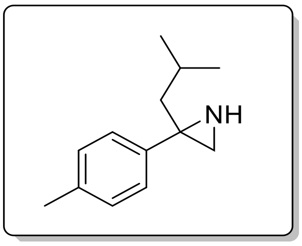


Prepared according General Procedure C using vinyl azide **1a**. The product was purified by column chromatography ($SiO_2$, $R_f$ 0.45 Hexane/Ethyl Acetate/Triethylamine 50:49:1) to afford aziridine **3d** as a brown waxy solid (89 mg, 47%). $^1$H NMR (300 MHz, $CDCl_3$, ppm) δ 7.29(d, *J* = 8.0 Hz, 2H, Ar–H), 7.12 (d, *J* = 8.0 Hz, 2H, Ar–H), 2.33 (s, 3H, Ar–$CH_3$), 1.95 and 1.84 (2 x s, 2H, 2 x C=NC*H*H), 1.69–1.67 (m, 2H), 1.52 (nonet, *J*= 6.7 Hz, 1H, C*H*$(CH_3)_2$), 1.07 (broad signal,1H, NH), 0.89 and 0.88 (2 x d, *J* = 6.5 Hz, 6H, 2 x CH(C*H*$_3$). $^{13}$C NMR (75 MHz, $CDCl_3$, ppm) δ 139.6 (Ar–$C_q$), 136.6 (Ar–$C_q$), 129.1 (2 x Ar), 127.6 (2 x Ar), 48.9 ($C_q$), 40.6 (*C*$H_2$C=N$CH_2$), 32.8 ($CH_2$C=N*C*$H_2$), 26.2 ($CH_2$), 23.3 (CH*C*$H_3$), 22.9 (CH*C*$H_3$) 21.2 (Ar–$CH_3$). IR (ATR, neat)/$cm^{-1}$ 3293, 3052, 2953, 2868, 1517, 1467, 813, 804, 560. HRMS (ESITOF) m/z $(M+H)^+$ calcd for $C_{13}H_{20}N$ 190,1596; found 190,1596.

**2-Phenyl-2-(*o*-tolyl)aziridine (3e)**

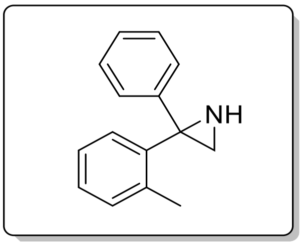


Prepared according General Procedure C using vinyl azide **1d**. The product was purified by column chromatography ($SiO_2$, $R_f$ 0.55 Hexane/Ethyl Acetate/Triethylamine 50:49:1) to afford aziridine **3e** as a brown oil (109 mg, 52%). $^1$H NMR (500 MHz, $CDCl_3$, ppm) δ 7.52–7.51 (m, 1H, Ar–H), 7.27–7.23 (m, 4H, Ar–H), 7.21–7.15 (m, 4H, Ar–H), 2.45 and 2.34 (2 x s, 2H, 2 x C=NC*H*H), 2.24 (s, 3H, Ar–$CH_3$). $^{13}$C NMR (125 MHz, $CDCl_3$, ppm) δ 142.3 (Ar–$C_q$), 140.1 (Ar–$C_q$), 138.1 (Ar–$C_q$), 130.5 (Ar), 129.5 (Ar), 128.3 (Ar), 127.8 (Ar), 126.6 (Ar), 125.9 (Ar), 125.8 (Ar), 42.8 ($C_q$), 36.5, 19.6 (Ar–$CH_3$). IR (ATR, neat)/$cm^{-1}$ 3280, 3060, 2854, 1639, 1494, 755, 698. HRMS (ESITOF) m/z $(M+H)^+$ calcd for $C_{15}H_{16}N$ 210,1283; found 210,1281.

**2-Hexyl-2-(*o*-tolyl)aziridine (3f)**

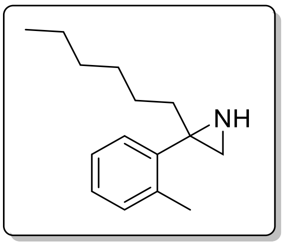


Prepared according General Procedure C using vinyl azide **1d**. The product was purified by column chromatography ($SiO_2$, $R_f$ 0.35 Hexane/Ethyl Acetate/Triethylamine 70:29:1) to afford aziridine **3f** as a brown waxy solid (134 mg, 62%). $^1$H NMR (500 MHz, $CDCl_3$, ppm) δ 7.29–7.26 (m, 1H, Ar–H), 7.18–7.12 (m, 3H, Ar–H), 2.43 (s, 3H, Ar–$CH_3$), 1.93 and 1.89 (2 $^x$ s, 2H, 2 $^x$ C=NC*H*H), 1.74–1.79 (m, 1H, CH*H*C=$NCH_2$), 1.64–1.59 (m, 1H, CH*H*C=$NCH_2$), 1.31–1.21 (broad signal, 8H), 0.84 (t, *J* = 6.9 Hz, 3H, $CH_2CH_3$). $^{13}$C NMR (125 MHz, $CDCl_3$, ppm) δ 140.8 (Ar–$C_q$), 136.7 (Ar–$C_q$), 130.4 (Ar), 129.4 (Ar), 127.2 (Ar), 125.6 (Ar), 41.7 ($C_q$), 38.6 (*C*$H_2$C=$NCH_2$), 32.7 ($CH_2$C=N*C*$H_2$), 31.9 ($CH_2$), 29.5 ($CH_2$), 26.2 ($CH_2$), 22.7 ($CH_2$), 19.3 (Ar–$CH_3$), 14.2 ($CH_3$). IR (ATR, neat)/cm$^{-1}$ 3282, 3058, 2927, 1687, 1457, 878, 760, 729. HRMS (ESITOF) m/z $(M+H)^+$ calcd for $C_{15}H_{24}N$ 218,1909; found 218,1907.

**2-Butyl-2-(*o*-tolyl)aziridine (3g)**

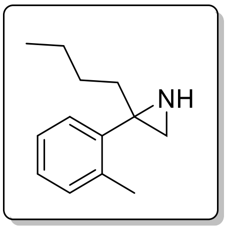


Prepared according General Procedure C using vinyl azide **1d**. The product was purified by column chromatography ($SiO_2$, $R_f$ 0.35 Hexane/Ethyl Acetate/Triethylamine 60:39:1) to afford aziridine **3g** as a brown waxy solid (97 mg, 51%). $^1$H NMR (500 MHz, $CDCl_3$, ppm) δ 7.30–7.26 (m, 1H, Ar–H), 7.18–7.13 (m, 3H, Ar–H), 2.43 (s, 3H, Ar–$CH_3$), 1.93 and 1.89 (2 $^x$ s, 2H, 2 $^x$ C=NC*H*H), 1.76–1.62 (m, 2H), 1.33–1.21 (m, 4H), 0.83 (t, *J* = 6.9 Hz, 3H, $CH_2CH_3$). $^{13}$C NMR (125 MHz, $CDCl_3$, ppm) δ 140.8 (Ar–$C_q$), 136.8 (Ar–$C_q$), 130.4 (Ar), 129.5 (Ar), 127.2 (Ar), 125.6 (Ar), 41.7 ($C_q$), 38.4 (*C*$H_2$C=$NCH_2$), 32.7 ($CH_2$C=N*C*$H_2$) 28.4 ($CH_2$), 23.0 ($CH_2$), 19.3 (Ar–$CH_3$), 14.2 ($CH_3$). IR (ATR, neat)/cm$^{-1}$ 3298, 2956, 2930, 2858, 1491, 1458, 862, 761, 730. HRMS (ESITOF) m/z $(M+H)^+$ calcd for $C_{13}H_{20}N$ 190,1596; found 190,1591.

**2-Decyl-2-phenylaziridine (3h)**

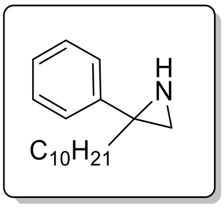


Prepared according General Procedure C using vinyl azide **1i**. The product was purified by column chromatography ($SiO_2$, $R_f$ 0.45 Hexane/Ethyl Acetate/Triethylamine 60:39:1) to

afford aziridine **3h** as a yellow waxy solid (116 mg, 45%). $^{1}$H NMR (500 MHz, $CDCl_3$, ppm) δ 7.37 (d, J = 7.1 Hz, 2H, Ar–H), 7.31 (t, *J* = 7.5 Hz, 2H, Ar–H), 7.24 (t, J = 7.3 Hz, 1H, Ar–H), 1.94 and 1.90 (2 × s, 2H, 2 × NC*H*H), 1.84–1.79 (m, 1H), 1.74–1.68 (m, 1H), 1.29–1.21 (broad signal, 16H), 0.87 (t, J = 6.5 Hz, 3H, $CH_2CH_3$). $^{13}$C NMR (125 MHz, $CDCl_3$, ppm) δ 142.6 (Ar–$C_q$), 128.4 (Ar), 127.7 (Ar), 127.1 (Ar), 42.1 ($C_q$), 39.6 (*C*H2$C_q$PhN), 32.0 ($C_q$N*C*$H_2$), 29.8 ($CH_2$), 29.7 (2 × $CH_2$), 29.6 (2 × $CH_2$), 29.4 (2 × $CH_2$), 29.3 ($CH_2$), 14.3 ($CH_3$). IR (ATR, neat)/cm$^{-1}$ 3298, 3059, 2923, 2852, 1465, 867, 698. HRMS (ESITOF) m/z $(M+H)^+$ calcd for $C_{18}H_{30}N$ 260,2378; found 260,2380.

**2-Dodecyl-2-isobutylaziridine (3i)**

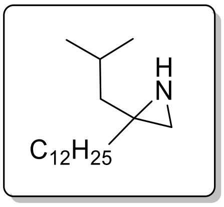


Prepared according General Procedure C using vinyl azide **1j**. The product was purified by column chromatography ($SiO_2$, $R_f$ 0.30 Hexane/Ethyl Acetate/Triethylamine 50:49:1) to afford aziridine **3i** as a yellow waxy solid (128 mg, 48%). $^{1}$H NMR (500 MHz, $CDCl_3$, ppm) δ 1.81–1.73 (m, 1H), 1.52 and 1.51 (2 × s, 2H, 2 × NC*H*H), 1.47 and 1.14 (2 × dd, *J* = 14.0, 8.0 Hz, 2H, 2 × C*H*HCH($CH_3$)$_2$), 1.34–1.19 (broad signal, 22H), 0.94 and 0.92 (2 × d, *J* = 6.6 Hz, 6H, 2 × CHC*H*$_3$), 0.87 (t, *J* = 6.8 Hz, 3H, $CH_2CH_3$). $^{13}$C NMR (125 MHz, $CDCl_3$, ppm) δ 45.5, 37.0, 35.7, 32.4, 32.1, 30.0, 29.8 (5 × $CH_2$), 29.5, 25.9, 25.8, 23.5, 22.9, 22.8, 14.3 ($CH_3$). IR (ATR, neat)/cm$^{-1}$ 2954, 2923, 2853, 1466, 1378, 801. HRMS (ESITOF) m/z $(M+H)^+$ calcd for $C_{18}H_{37}N$ 268,3004; found 268,3005.

**2,2,3-Triphenylaziridine (3j)**

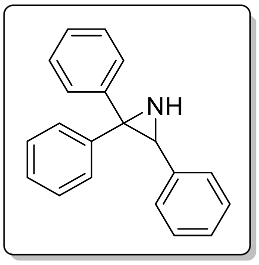


Prepared according General Procedure C using vinyl azide **1l**. The product was purified by column chromatography ($SiO_2$, $R_f$ 0.60 Hexane/Ethyl Acetate 90:10) to afford aziridine **3j** as a white solid (168 mg, 62%). $^{1}$H NMR (500 MHz, $CDCl_3$, ppm) δ 7.41 (d, *J* = 7.4 Hz, 2H, Ar–H), 7.35 (t, *J* = 7.5 Hz, 2H, Ar–H), 7.28 (d, *J* = 7.4 Hz, 1H, Ar–H), 7.25 (d, *J* = 7.0 Hz, 2H, Ar–H), 7.16–7.11 (m, 8H, Ar–H), 3.90 (s, 1H, NCHPh), 1.80 (NH). $^{13}$C NMR (75 MHz, $CDCl_3$, ppm) δ 144.4 (Ar–$C_q$), 138.5 (Ar–$C_q$), 137.2 (Ar–$C_q$), 129.8 (Ar), 128.8 (Ar), 127.8 (2 × Ar), 127.6 (Ar), 127.4 (Ar), 127.1 (Ar), 126.8 (2 × Ar), 52.4, 47.0. Analytical data (NMR) in agreement with those reported in the literature.[9]

**(2*S**,3*R**)-2-Hexyl-2,3-diphenylaziridine (3k)**

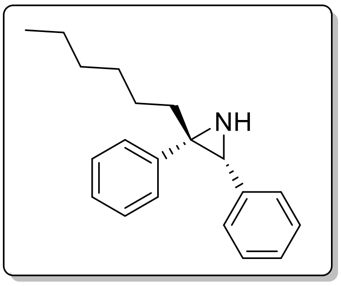


Prepared according General Procedure C using vinyl azide **1l**. The product was purified by column chromatography ($SiO_2$, $R_f$ 0.50 Hexane/Ethyl Acetate 80:20) to afford aziridine **3k** as a pale yellow waxy solid (145 mg, 52%). $^1$H NMR (500 MHz, $CDCl_3$, ppm) δ 7.17–7.12 (m, 4H, Ar–H), 7.10–7.02 (m, 4H, Ar–H), 6.96–6.94 (m, 2H, Ar–H), 3.30 (s, 1H, C=NCHPh), 2.21–2.15 and 1.68–1.62 (2 × m, 2H, 2 × C*H*HC=N), 1.51 (broad signal, 1H, NH), 1.41–1.19 (broad signal, 8H), 0.86 (t, *J* = 7.0 Hz, 3H). $^{13}$C NMR (125 MHz, $CDCl_3$, ppm) δ 138.7 (Ar–$C_q$), 137.9 (Ar–$C_q$), 129.3 (2 × Ar–C), 127.7 (2 × Ar–C) 127.6 (2 × Ar–C), 127.2 (2 × Ar–C), 126.5 (Ar–C), 127.4 (Ar–C), 50.8 ($C_q$), 45.6 (N*C*HPh), 42.9 (PhC*C*$H_2$$CH_2$), 31.9, 29.5, 26.2, 22.7, 14.2 ($CH_3$). IR (ATR, neat)/cm$^{-1}$ 3298, 3028, 2927, 2855, 1603, 1447, 752, 696. HRMS (ESITOF) m/z $(M+H)^+$ calcd for $C_{20}H_{26}N$ 280,2065; found 280,2053.

**(2*S**,3*R**)-2-Isobutyl-2,3-diphenylaziridine (3l)**

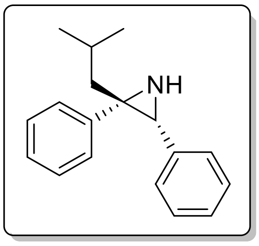


Prepared according General Procedure C using vinyl azide **1l**. The product was purified by column chromatography ($SiO_2$, $R_f$ 0.25 Hexane/AcOEt/$Et_3N$ 90:9:1) to afford aziridine **3l** as a pale yellow waxy solid (113 mg, 45%). $^1$H NMR (500 MHz, $CDCl_3$, ppm) δ 7.18 (d, *J* = 7.0 Hz, 2H, Ar–H), 7.13 (t, *J* = 7.0 Hz, 2H, Ar–H), 7.09–7.03 (m, 4H, Ar–H), 6.97 (d, *J* = 7.0 Hz, 2H, Ar–H), 3.25 (s, PhCHN, 1H), 2.21 (dd, *J* = 14.0, 6.0 Hz,1 × PhCC*H*HCH($CH_3$)$_2$, 1H), 1.59–1.47 (m, 2H), 0.98 and 0.94 (2 × d, *J* = 6.5 Hz, 2 × PhCC$H_2$CH(C*H*$_3$)$_2$, 6H). $^{13}$C NMR (75 MHz, $CDCl_3$, ppm) δ 138.3 (Ar–$C_q$), 137.5 (Ar–$C_q$), 129.1 (Ar), 127.5 (Ar), 127.4 (Ar), 127.1 (Ar), 126.3 (2 × Ar), 52.0 (Ph$C_q$*C*$H_2$CH($CH_3$)$_2$), 49.6 (Ph*C*$_q$$CH_2$CH($CH_3$)$_2$), 45.0 (PhCHN), 26.1 (Ph$C_q$$CH_2$*C*H($CH_3$)$_2$), 23.2 and 22.7 (2 × CH(*C*$H_3$)$_2$). IR (ATR, neat)/cm$^{-1}$ 3086, 3028, 2926, 2869, 1683, 1498, 871, 697, 607. HRMS (ESITOF) m/z $(M+H)^+$ calcd for $C_{18}H_{22}N$ 252,1752; found 252,1756.

## 5. Copies of $^{1}H$, $^{13}C$, $^{19}F$, NOESY NMR spectra

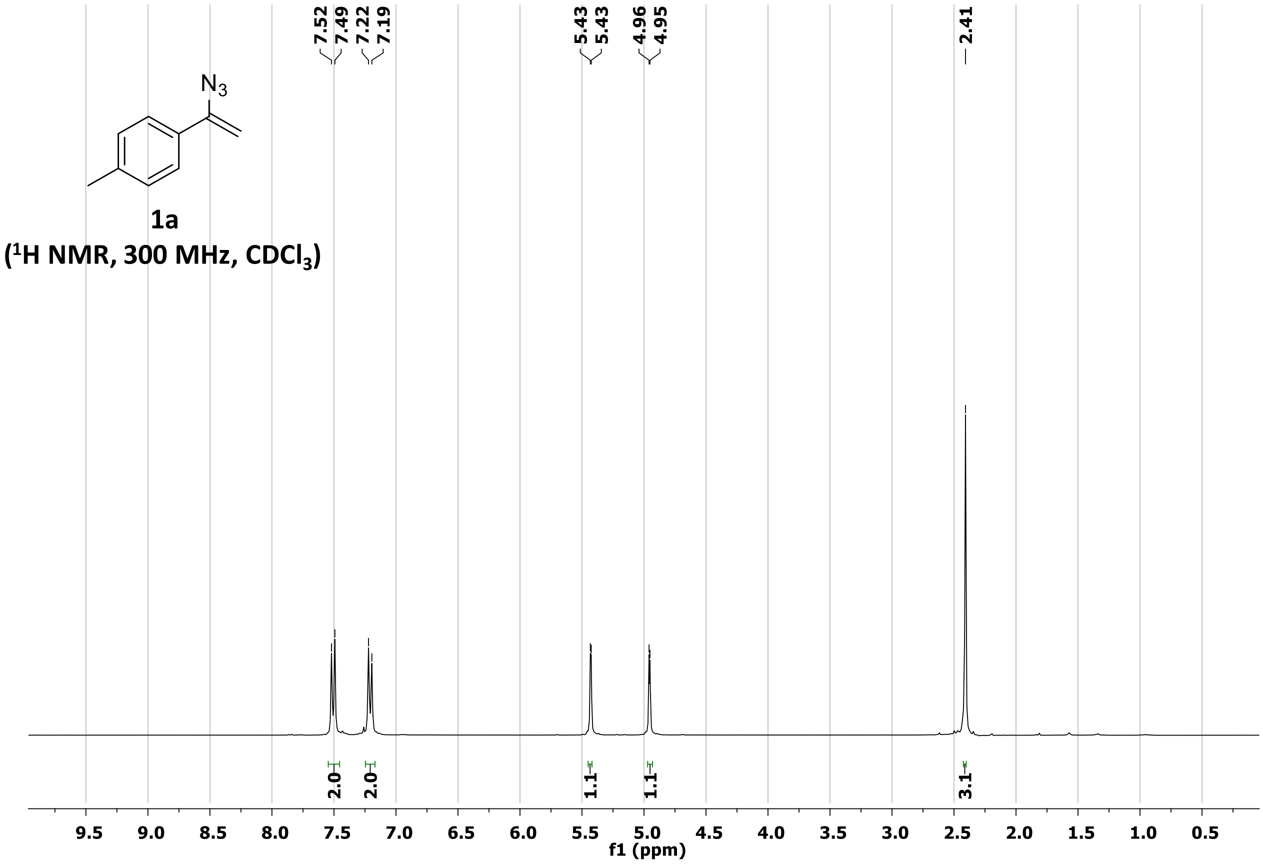


**1a**

**($^{1}H$ NMR, 300 MHz, $CDCl_3$)**

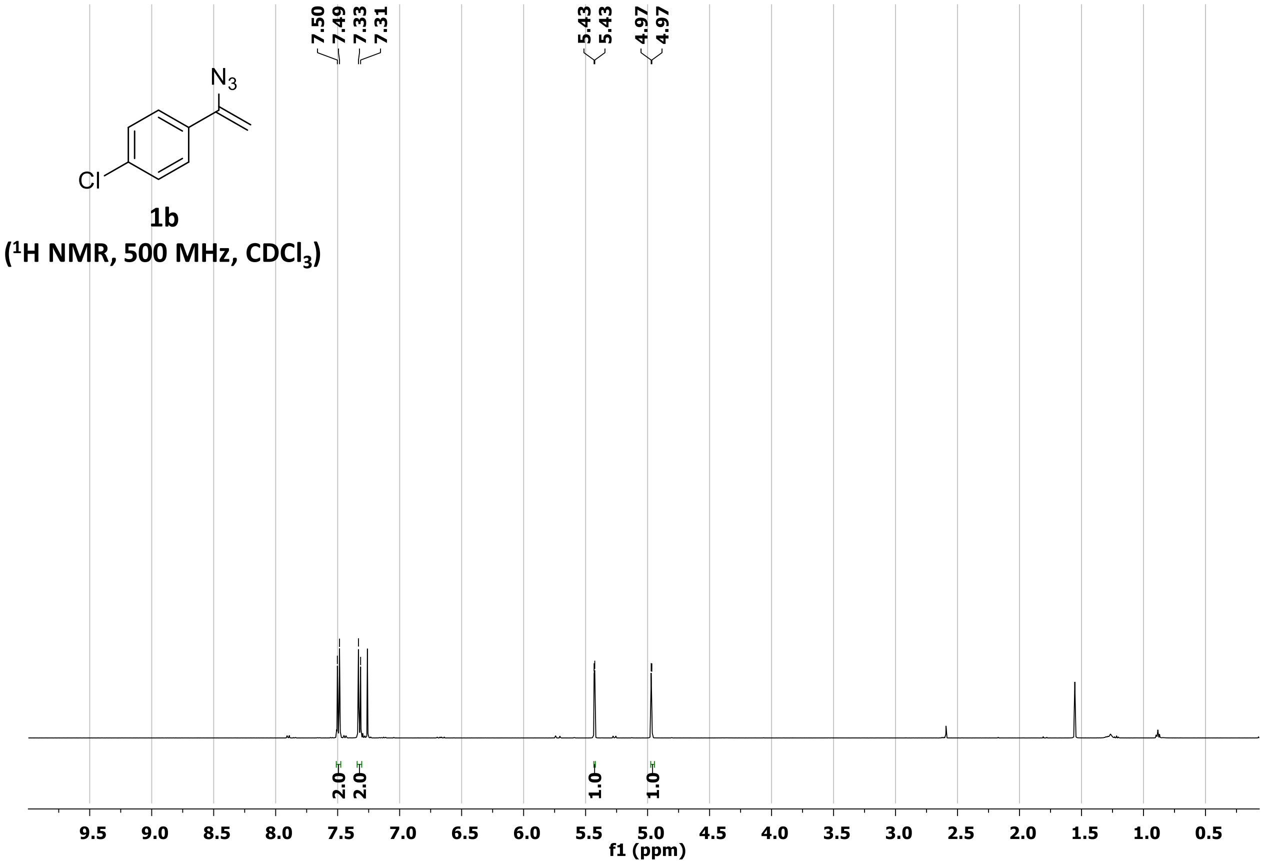


**1b**

**($^{1}H$ NMR, 500 MHz, $CDCl_3$)**

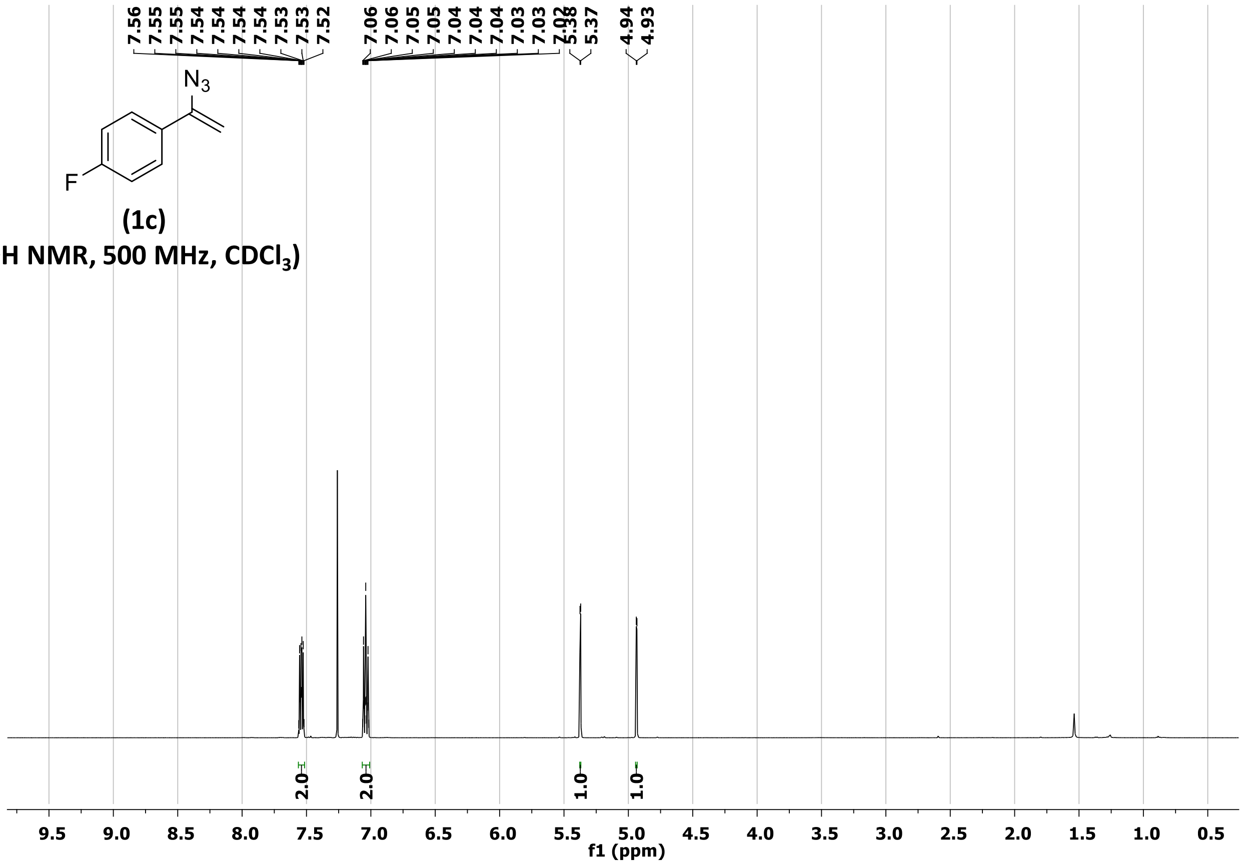


**(1c)**
**($^1$H NMR, 500 MHz, $CDCl_3$)**

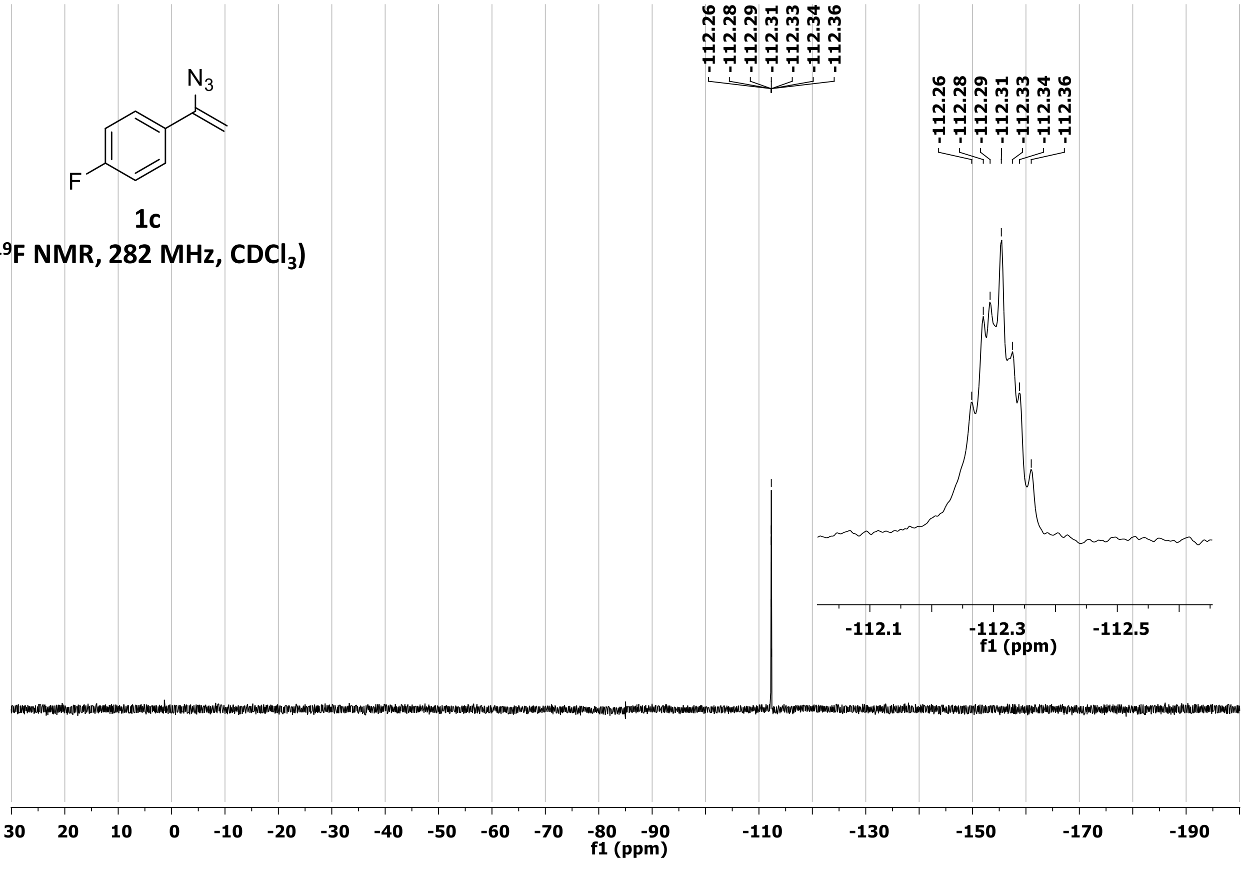


**1c**
**($^{19}$F NMR, 282 MHz, $CDCl_3$)**

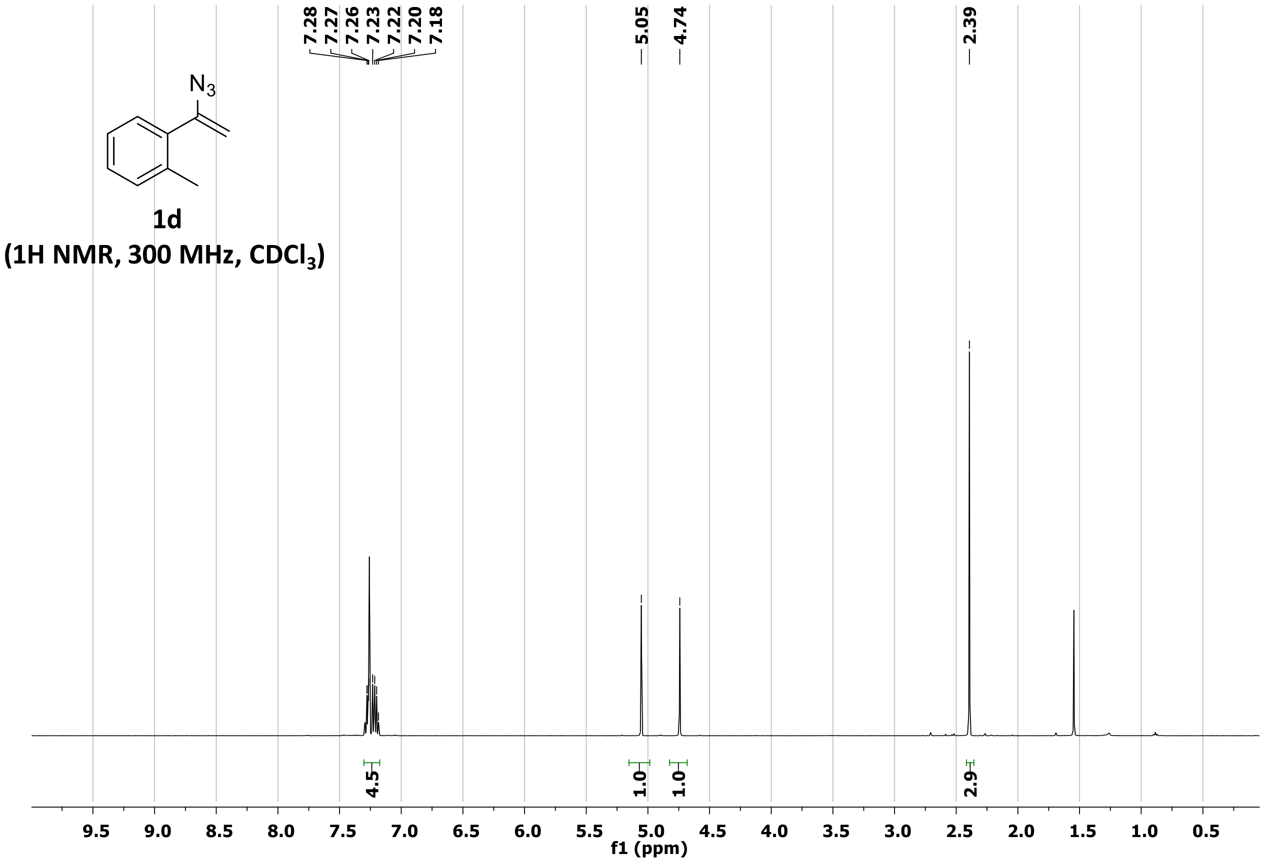
7.28
7.27
7.26
7.23
7.22
7.20
7.18
5.05
4.74
2.39
$N_3$
1d
(1H NMR, 300 MHz, $CDCl_3$)
4.5
1.0
1.0
2.9
9.5 9.0 8.5 8.0 7.5 7.0 6.5 6.0 5.5 5.0 4.5 4.0 3.5 3.0 2.5 2.0 1.5 1.0 0.5
f1 (ppm)

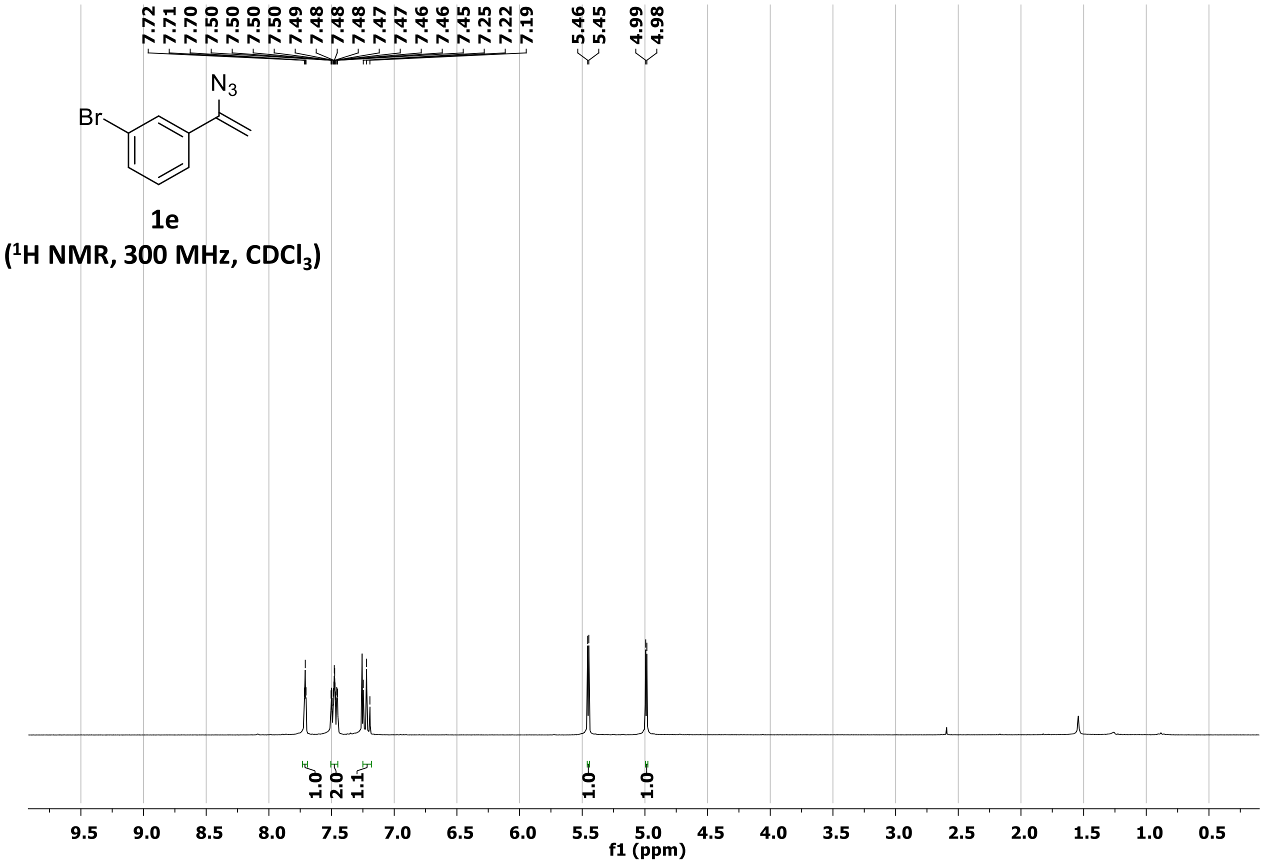
7.72
7.71
7.70
7.50
7.50
7.50
7.49
7.48
7.48
7.48
7.47
7.46
7.46
7.45
7.25
7.22
7.19
5.46
5.45
4.99
4.98
Br
$N_3$
1e
($^1$H NMR, 300 MHz, $CDCl_3$)
1.0
2.0
1.1
1.0
1.0
9.5 9.0 8.5 8.0 7.5 7.0 6.5 6.0 5.5 5.0 4.5 4.0 3.5 3.0 2.5 2.0 1.5 1.0 0.5
f1 (ppm)

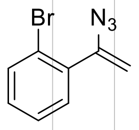

**1f**

**($^{1}$H NMR, 500 MHz, $CDCl_3$)**

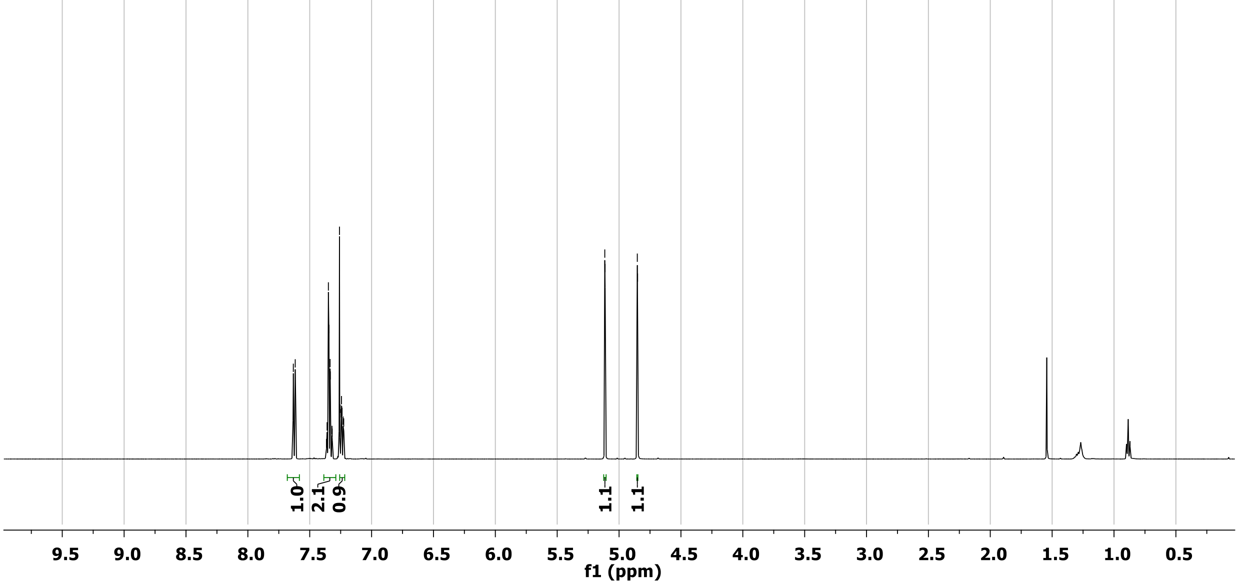


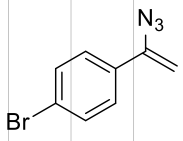

**1g**

**($^{1}$H NMR, 500 MHz, $CDCl_3$)**

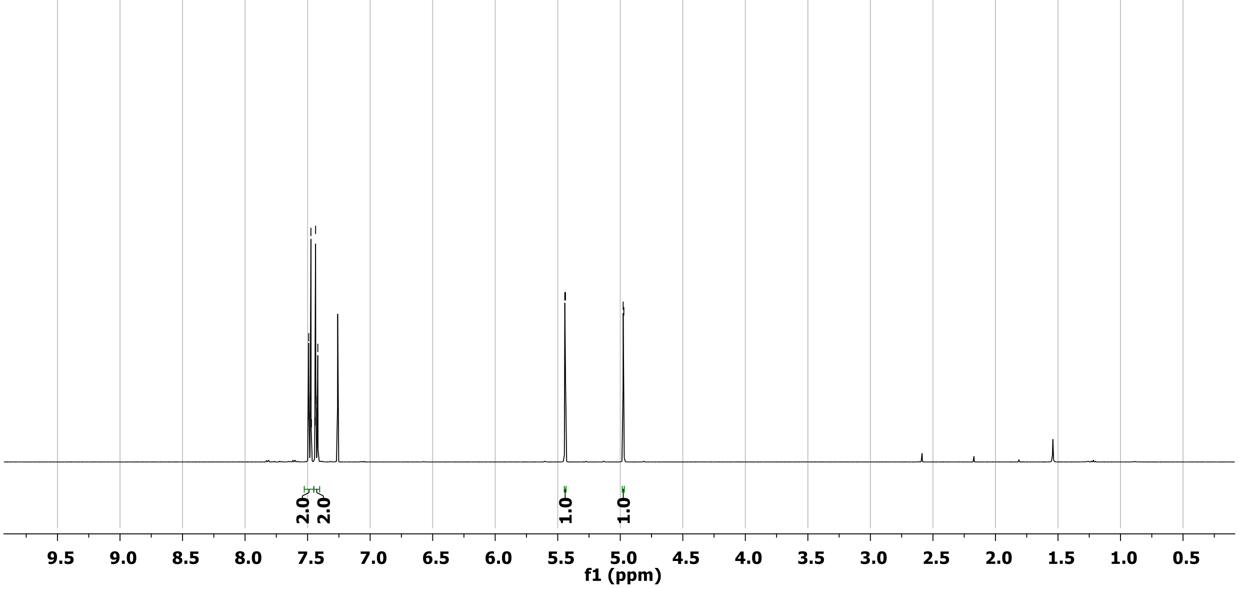

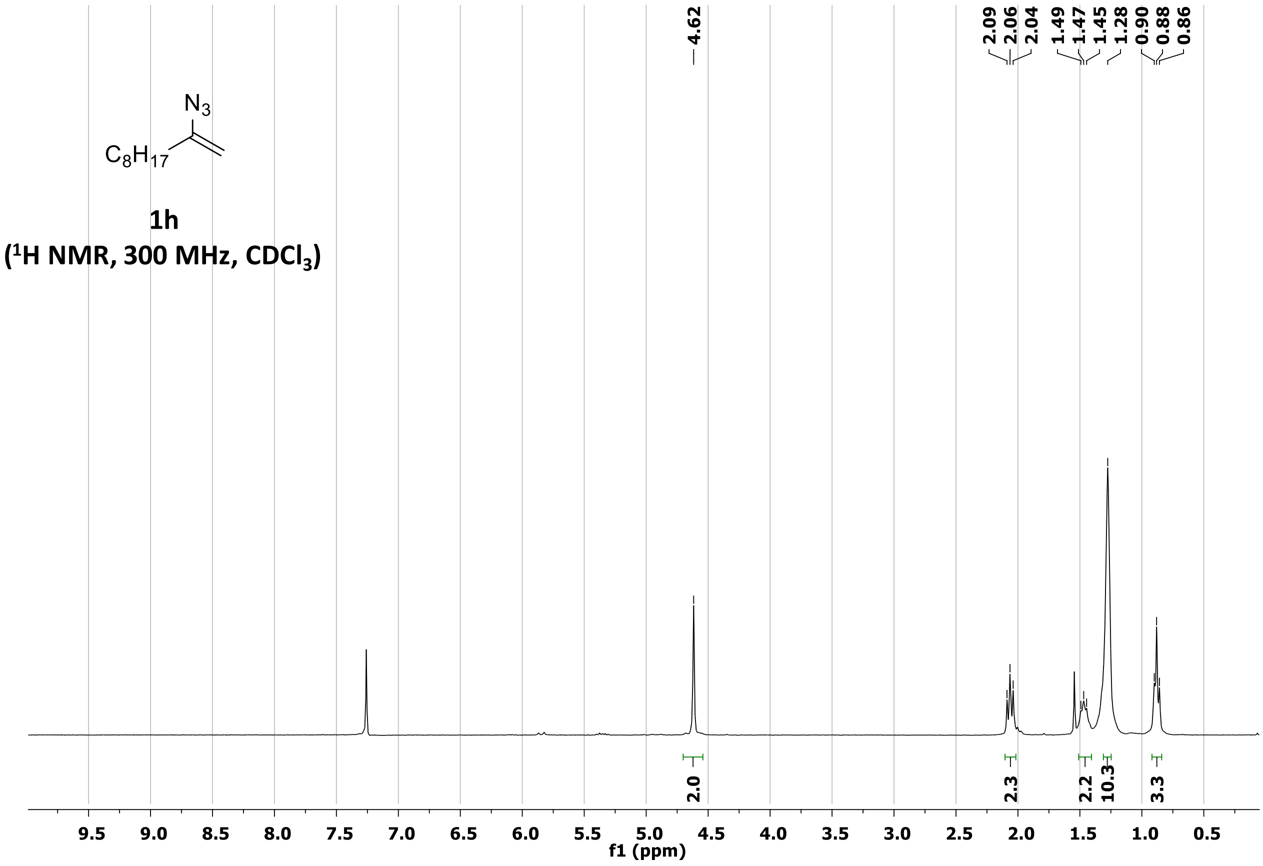


**1h**
**($^1$H NMR, 300 MHz, $CDCl_3$)**

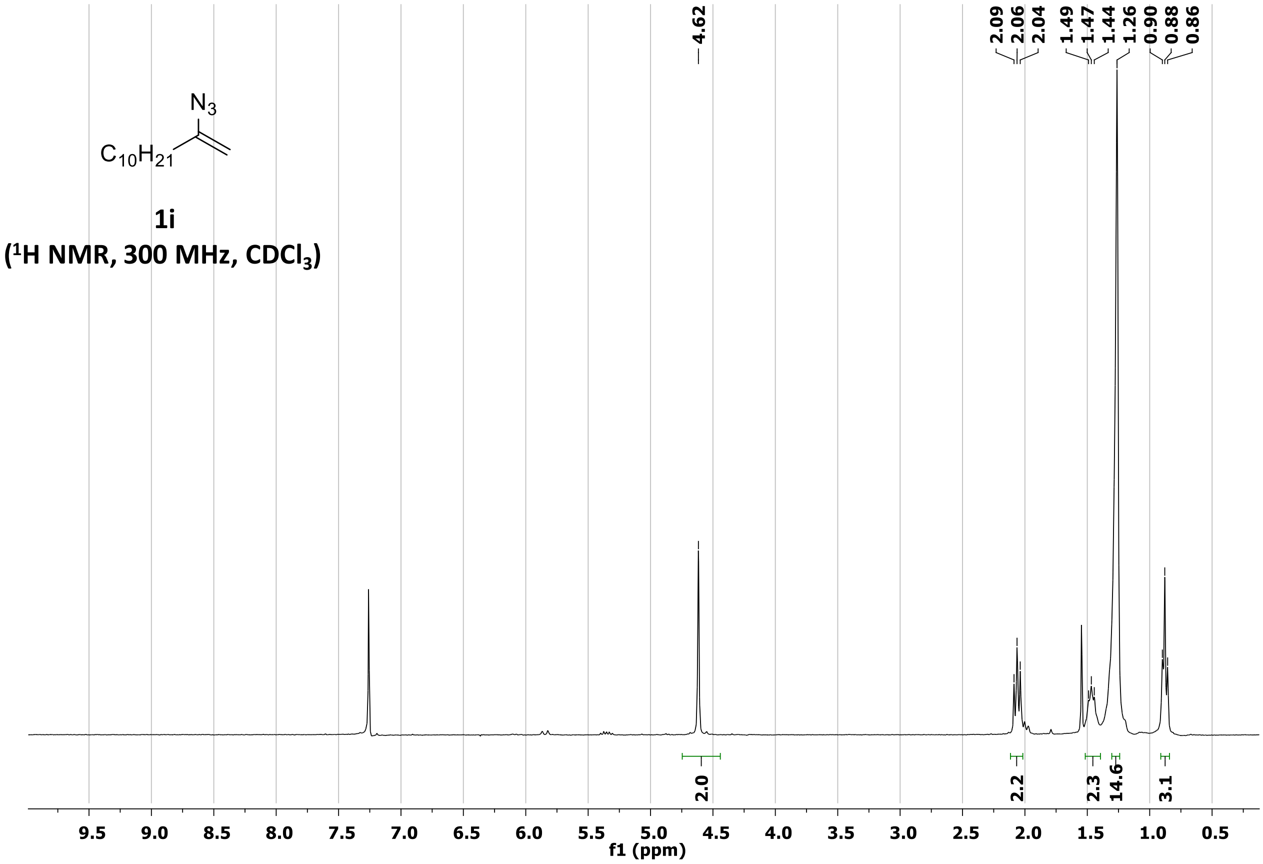


**1i**
**($^1$H NMR, 300 MHz, $CDCl_3$)**

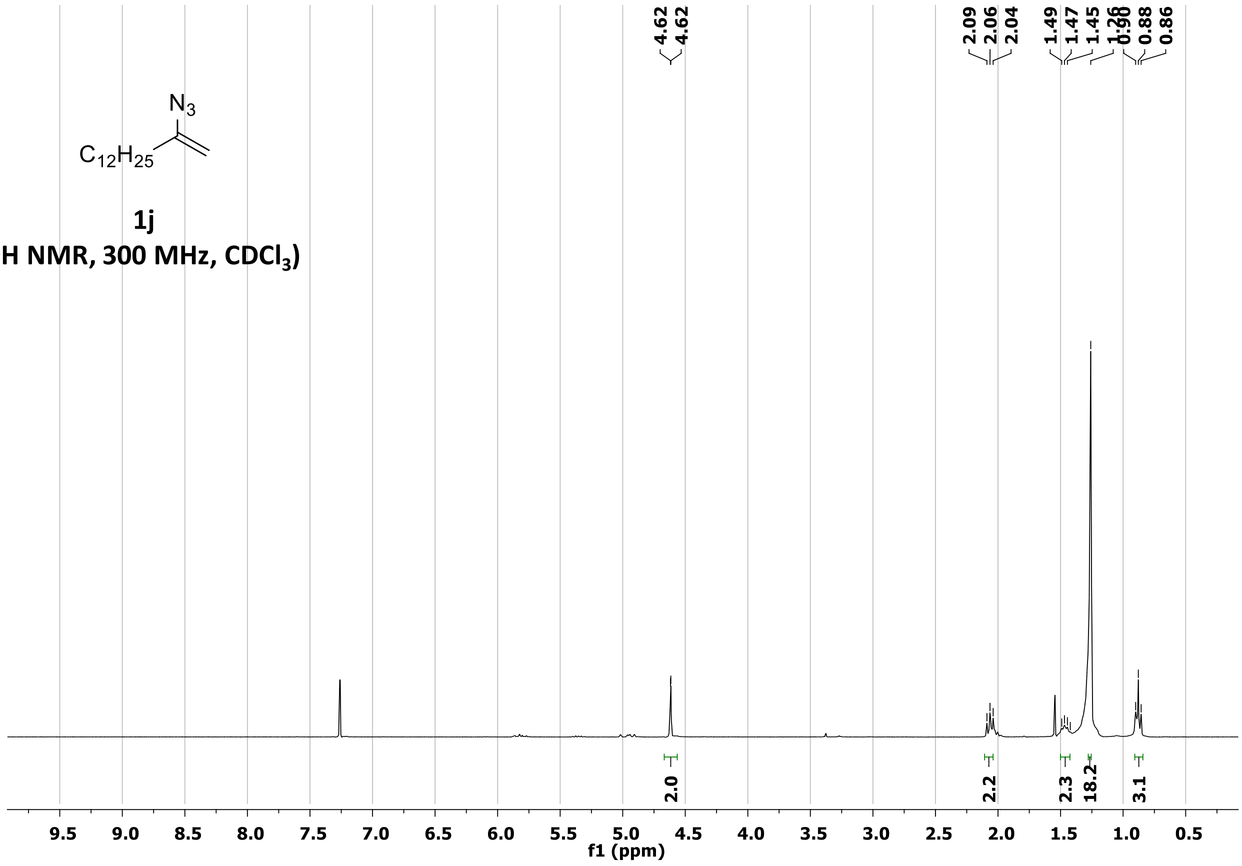


**1j**
**($^1$H NMR, 300 MHz, $CDCl_3$)**

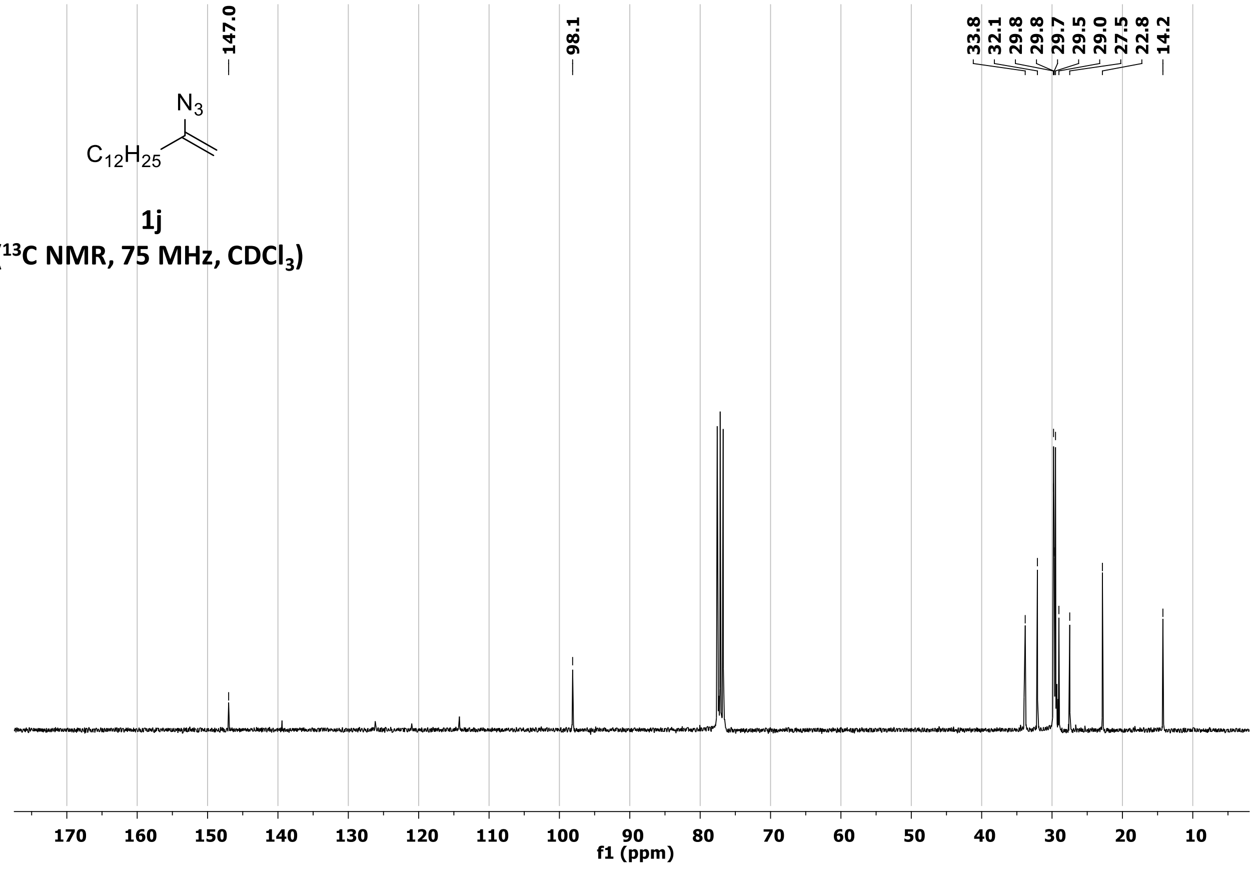


**1j**
**($^{13}$C NMR, 75 MHz, $CDCl_3$)**

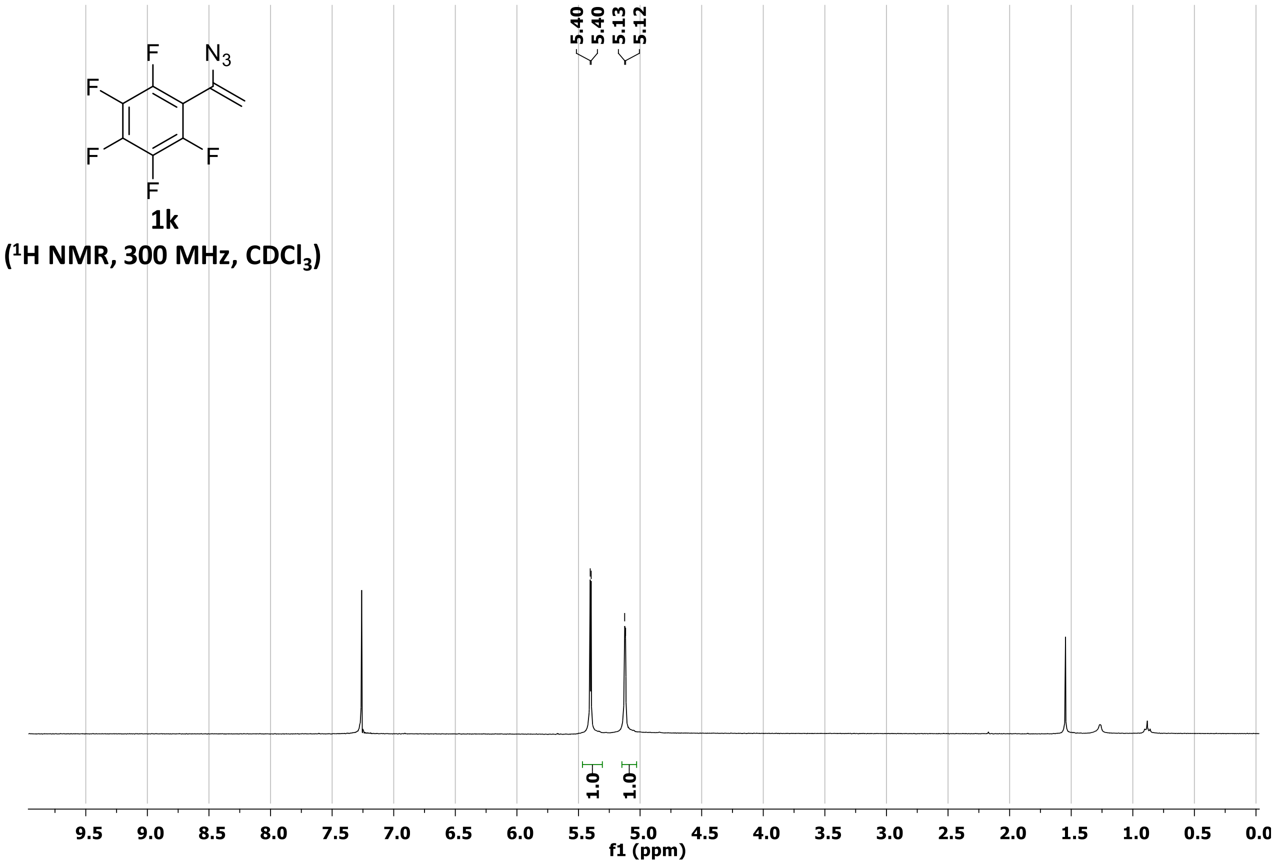
5.40
5.40
5.13
5.12
N3
F
F
F
F
F
1k
(1H NMR, 300 MHz, CDCl3)
1.0
1.0
9.5 9.0 8.5 8.0 7.5 7.0 6.5 6.0 5.5 5.0 4.5 4.0 3.5 3.0 2.5 2.0 1.5 1.0 0.5 0.0
f1 (ppm)

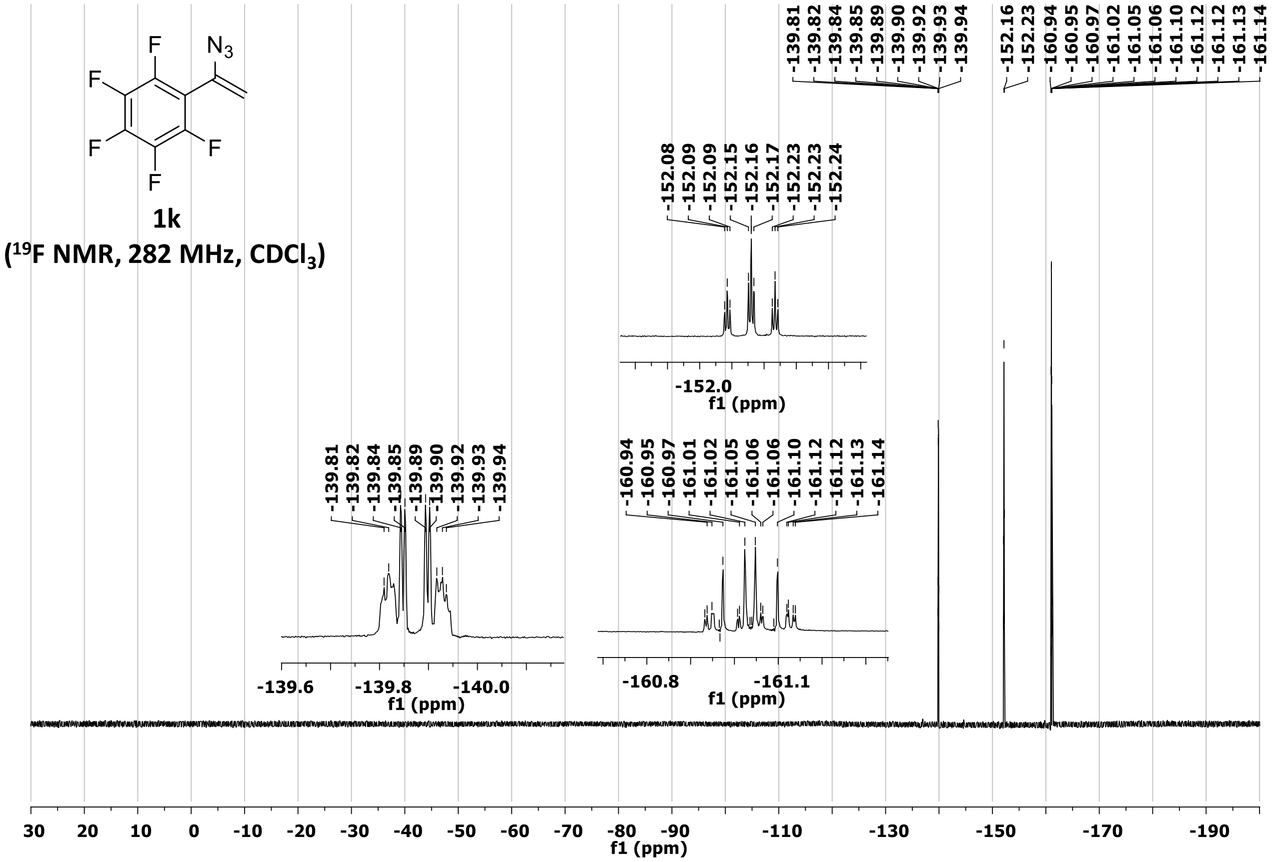
-139.81
-139.82
-139.84
-139.85
-139.89
-139.90
-139.92
-139.93
-139.94
-152.16
-152.23
-160.94
-160.95
-160.97
-161.02
-161.05
-161.06
-161.10
-161.12
-161.12
-161.13
-161.14
N3
F
F
F
F
F
1k
(19F NMR, 282 MHz, CDCl3)
-152.08
-152.09
-152.09
-152.15
-152.16
-152.17
-152.23
-152.23
-152.24
-152.0
f1 (ppm)
-139.81
-139.82
-139.84
-139.85
-139.89
-139.90
-139.92
-139.93
-139.94
-139.6 -139.8 -140.0
f1 (ppm)
-160.94
-160.95
-160.97
-161.01
-161.02
-161.05
-161.06
-161.06
-161.10
-161.12
-161.13
-161.14
-160.8 -161.1
f1 (ppm)
30 20 10 0 -10 -20 -30 -40 -50 -60 -70 -80 -90 -110 -130 -150 -170 -190
f1 (ppm)

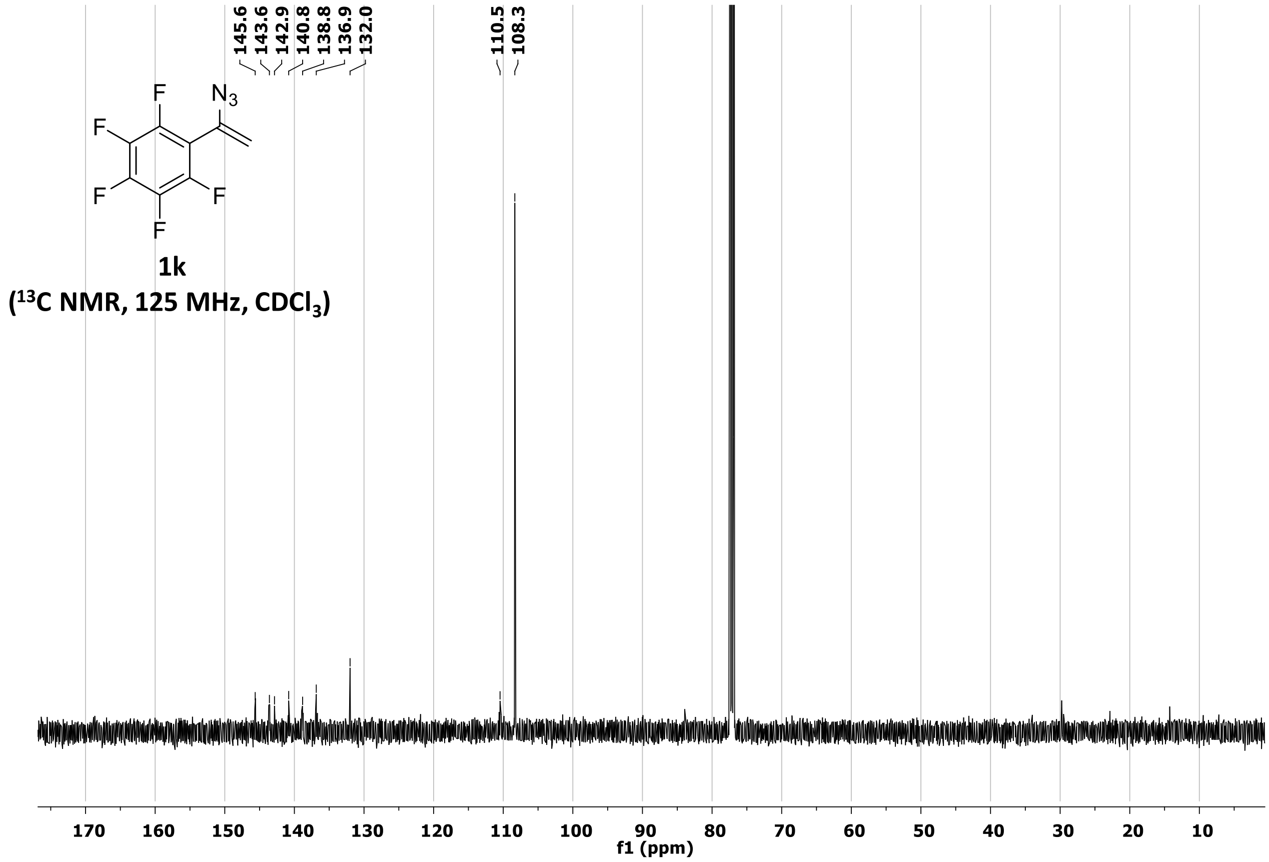


**1k**

**($^{13}C$ NMR, 125 MHz, $CDCl_3$)**

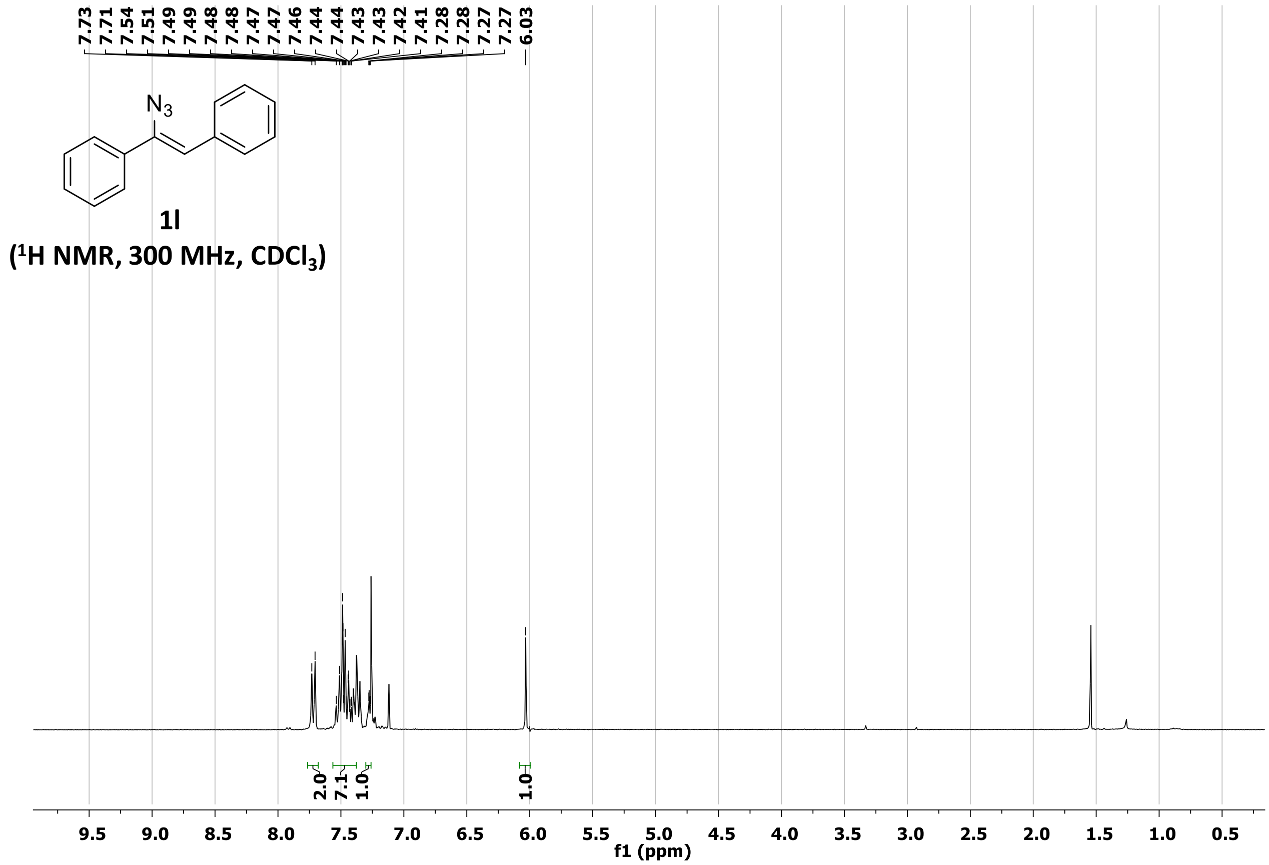


**1l**

**($^{1}H$ NMR, 300 MHz, $CDCl_3$)**

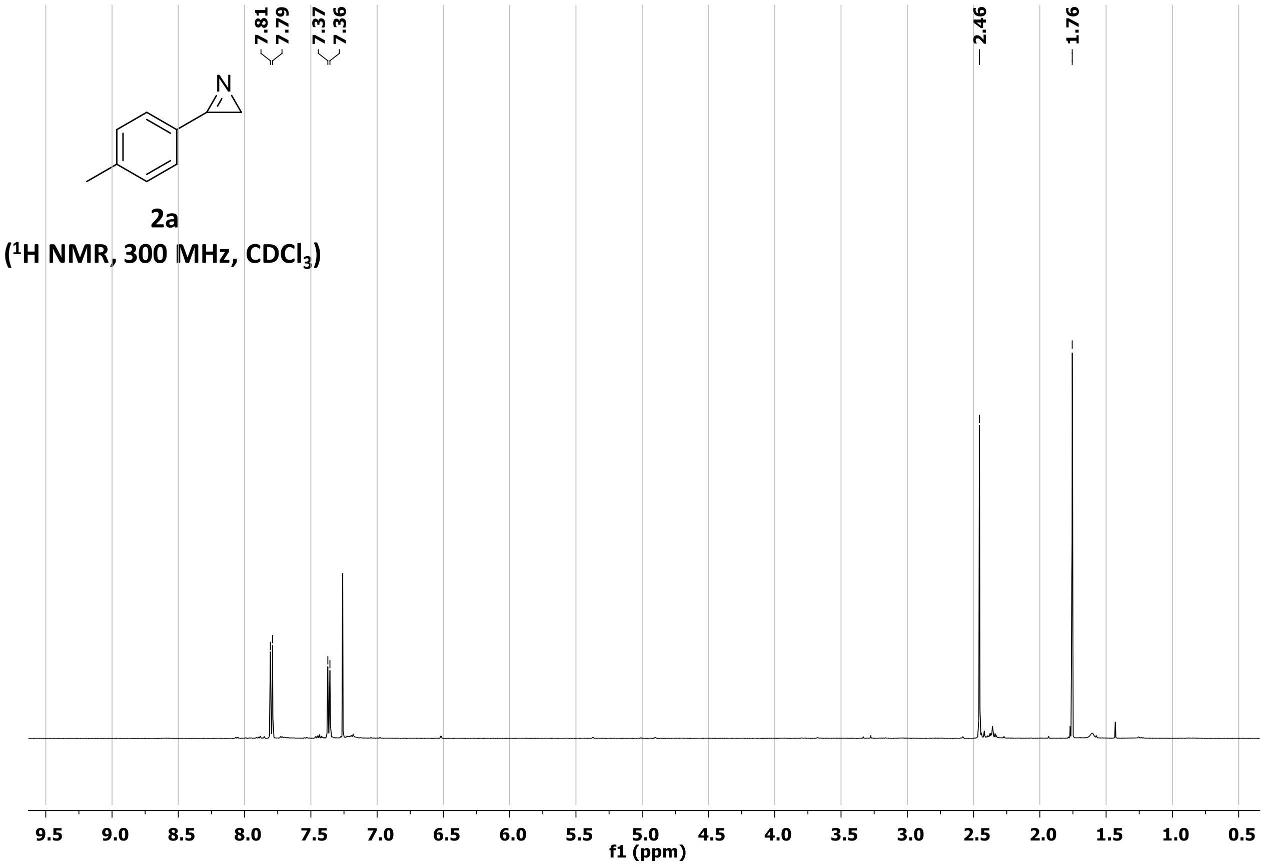


**2a**
($^1$H NMR, 300 MHz, $CDCl_3$)

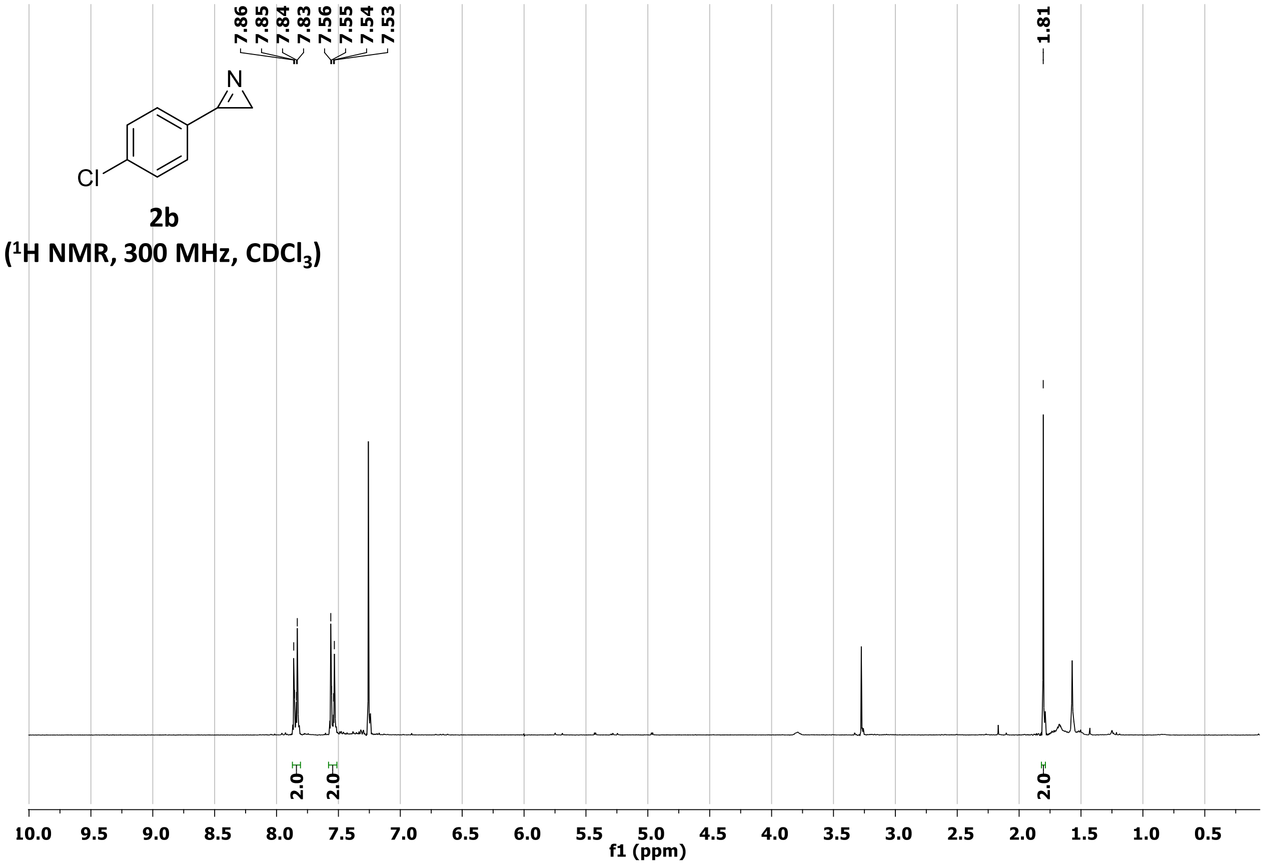


**2b**
($^1$H NMR, 300 MHz, $CDCl_3$)

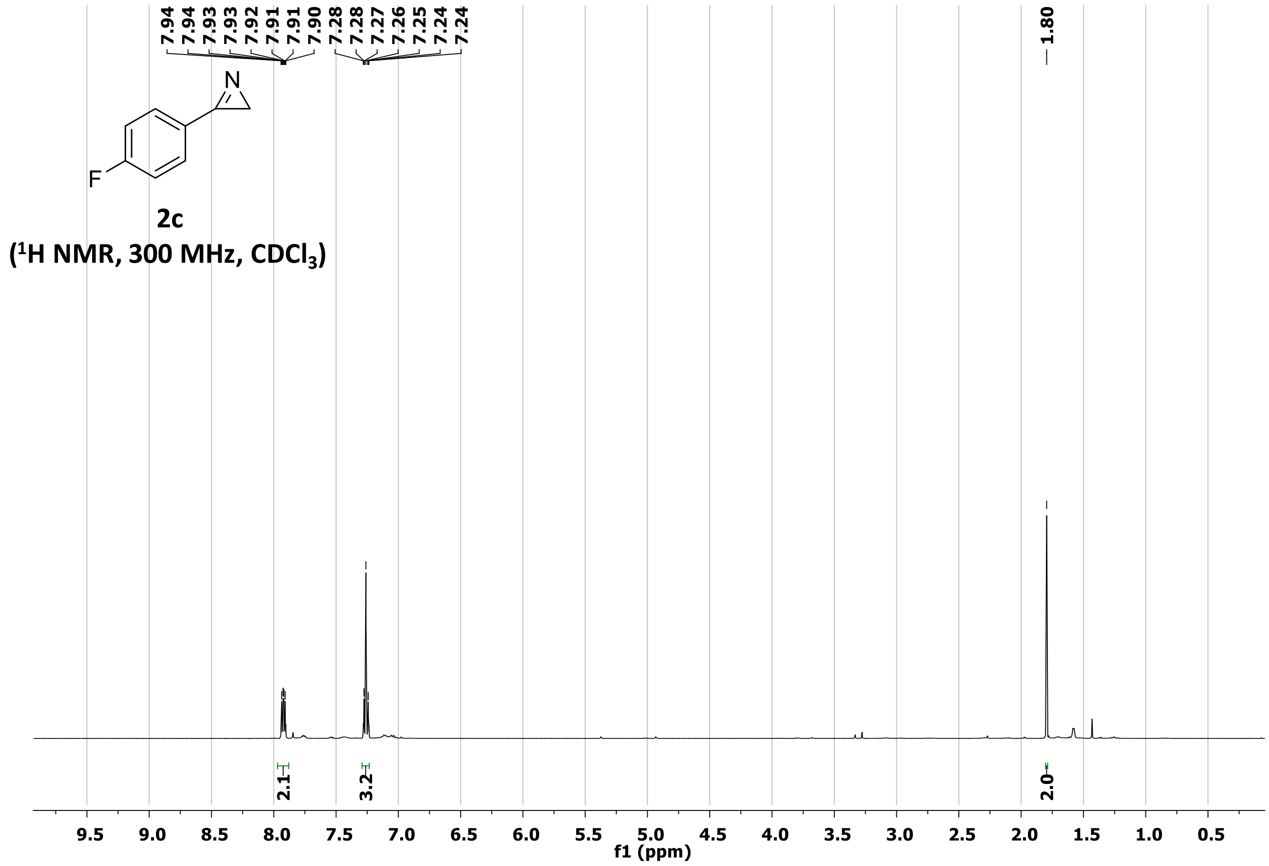
7.94
7.94
7.93
7.93
7.92
7.91
7.91
7.90
7.28
7.28
7.27
7.26
7.25
7.24
7.24
1.80
N
F
2c
(¹H NMR, 300 MHz, CDCl₃)
2.1
3.2
2.0
9.5 9.0 8.5 8.0 7.5 7.0 6.5 6.0 5.5 5.0 4.5 4.0 3.5 3.0 2.5 2.0 1.5 1.0 0.5
f1 (ppm)

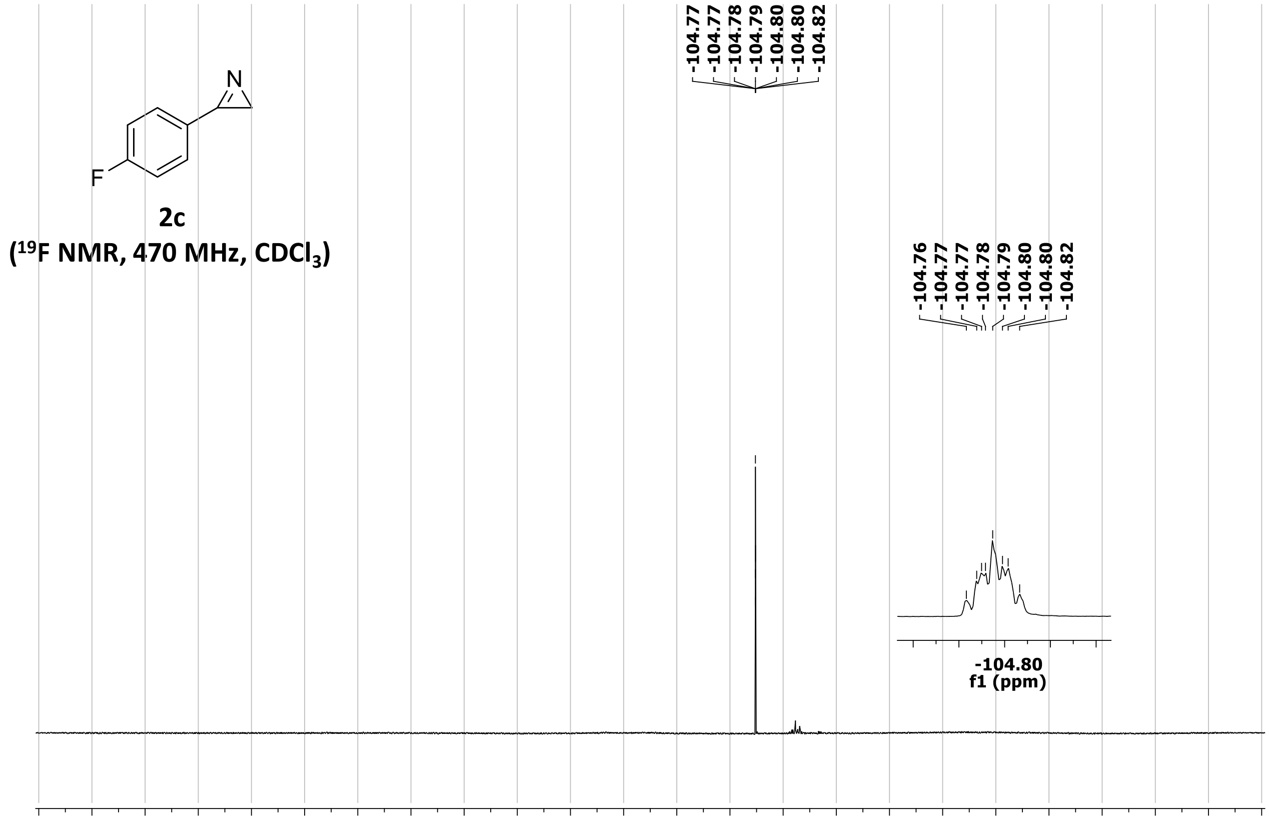
-104.77
-104.77
-104.78
-104.79
-104.80
-104.80
-104.82
N
F
2c
(¹⁹F NMR, 470 MHz, CDCl₃)
-104.76
-104.77
-104.77
-104.78
-104.79
-104.80
-104.80
-104.82
-104.80
f1 (ppm)
30 20 10 0 -10 -20 -30 -40 -50 -60 -70 -80 -90 -110 -130 -150 -170 -190
f1 (ppm)

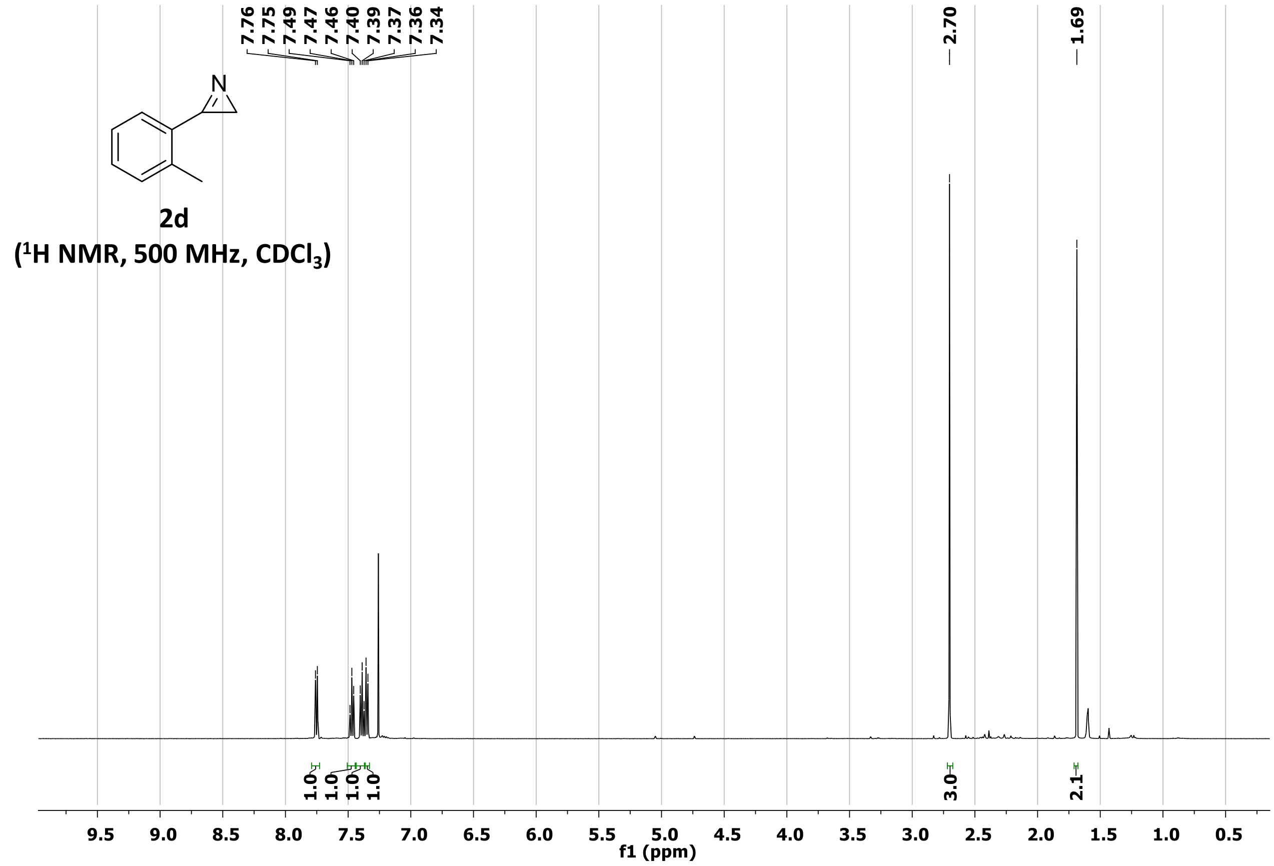
7.76
7.75
7.49
7.47
7.46
7.40
7.39
7.37
7.36
7.34
2.70
1.69
N
2d
(¹H NMR, 500 MHz, CDCl₃)
1.0
1.0
1.0
1.0
3.0
2.1
9.5
9.0
8.5
8.0
7.5
7.0
6.5
6.0
5.5
5.0
4.5
4.0
3.5
3.0
2.5
2.0
1.5
1.0
0.5
f1 (ppm)

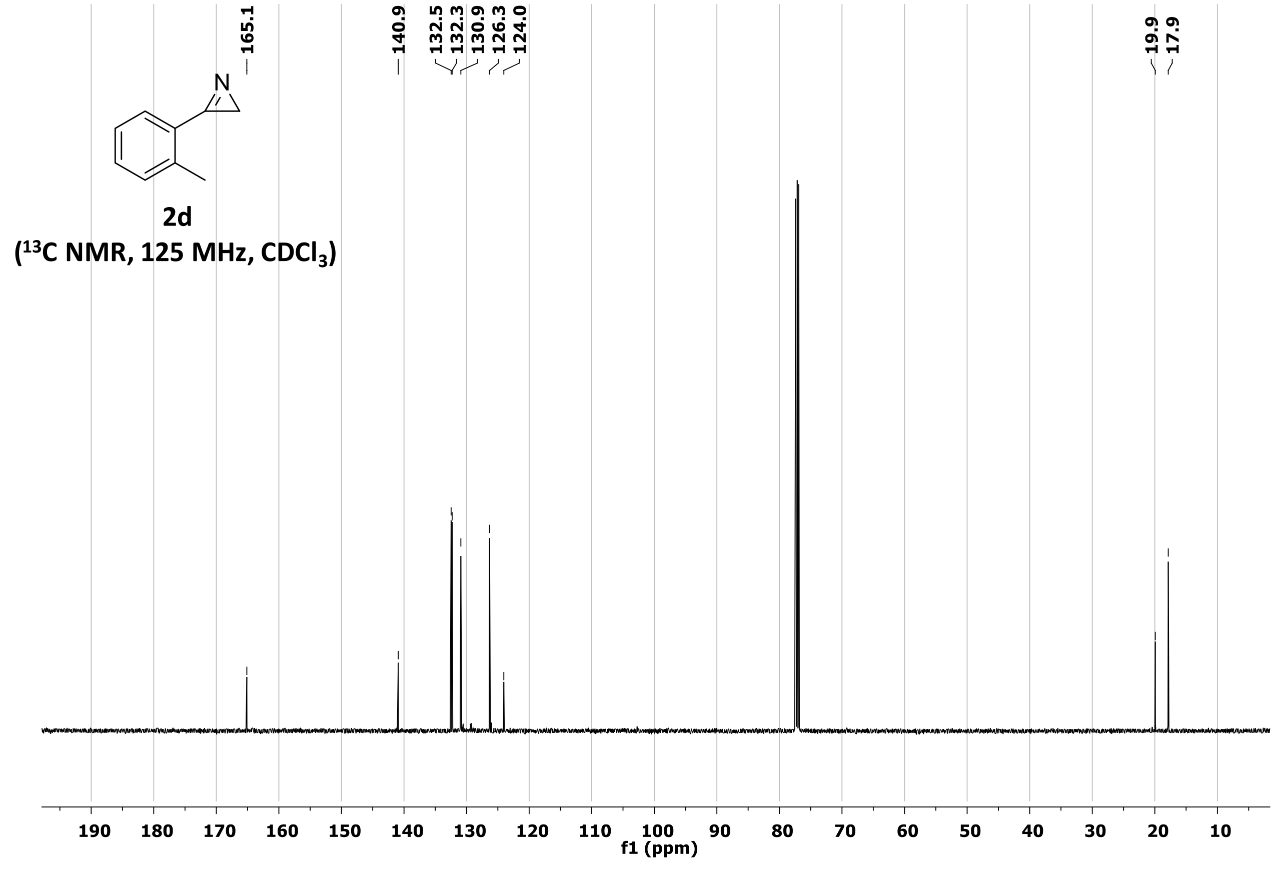
165.1
140.9
132.5
132.3
130.9
126.3
124.0
19.9
17.9
N
2d
(¹³C NMR, 125 MHz, CDCl₃)
190
180
170
160
150
140
130
120
110
100
90
80
70
60
50
40
30
20
10
f1 (ppm)

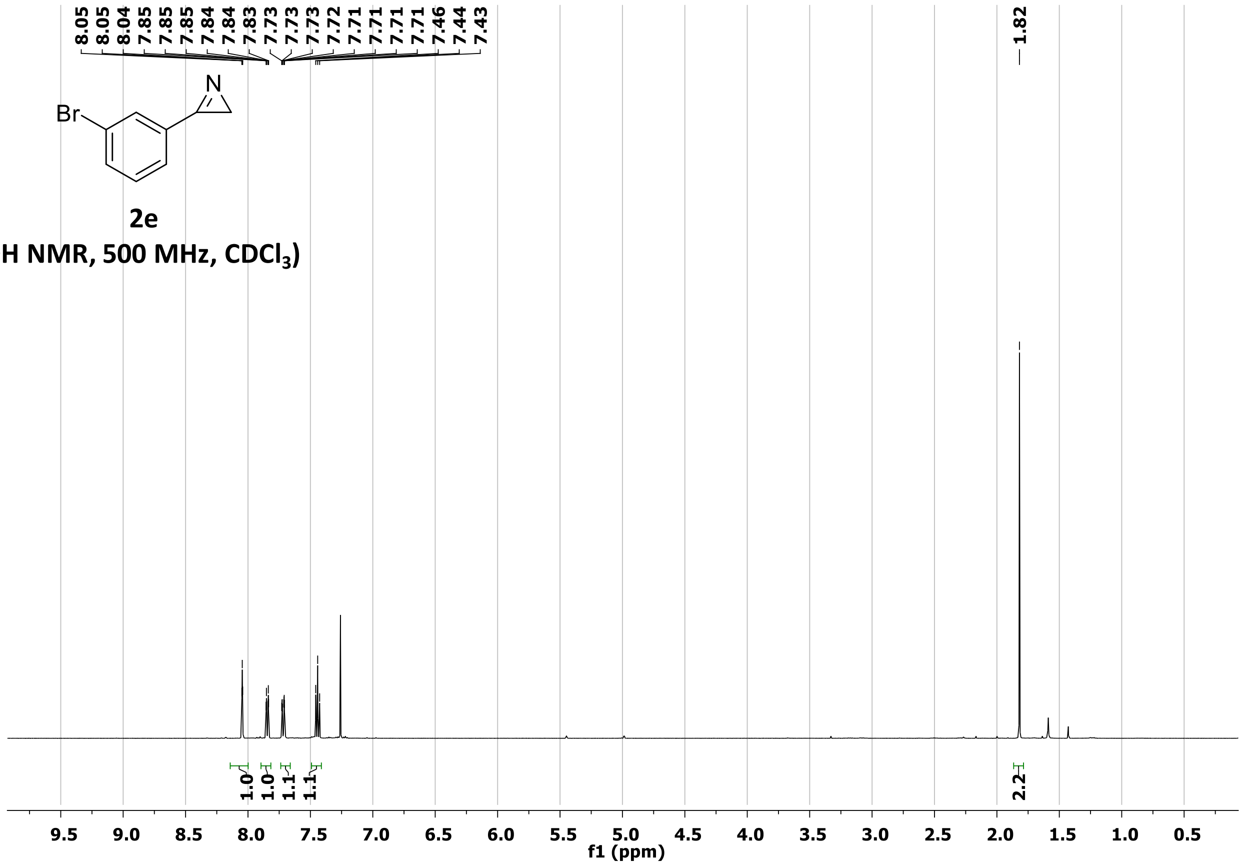


**2e**
**($^{1}$H NMR, 500 MHz, $CDCl_3$)**

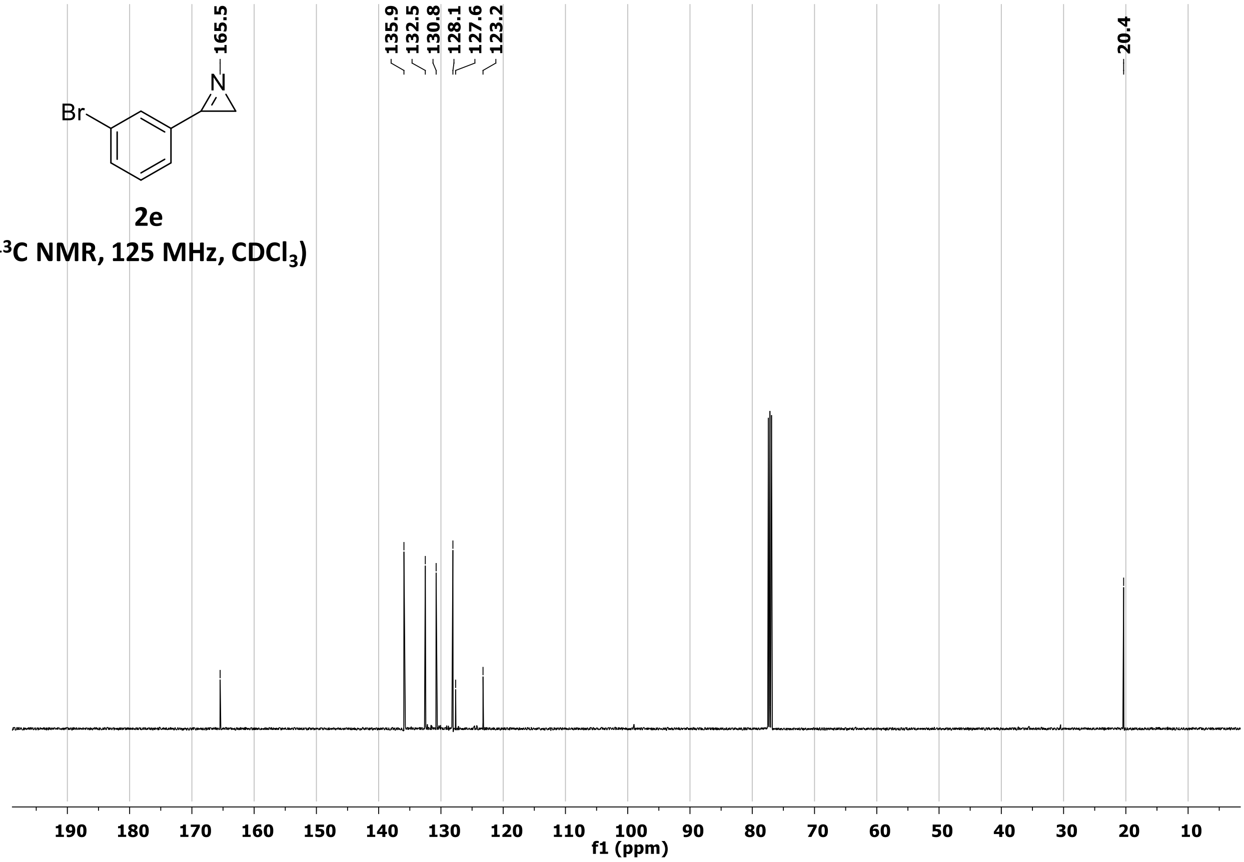


**2e**
**($^{13}$C NMR, 125 MHz, $CDCl_3$)**

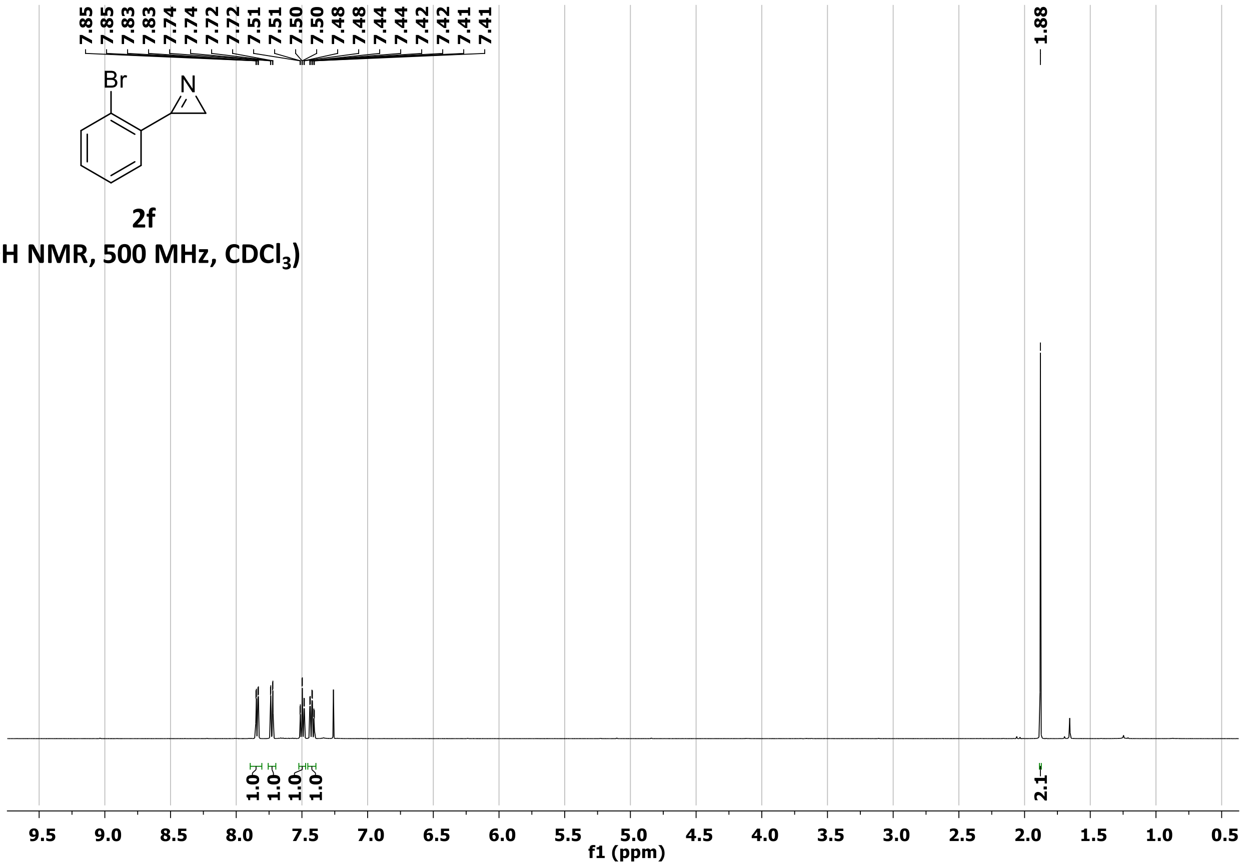


**2f**
($^1$H NMR, 500 MHz, $CDCl_3$)

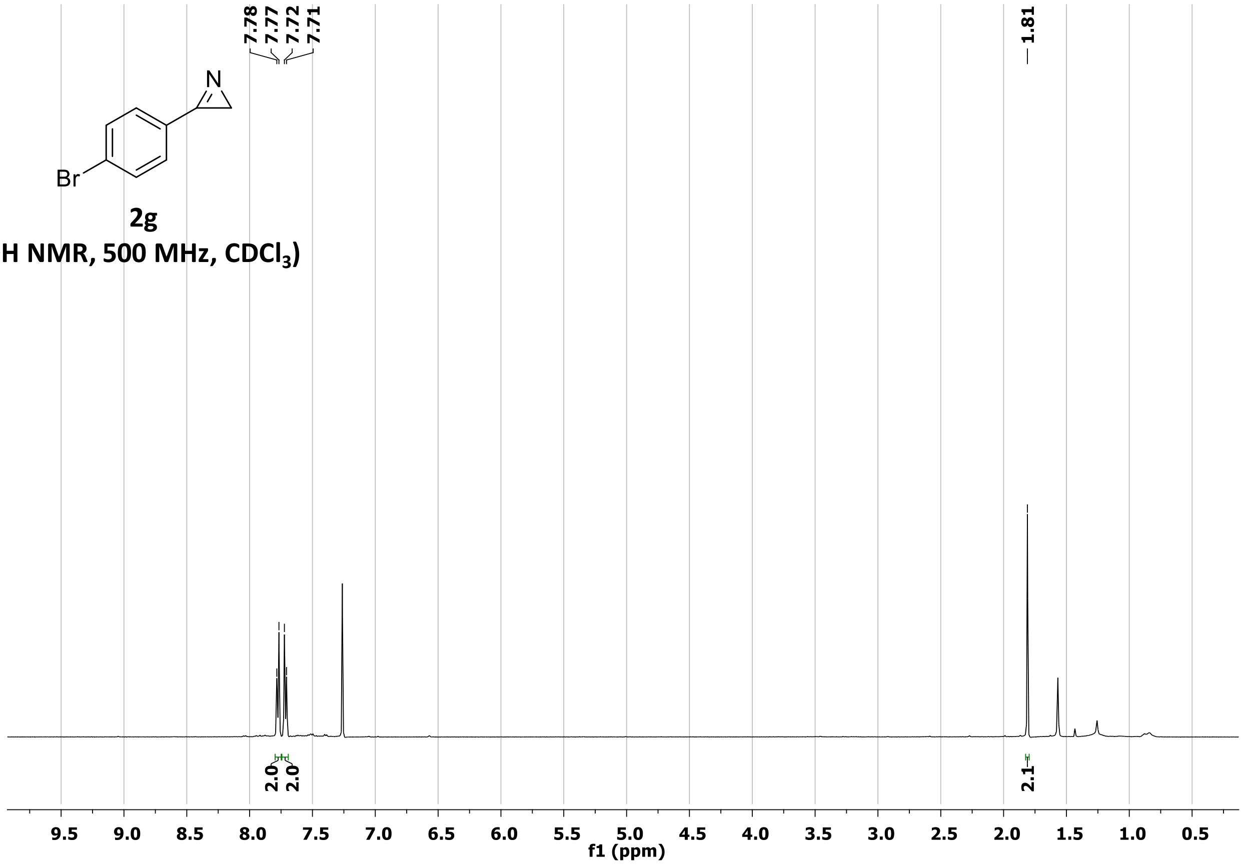


**2g**
($^1$H NMR, 500 MHz, $CDCl_3$)

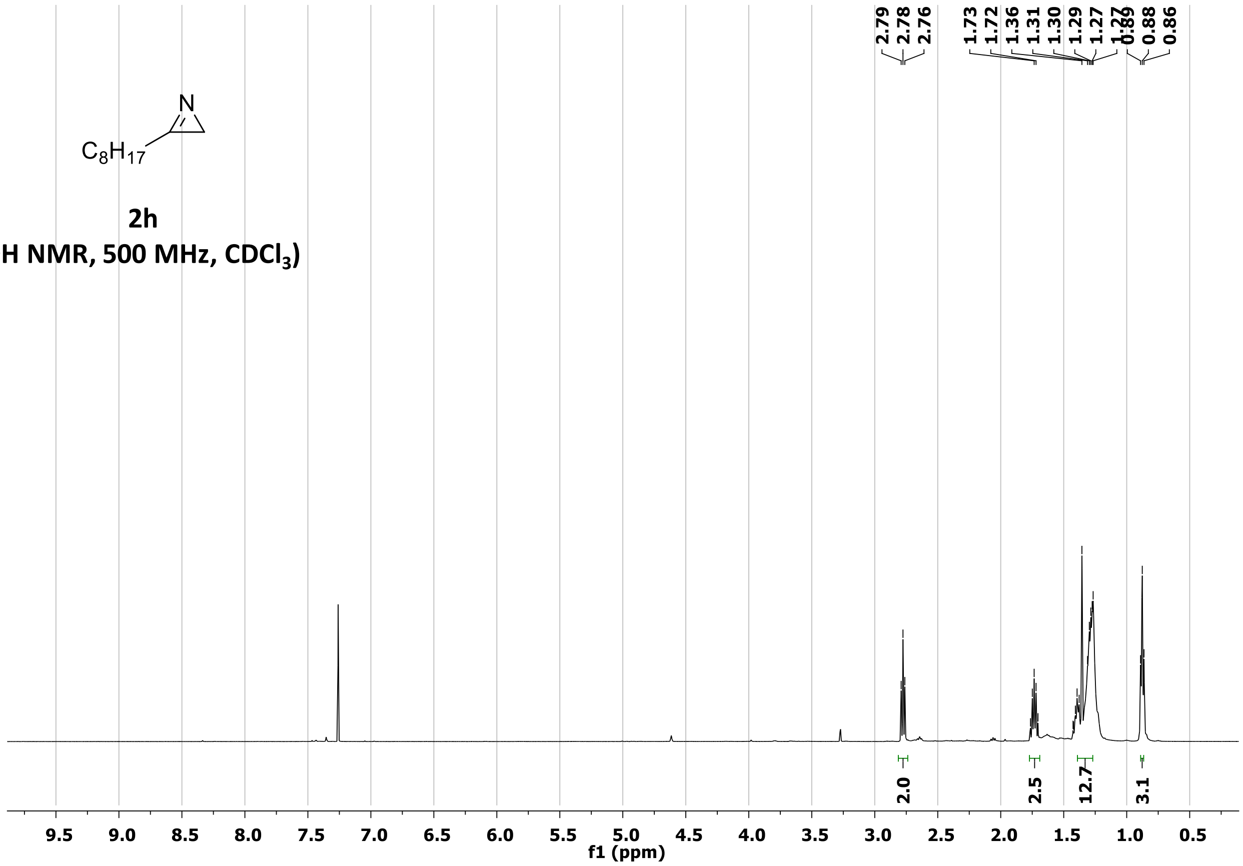


**2h**
**($^1$H NMR, 500 MHz, $CDCl_3$)**

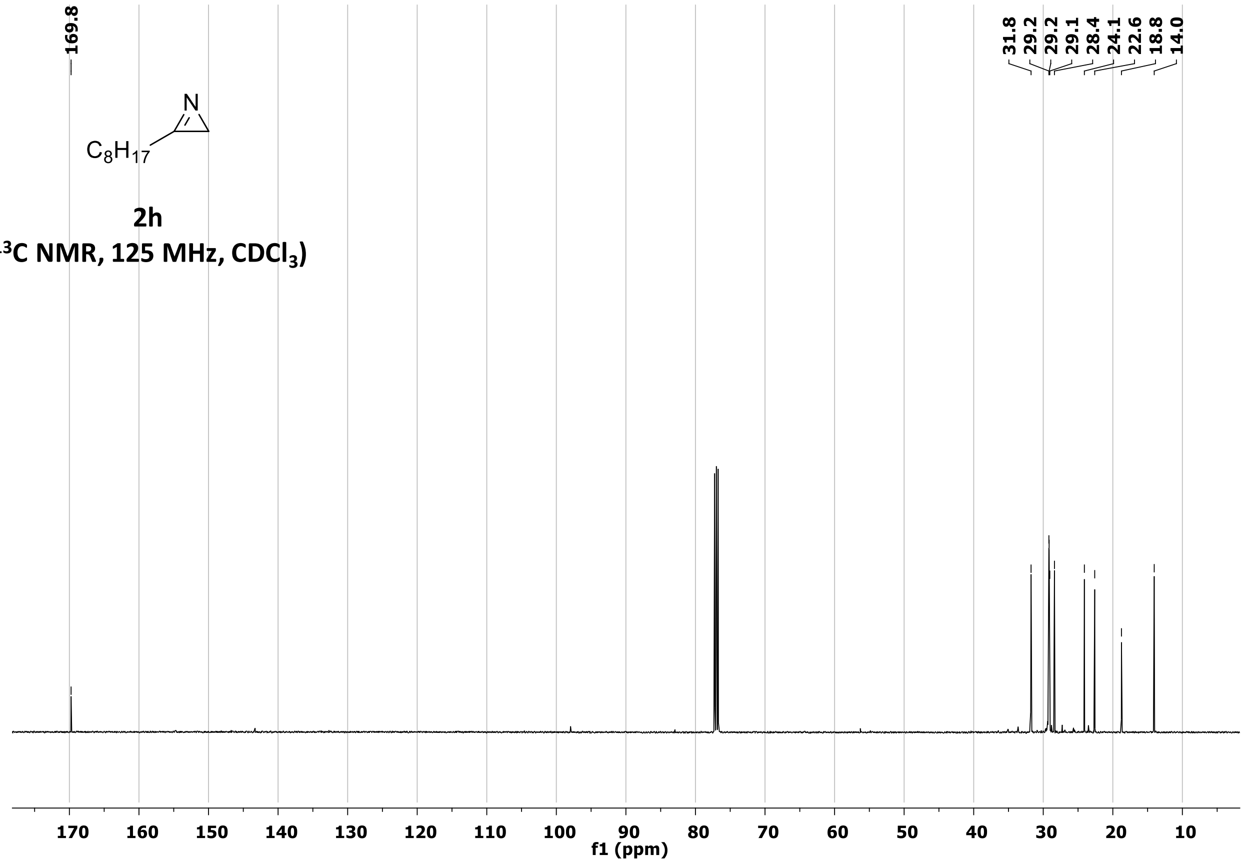


**2h**
**($^{13}$C NMR, 125 MHz, $CDCl_3$)**

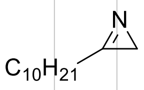


**2i**
**($^1$H NMR, 500 MHz, $CDCl_3$)**

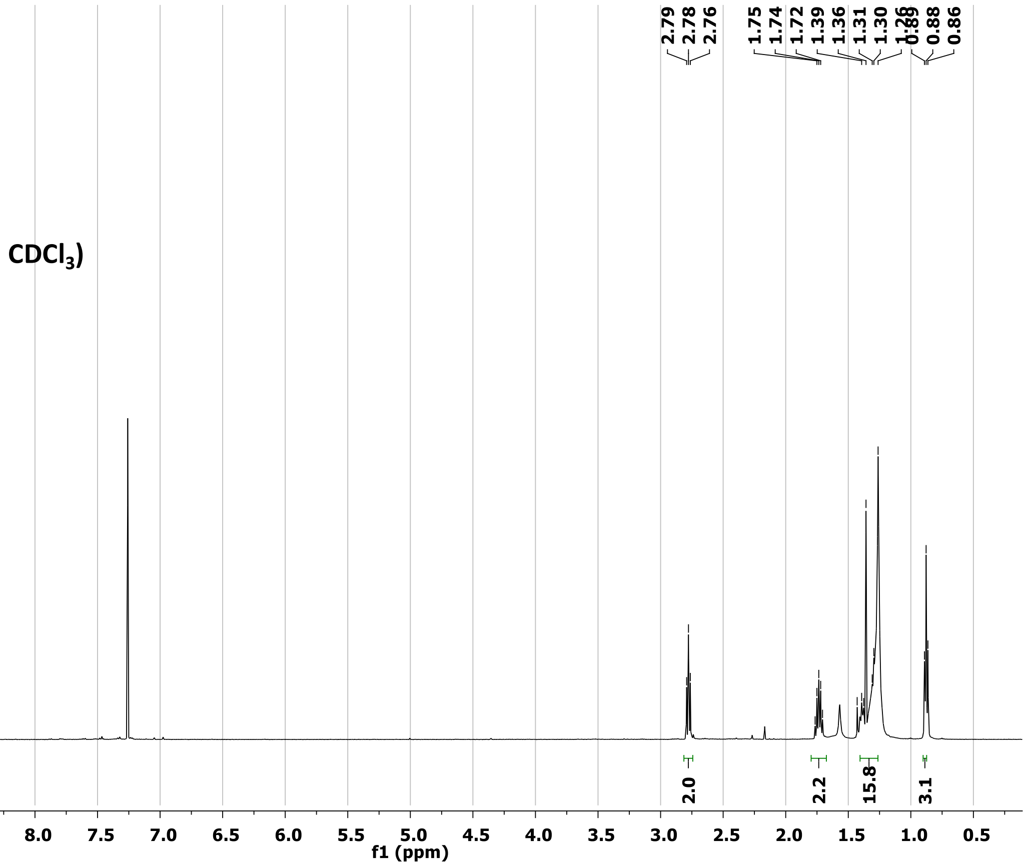


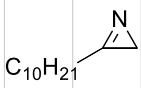


**2i**
**($^{13}$C NMR, 125 MHz, $CDCl_3$)**

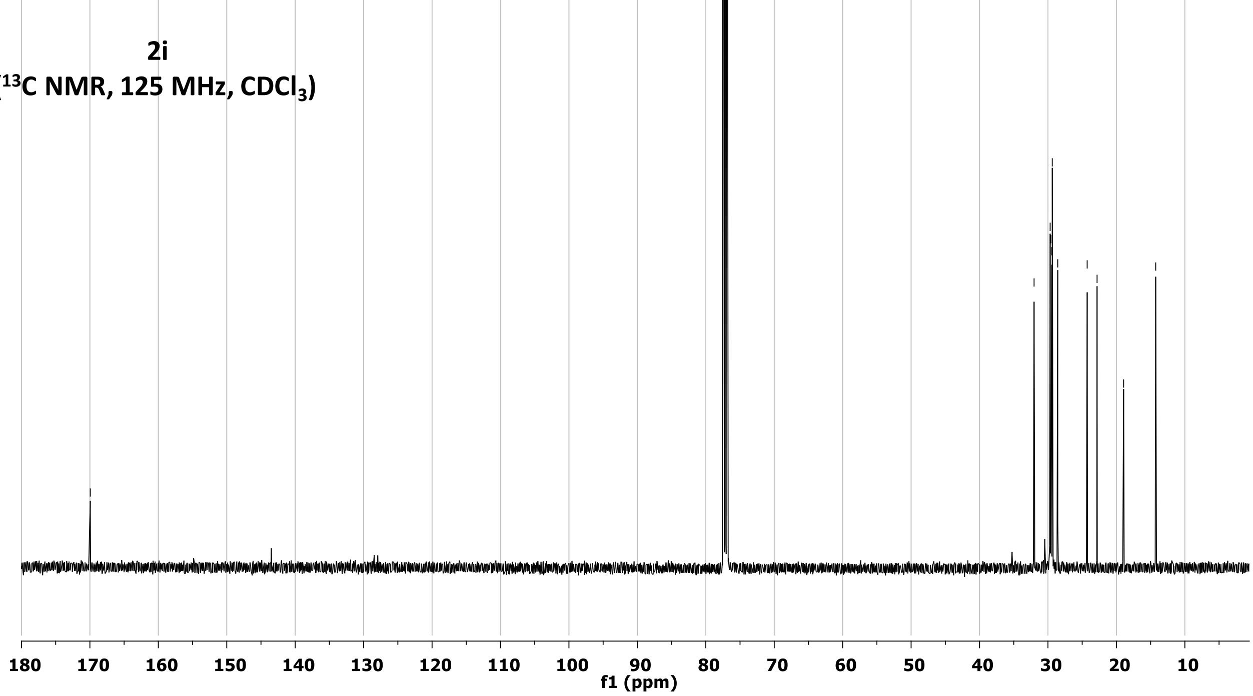

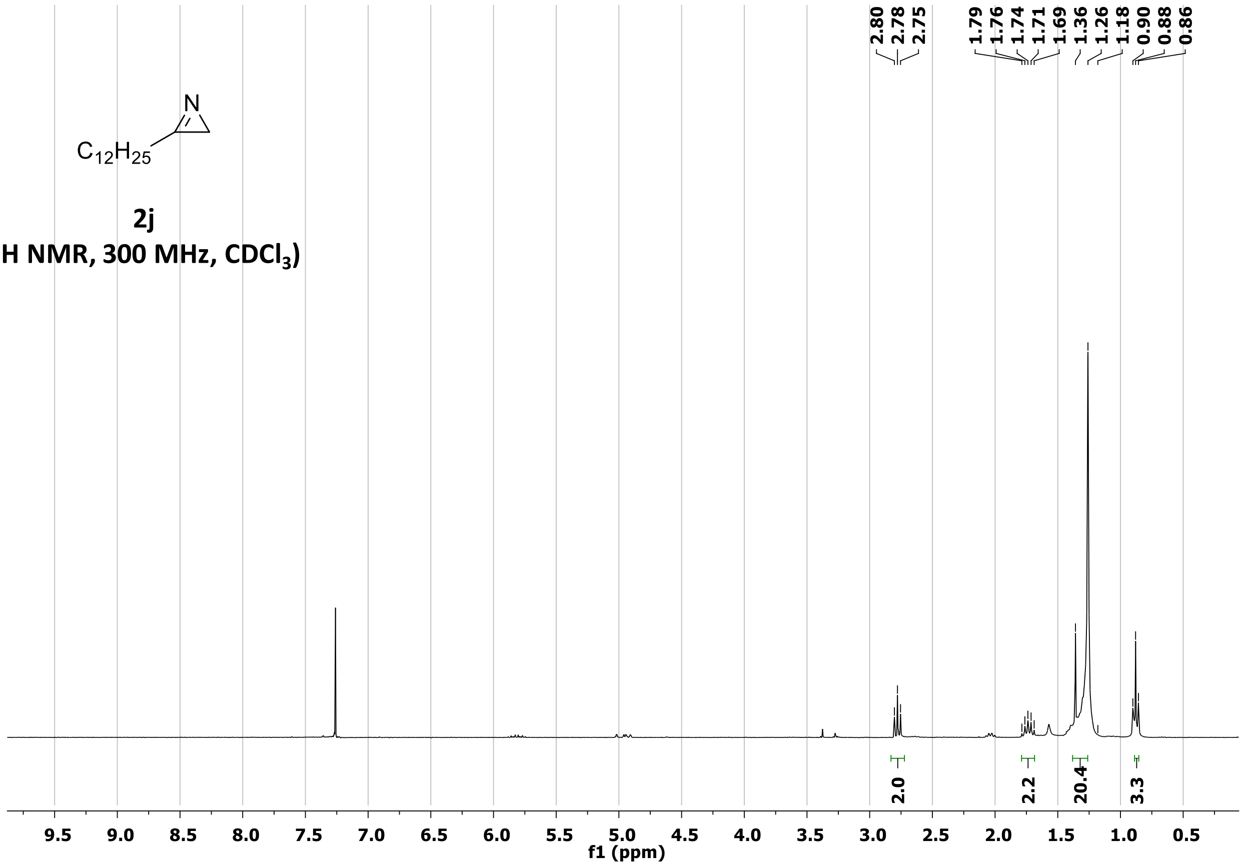


**2j**
**($^{1}H$ NMR, 300 MHz, $CDCl_3$)**

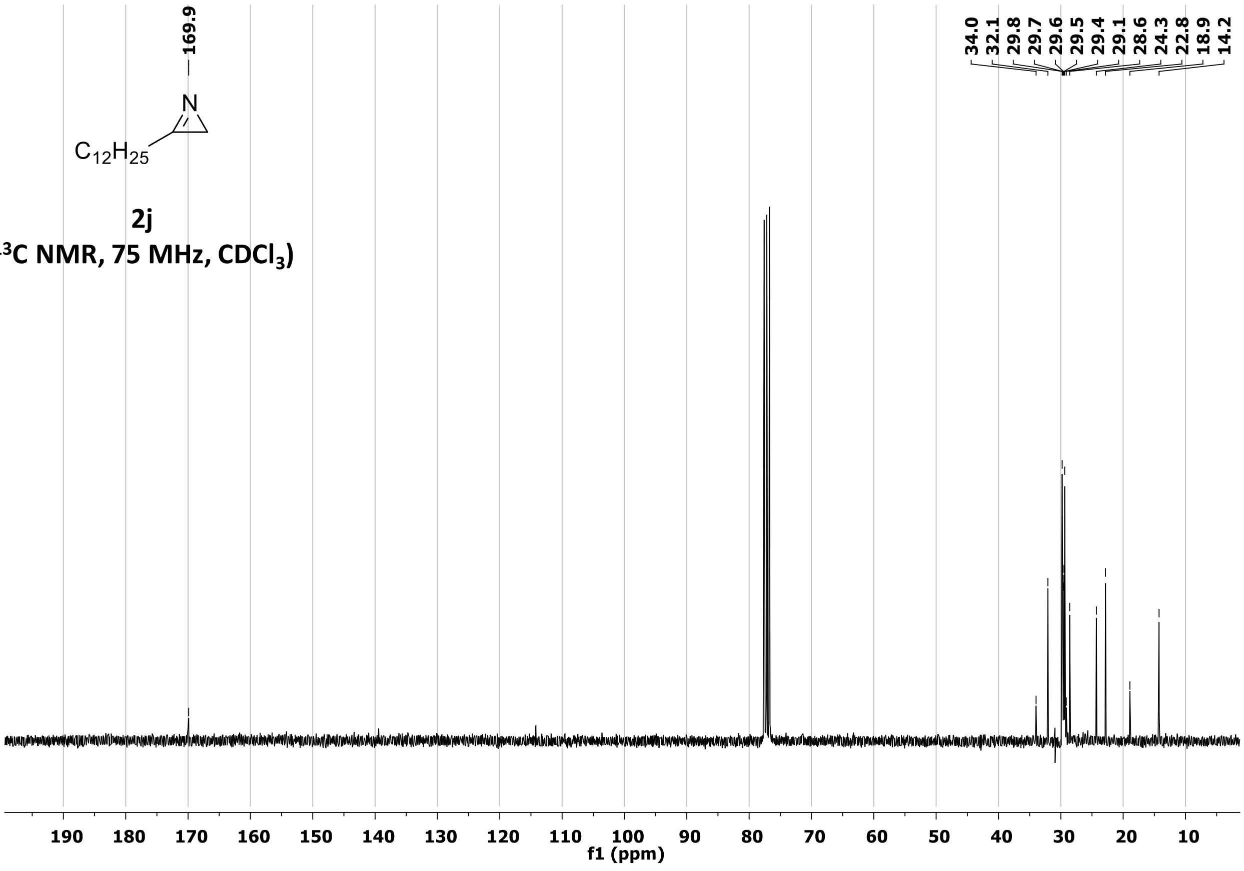


**2j**
**($^{13}C$ NMR, 75 MHz, $CDCl_3$)**

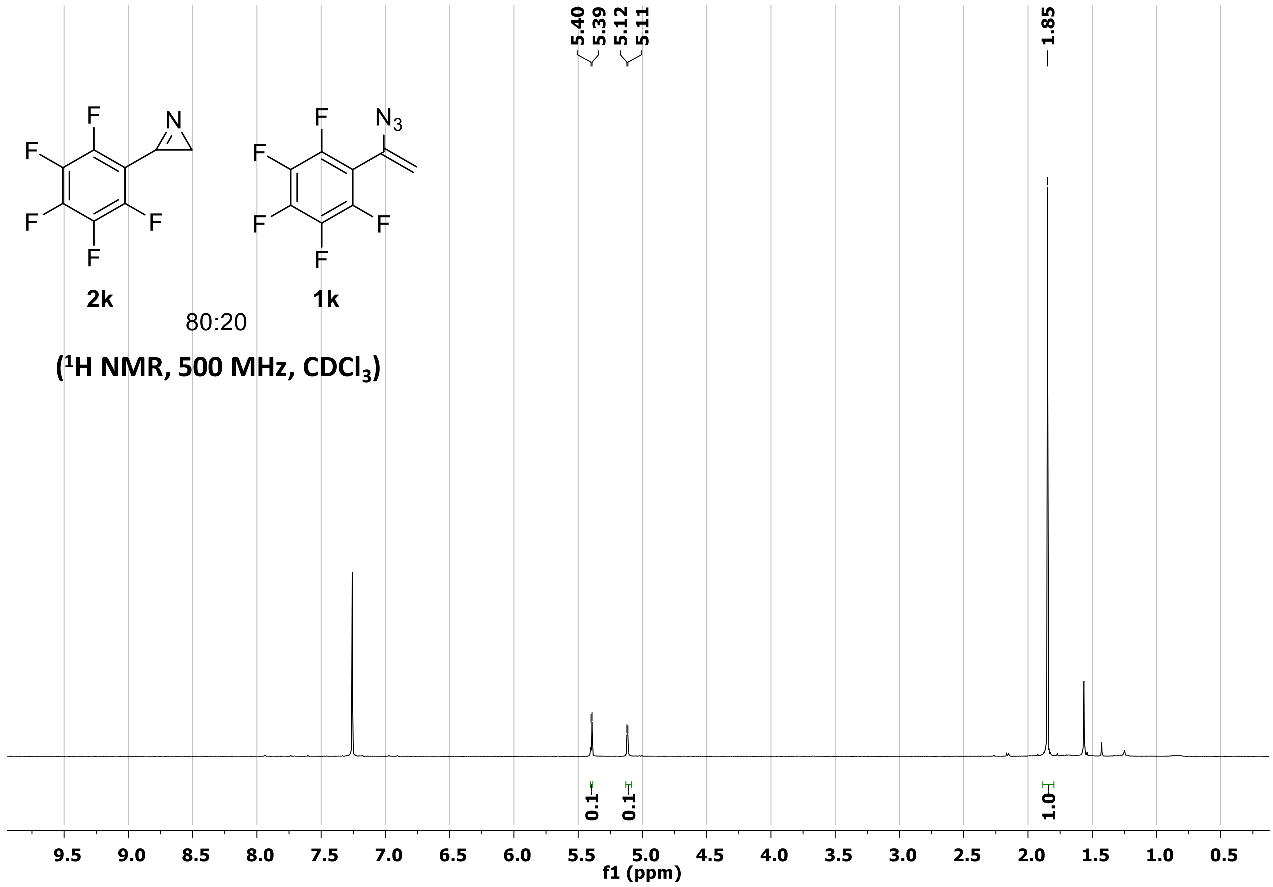
5.40
5.39
5.12
5.11
1.85
F
N
N3
2k
80:20
1k
(1H NMR, 500 MHz, CDCl3)
0.1
0.1
1.0
9.5 9.0 8.5 8.0 7.5 7.0 6.5 6.0 5.5 5.0 4.5 4.0 3.5 3.0 2.5 2.0 1.5 1.0 0.5
f1 (ppm)

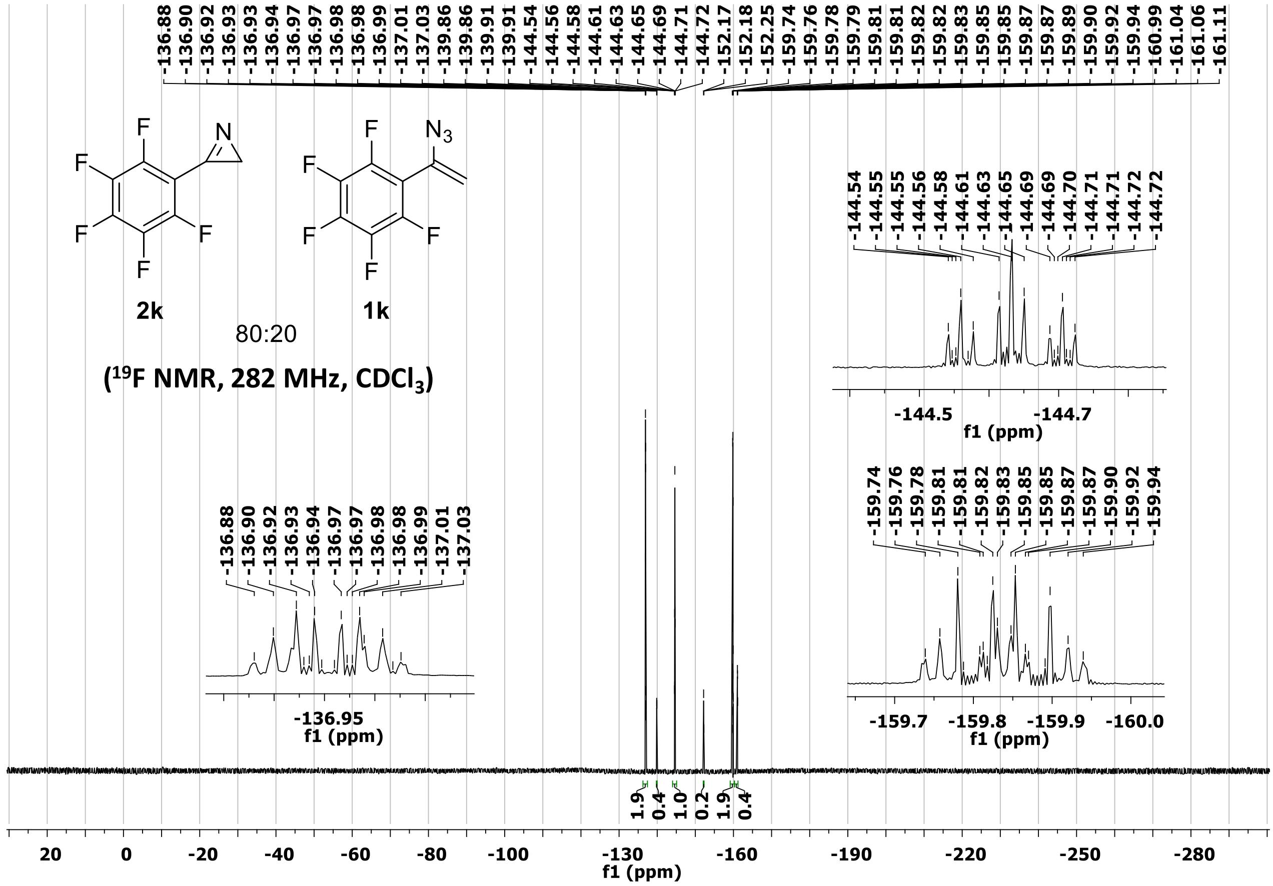
-136.88 -136.90 -136.92 -136.93 -136.94 -136.97 -136.97 -136.98 -136.98 -136.99 -137.01 -137.03 -139.86 -139.86 -139.91 -139.91 -144.54 -144.56 -144.58 -144.61 -144.63 -144.65 -144.69 -144.71 -144.72 -152.17 -152.18 -152.25 -159.74 -159.76 -159.78 -159.79 -159.81 -159.81 -159.82 -159.82 -159.83 -159.85 -159.85 -159.87 -159.87 -159.89 -159.90 -159.92 -159.94 -160.99 -161.04 -161.06 -161.11
F
N
N3
2k
80:20
1k
(19F NMR, 282 MHz, CDCl3)
-144.54 -144.55 -144.55 -144.56 -144.58 -144.61 -144.63 -144.65 -144.69 -144.69 -144.70 -144.71 -144.71 -144.72 -144.72
-144.5 -144.7
f1 (ppm)
-136.88 -136.90 -136.92 -136.93 -136.94 -136.97 -136.97 -136.98 -136.98 -136.99 -137.01 -137.03
-136.95
f1 (ppm)
-159.74 -159.76 -159.78 -159.81 -159.81 -159.82 -159.83 -159.85 -159.85 -159.87 -159.87 -159.90 -159.92 -159.94
-159.7 -159.8 -159.9 -160.0
f1 (ppm)
1.9
0.4
1.0
0.2
1.9
0.4
20 0 -20 -40 -60 -80 -100 -130 -160 -190 -220 -250 -280
f1 (ppm)

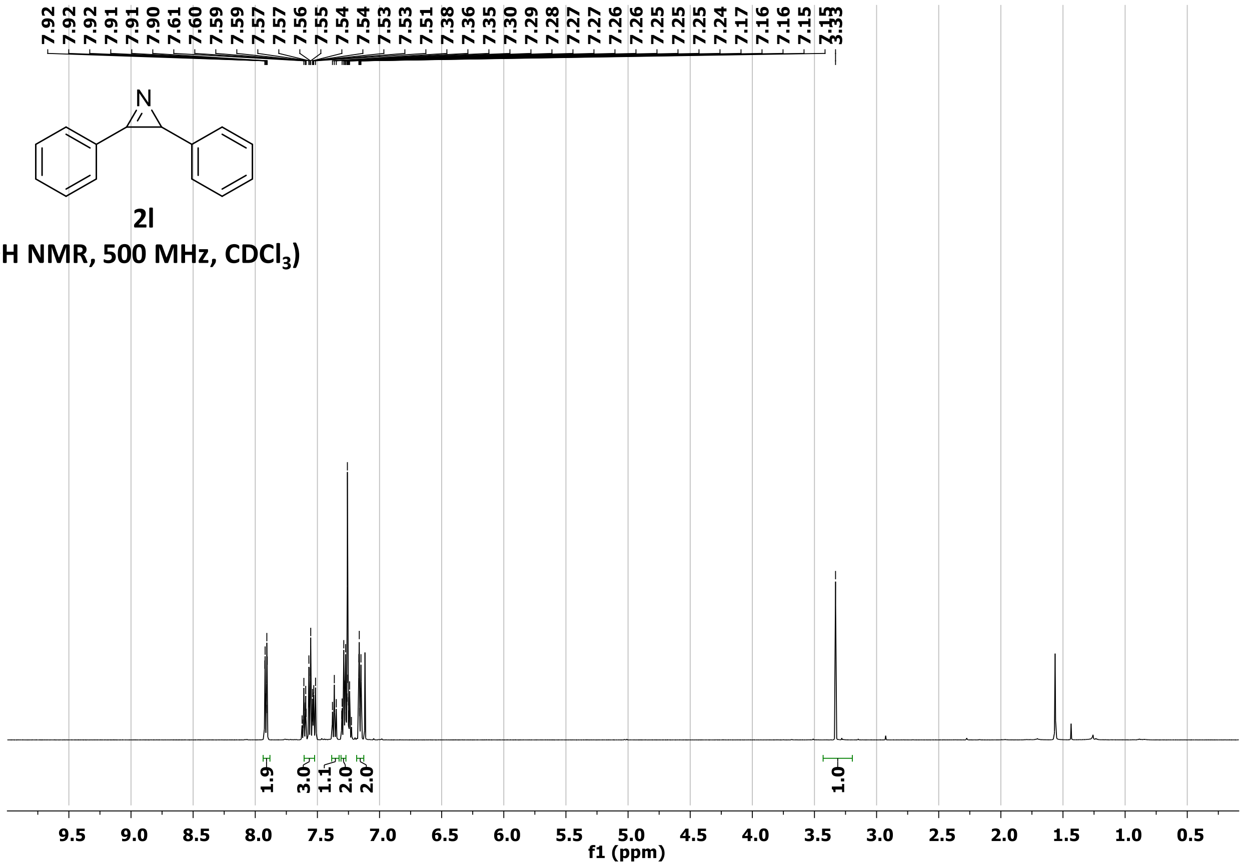


**2l**
($^1$H NMR, 500 MHz, $CDCl_3$)

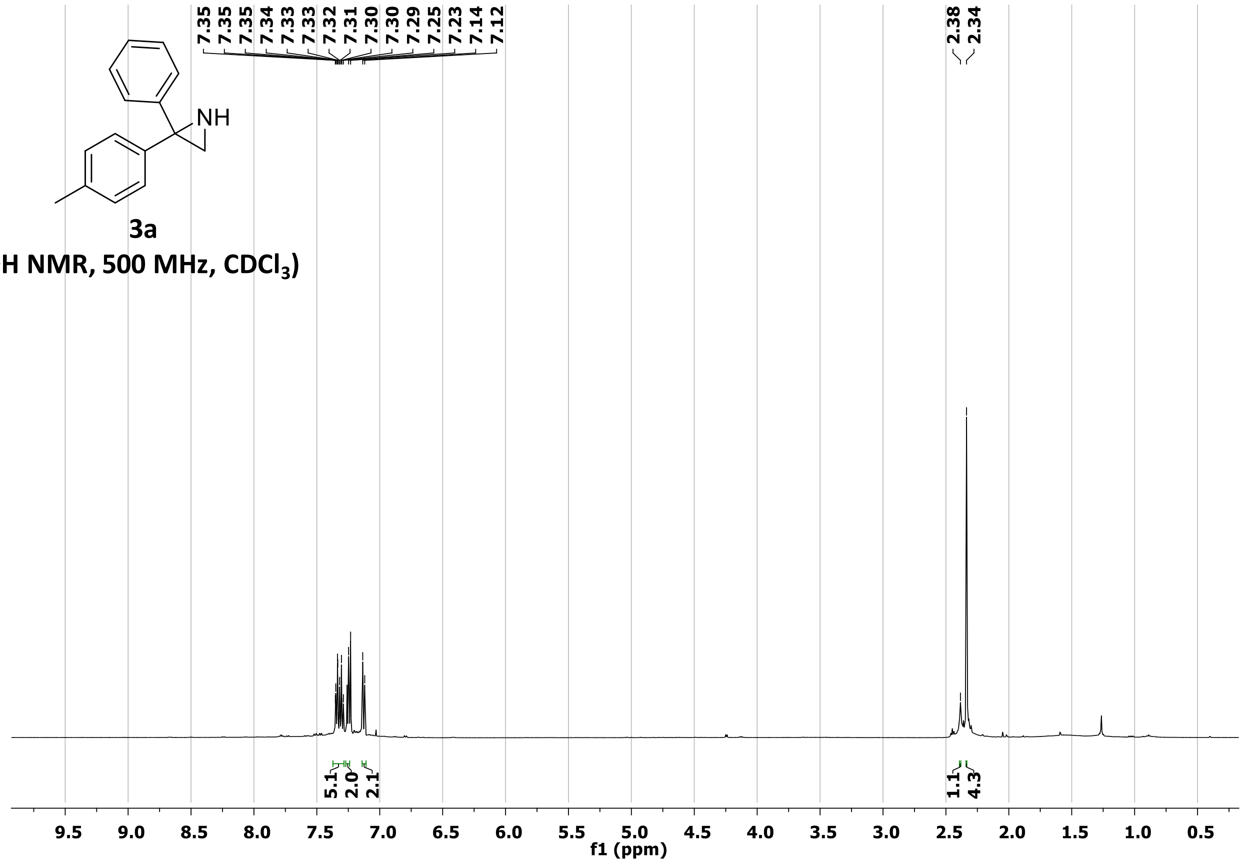


**3a**
($^1$H NMR, 500 MHz, $CDCl_3$)

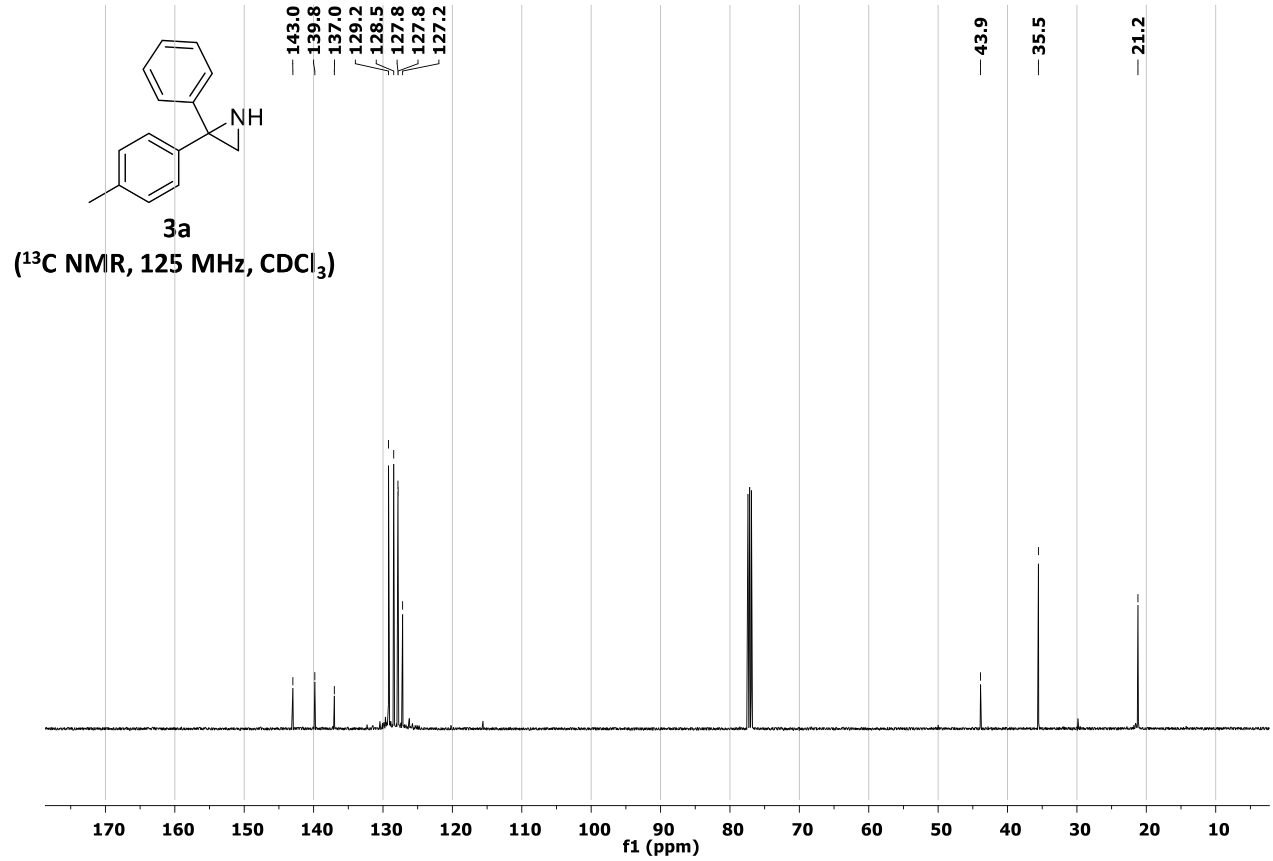


**3a**

($^{13}$C NMR, 125 MHz, $CDCl_3$)

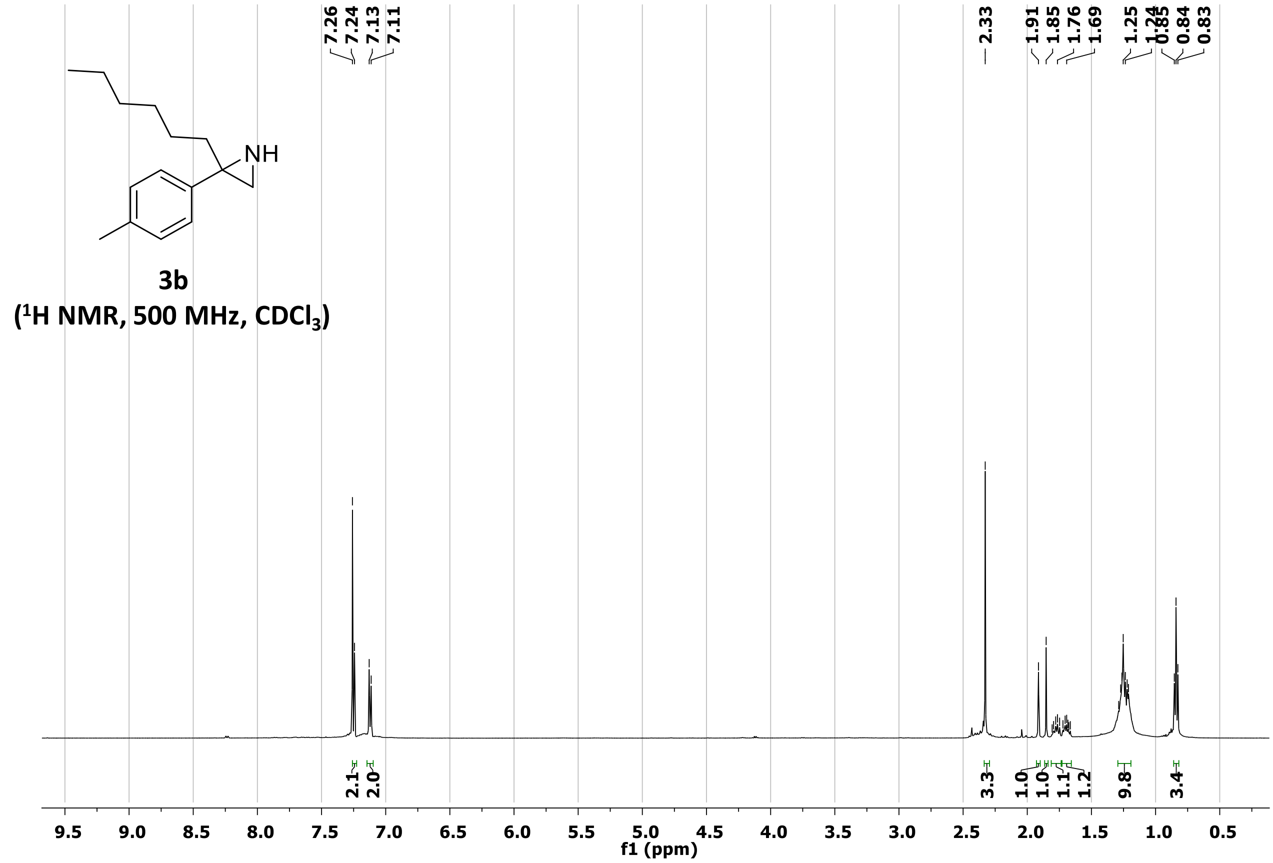


**3b**

($^{1}$H NMR, 500 MHz, $CDCl_3$)

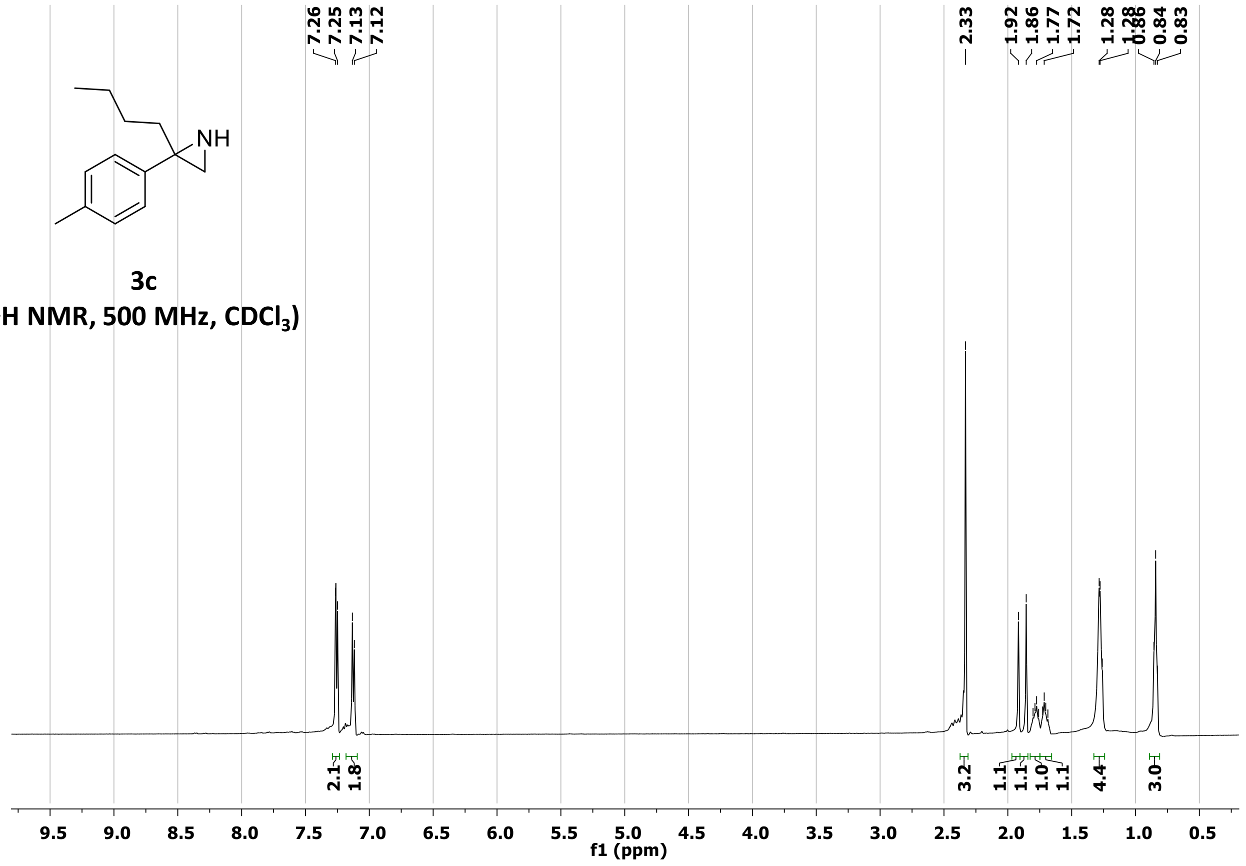


**3c**
**([1]H NMR, 500 MHz, $CDCl_3$)**

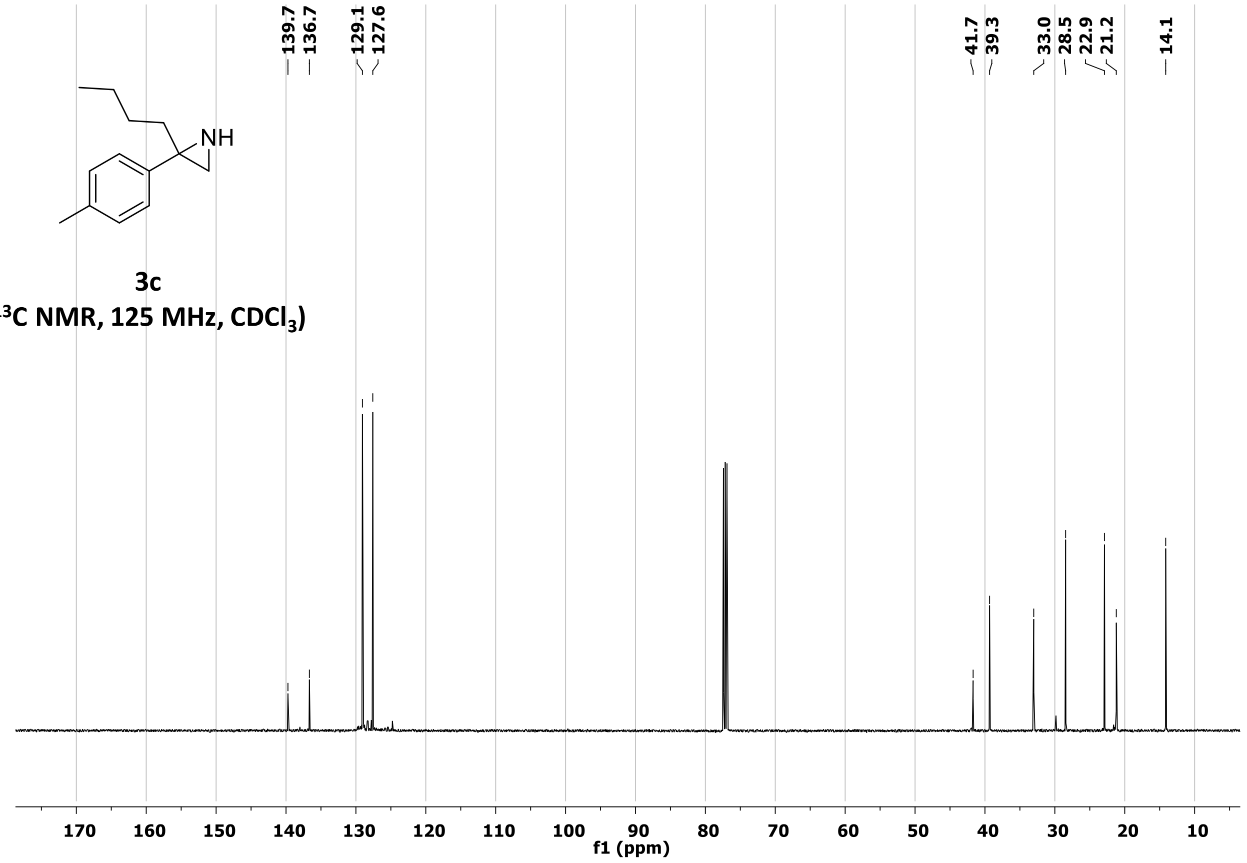


**3c**
**([13]C NMR, 125 MHz, $CDCl_3$)**

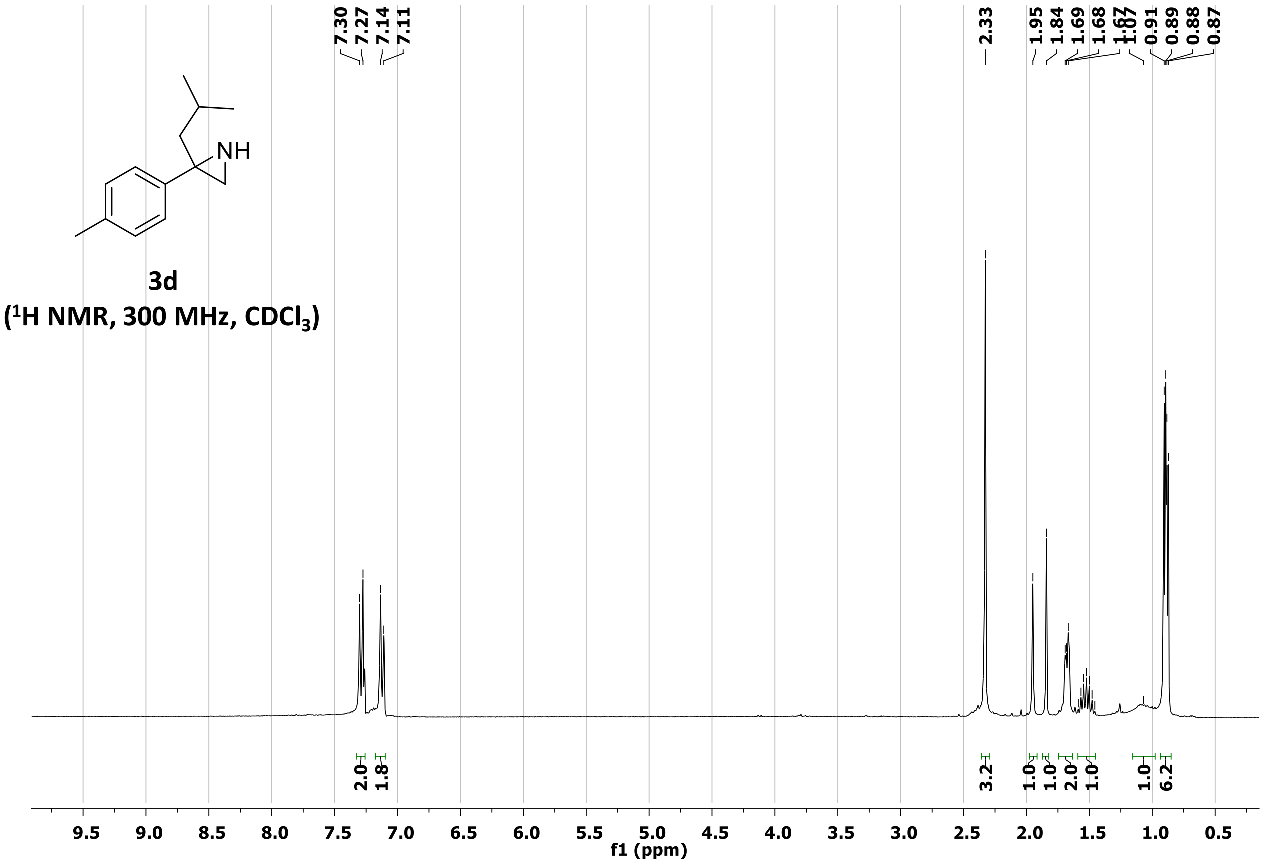
NH
3d
(¹H NMR, 300 MHz, CDCl₃)
7.30
7.27
7.14
7.11
2.33
1.95
1.84
1.69
1.68
1.67
1.07
0.91
0.89
0.88
0.87
2.0
1.8
3.2
1.0
1.0
2.0
1.0
1.0
6.2
9.5
9.0
8.5
8.0
7.5
7.0
6.5
6.0
5.5
5.0
4.5
4.0
3.5
3.0
2.5
2.0
1.5
1.0
0.5
f1 (ppm)

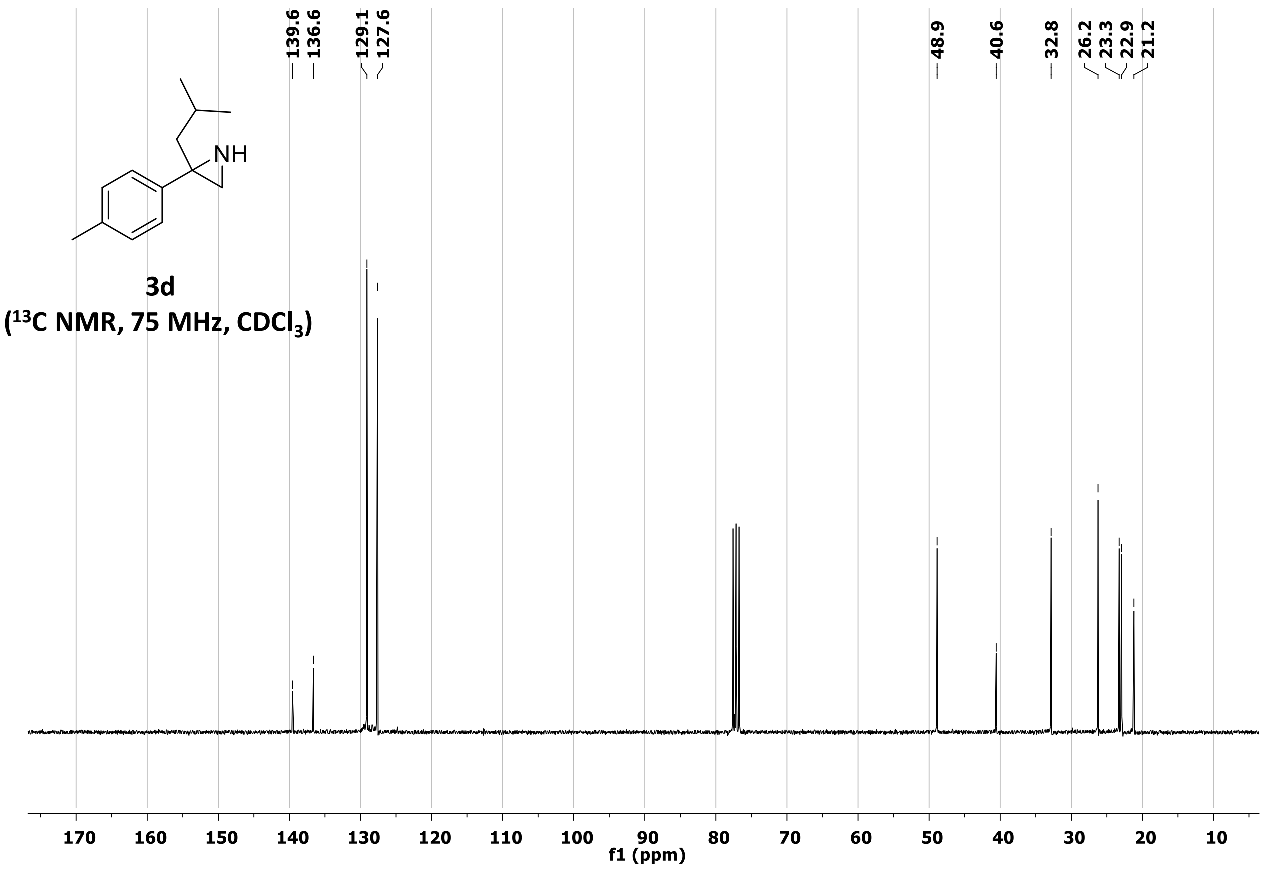
NH
3d
(¹³C NMR, 75 MHz, CDCl₃)
139.6
136.6
129.1
127.6
48.9
40.6
32.8
26.2
23.3
22.9
21.2
170
160
150
140
130
120
110
100
90
80
70
60
50
40
30
20
10
f1 (ppm)

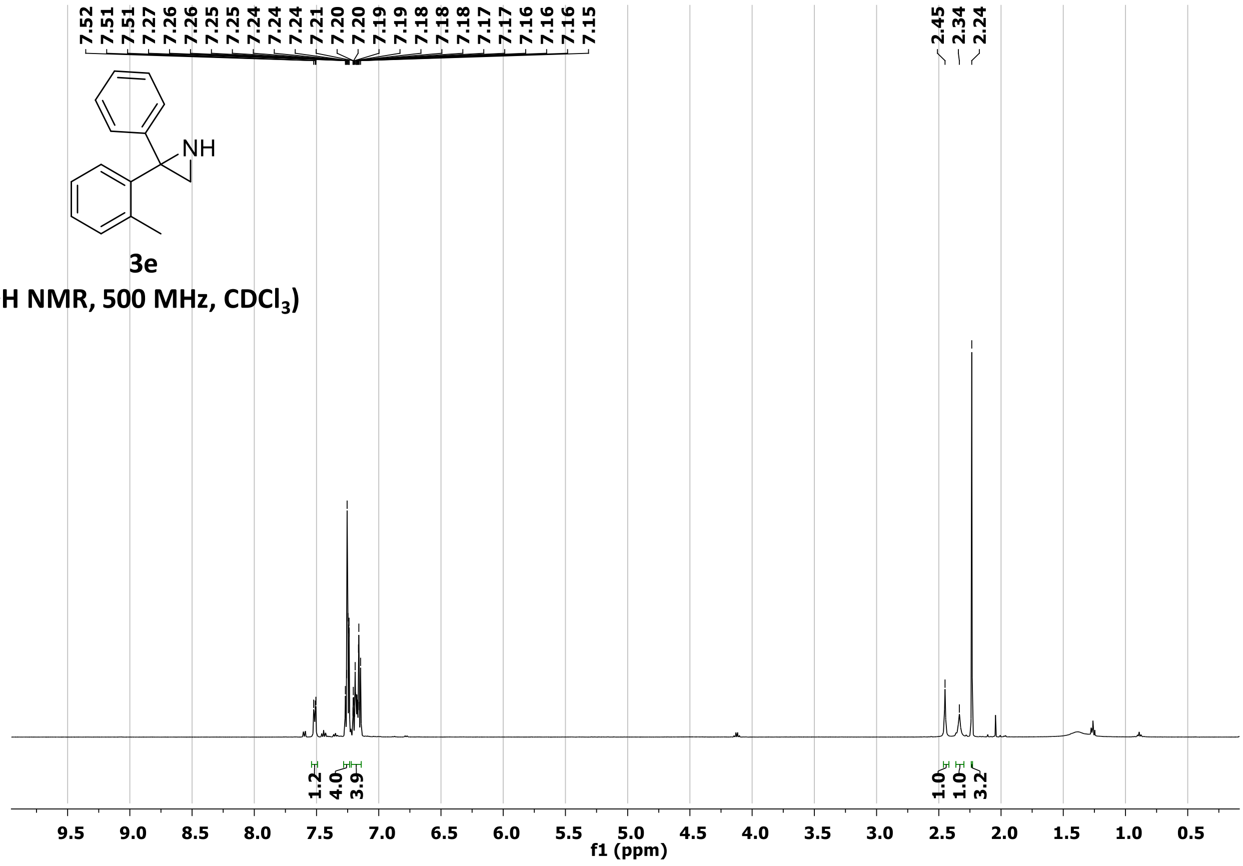


**3e**
**($^1$H NMR, 500 MHz, $CDCl_3$)**

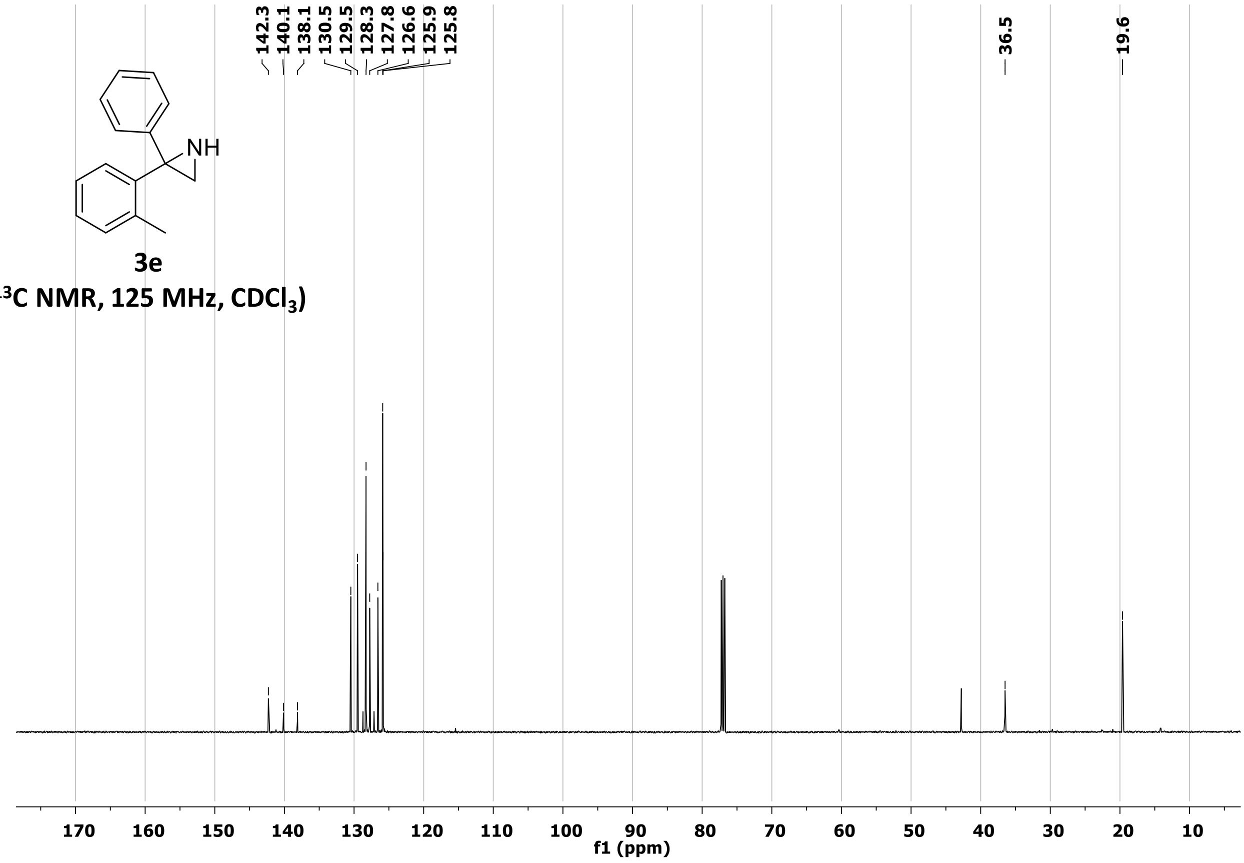


**3e**
**($^{13}$C NMR, 125 MHz, $CDCl_3$)**

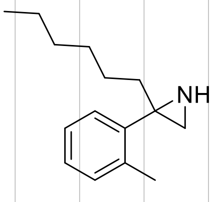

**3f**

**($^{1}$H NMR, 500 MHz, CDCl$_3$)**

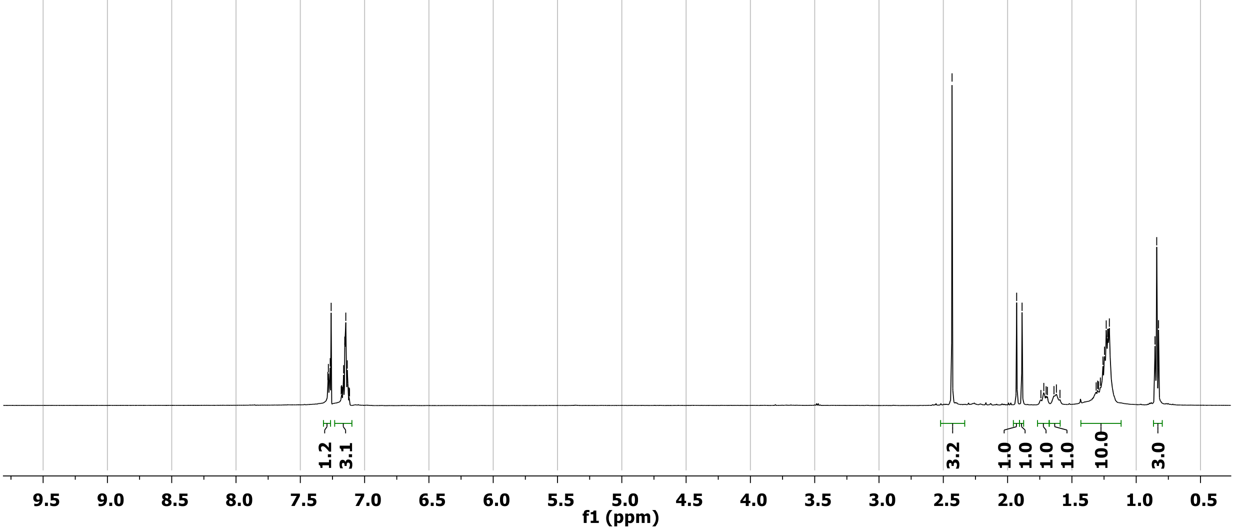


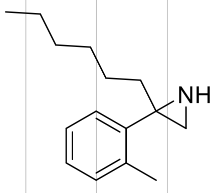

**3f**

**($^{13}$C NMR, 125 MHz, CDCl$_3$)**

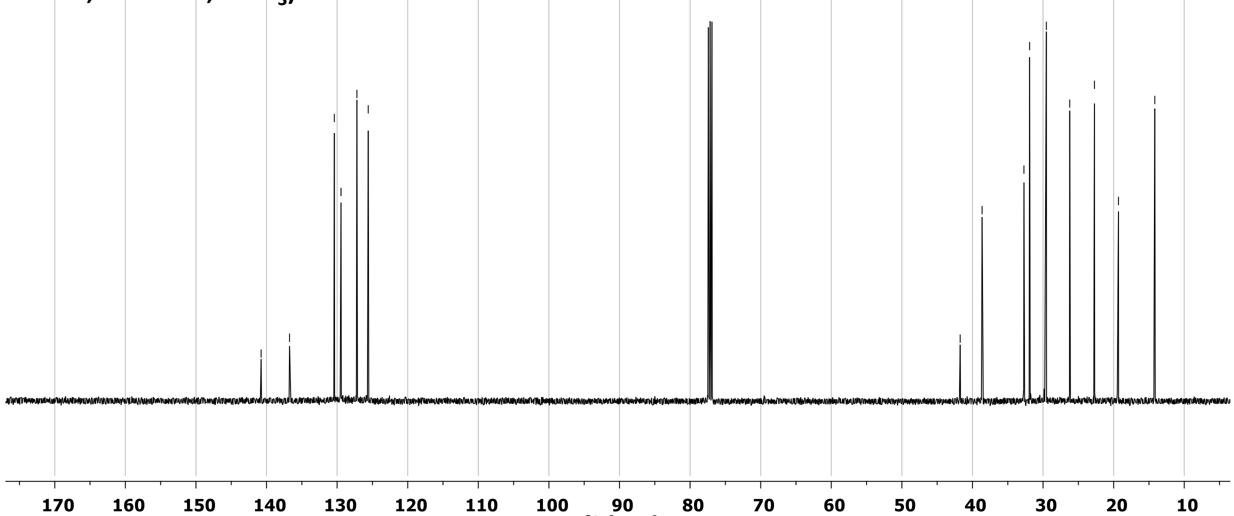

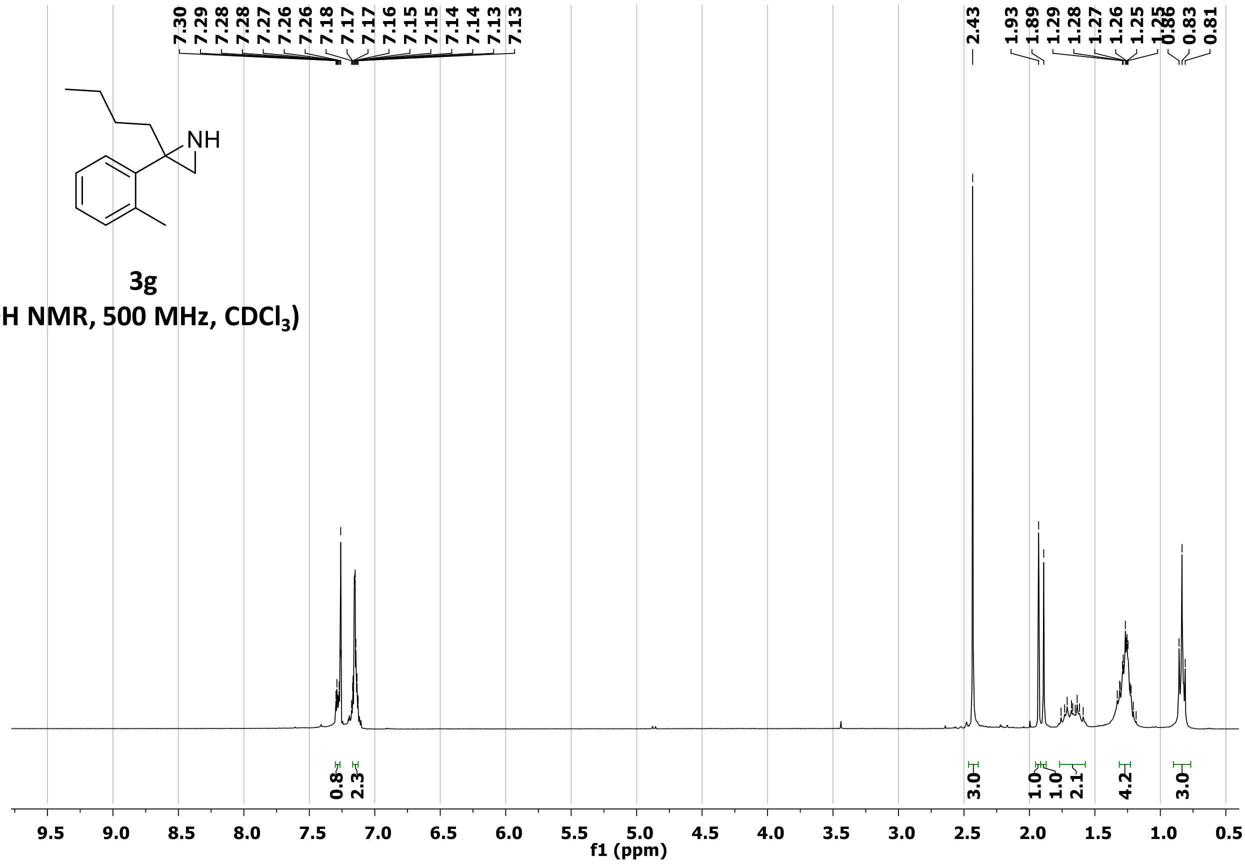


3g
($^1$H NMR, 500 MHz, $CDCl_3$)

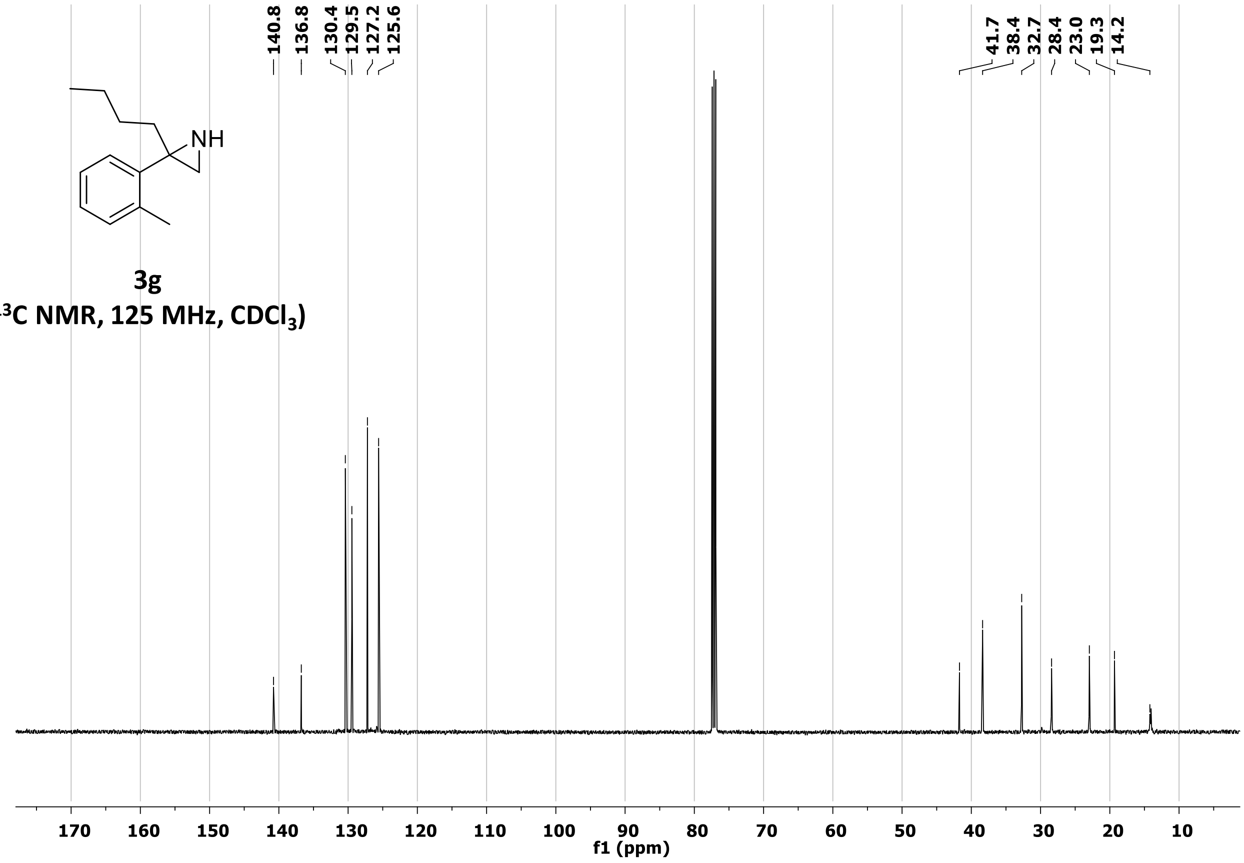


3g
($^{13}$C NMR, 125 MHz, $CDCl_3$)

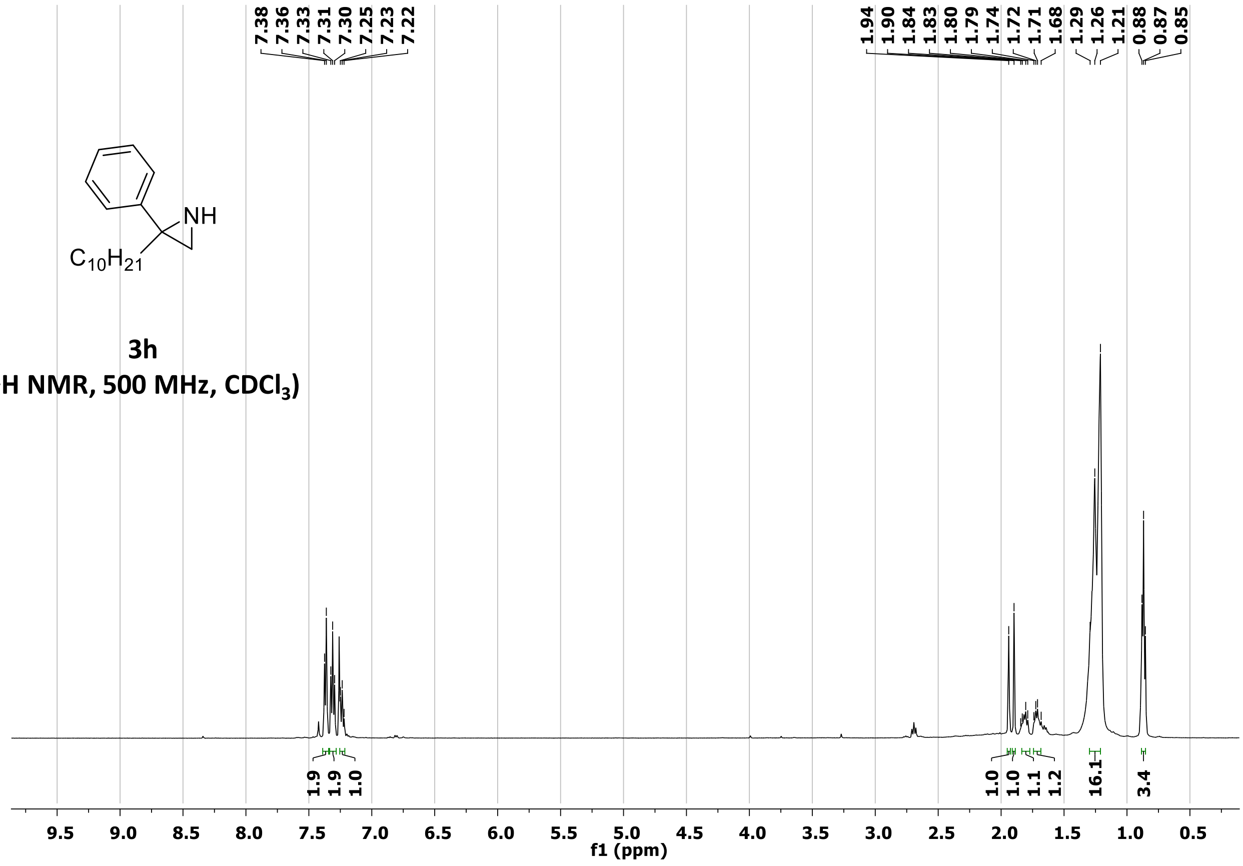


**3h**
**($^{1}$H NMR, 500 MHz, $CDCl_3$)**

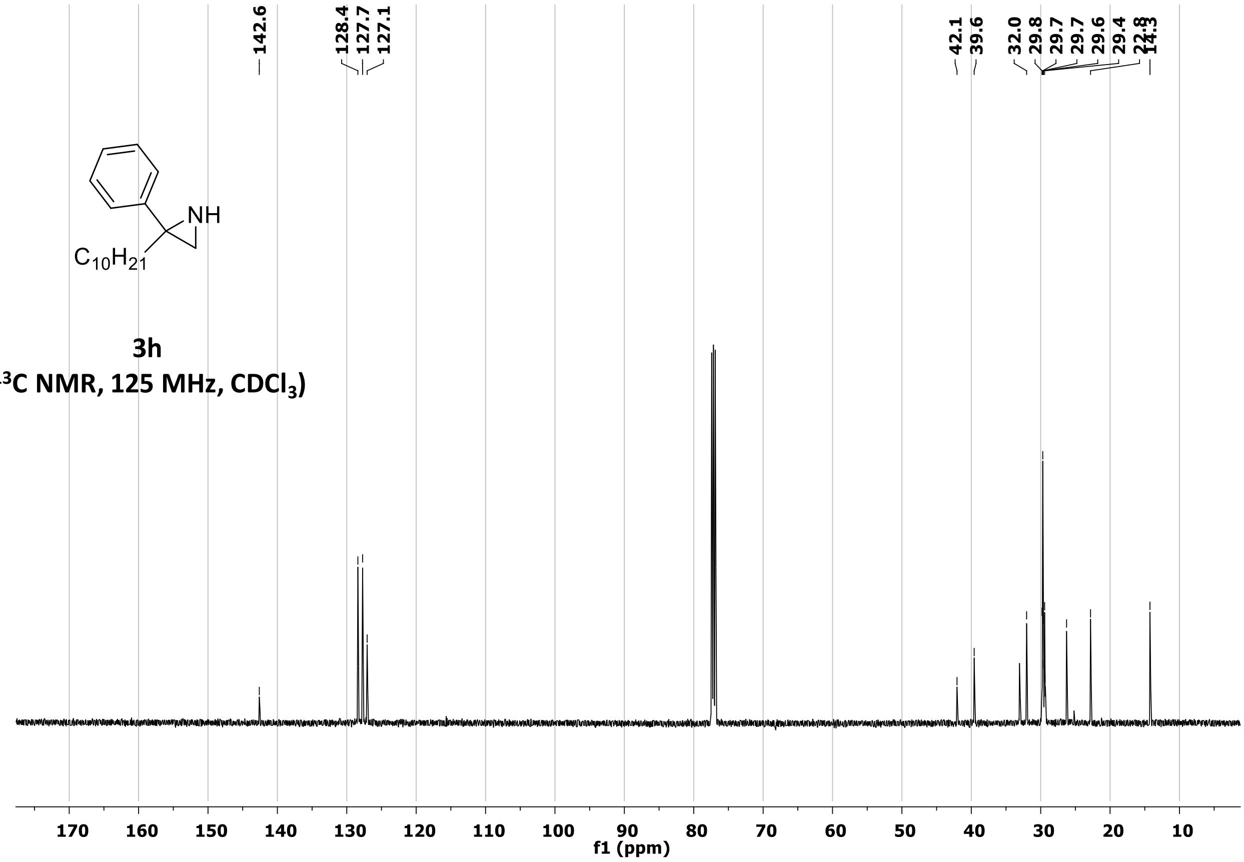


**3h**
**($^{13}$C NMR, 125 MHz, $CDCl_3$)**

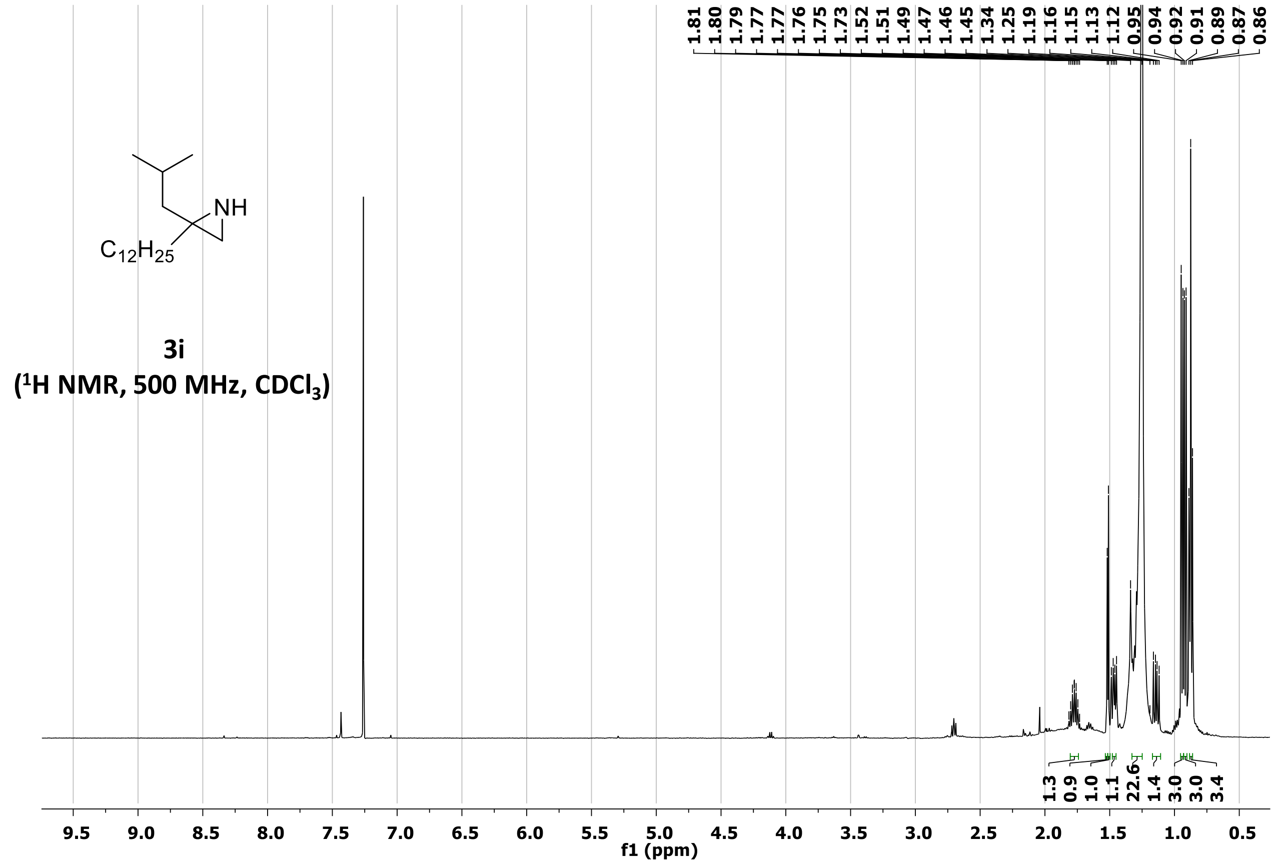


**3i**
**($^1$H NMR, 500 MHz, $CDCl_3$)**

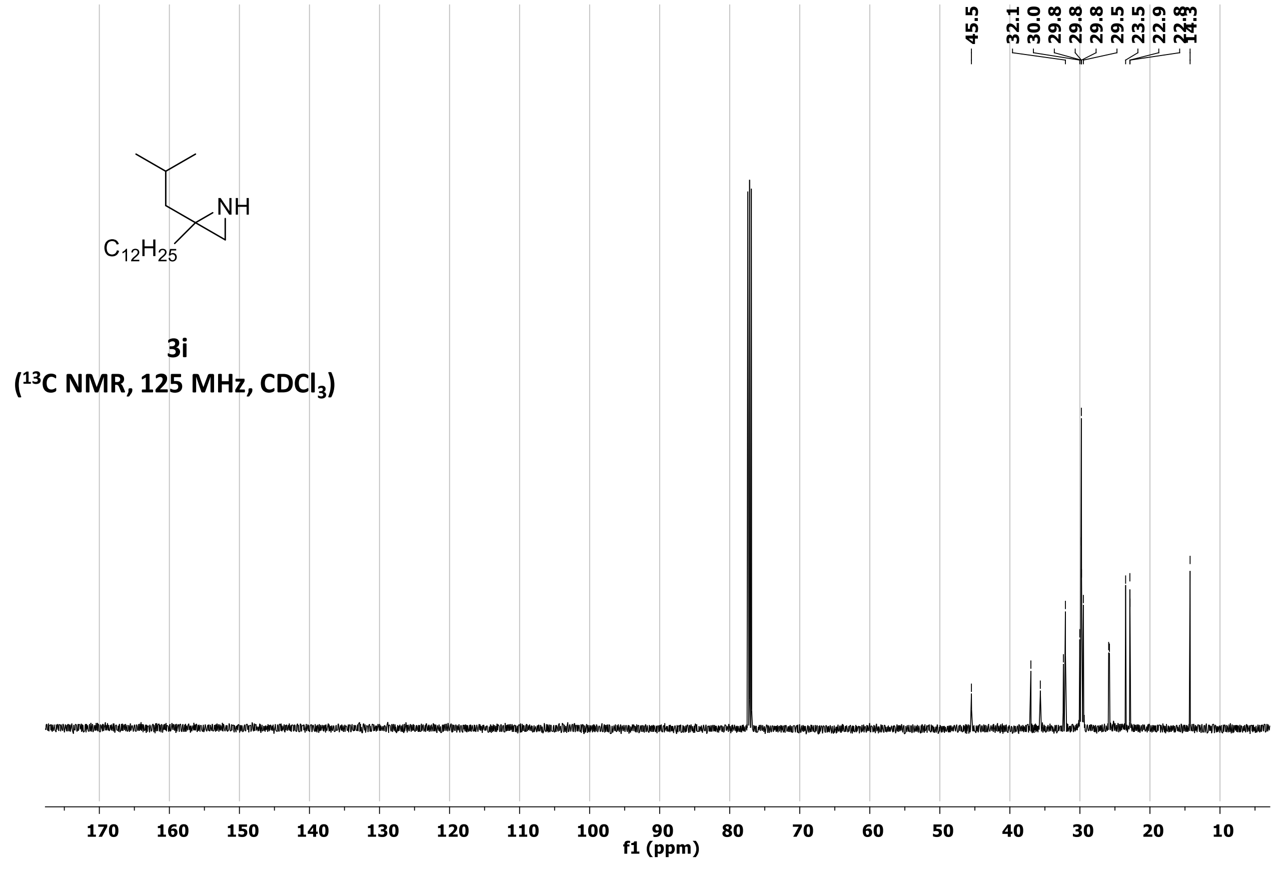


**3i**
**($^{13}$C NMR, 125 MHz, $CDCl_3$)**

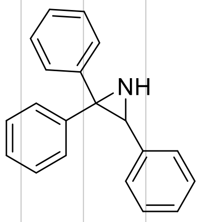

**3j**
**($^{1}$H NMR, 500 MHz, $CDCl_3$)**

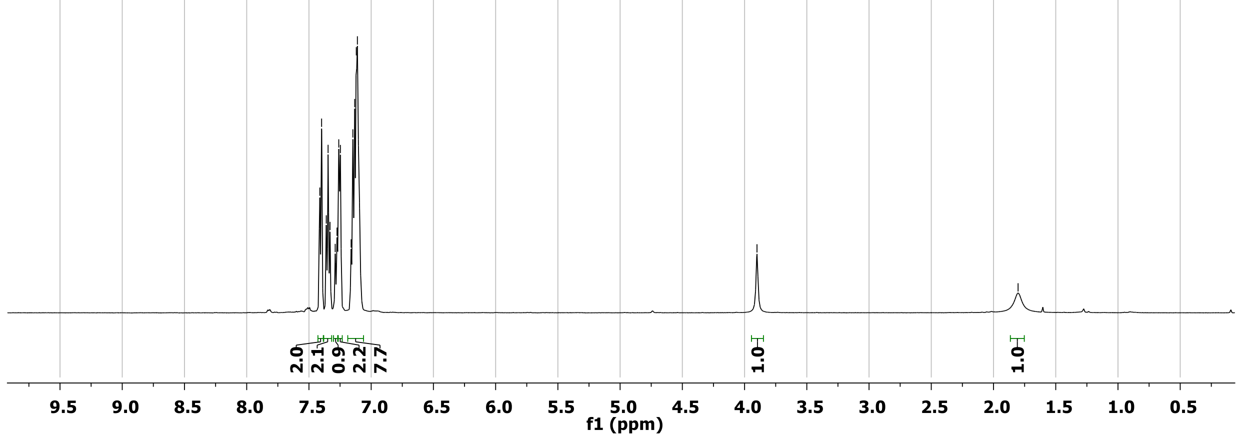


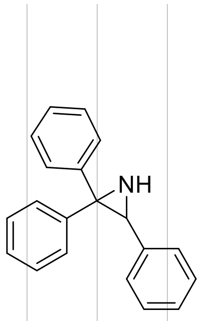

**3j**
**($^{13}$C NMR, 75 MHz, $CDCl_3$)**

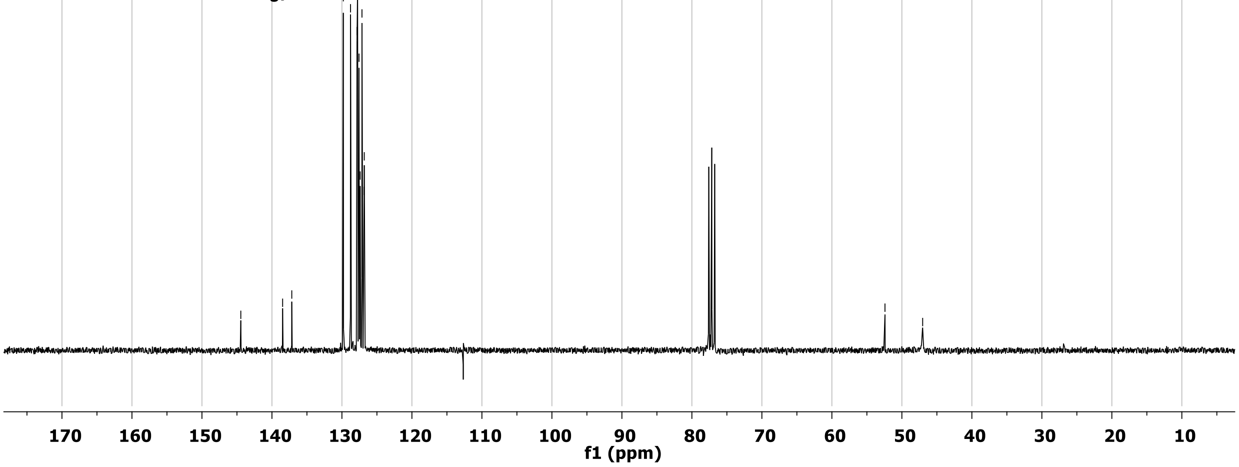

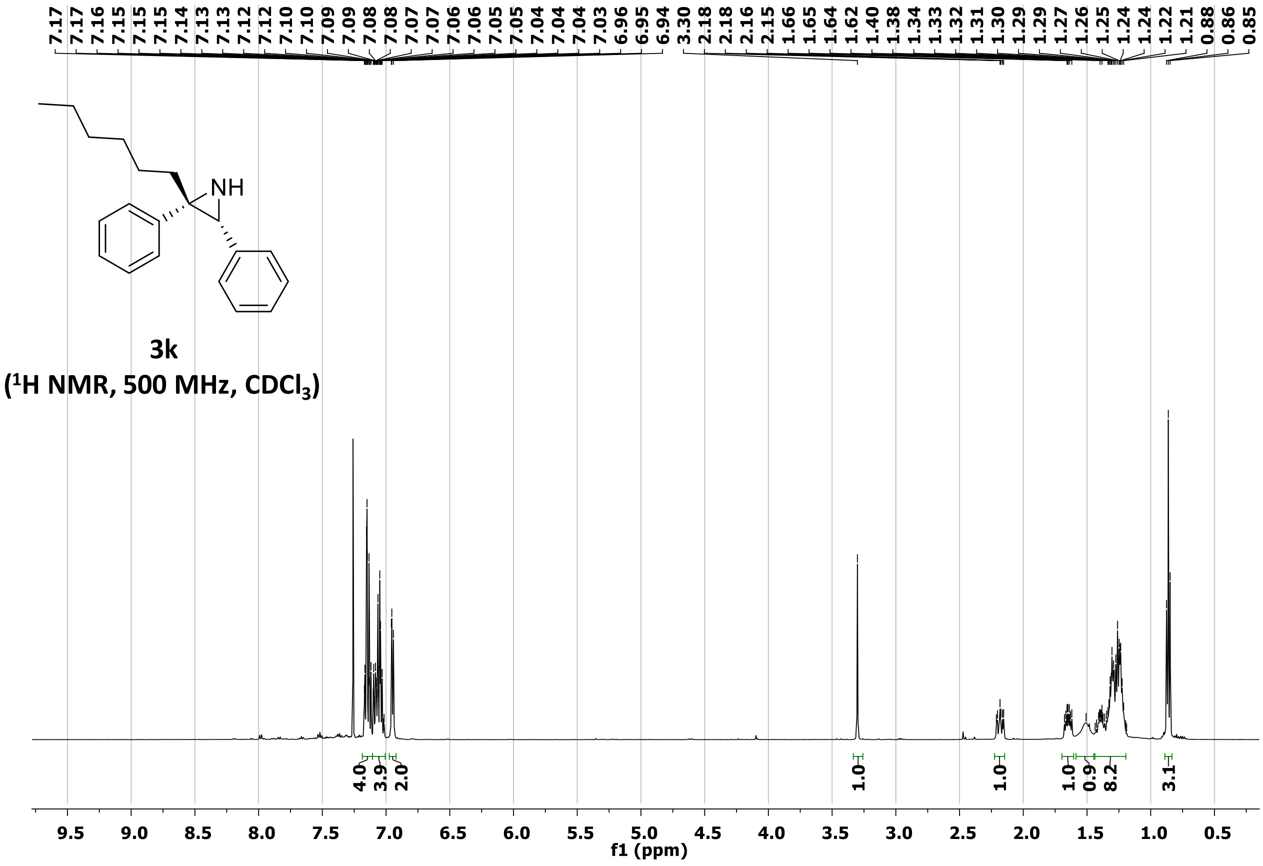


**3k**
**($^1$H NMR, 500 MHz, $CDCl_3$)**

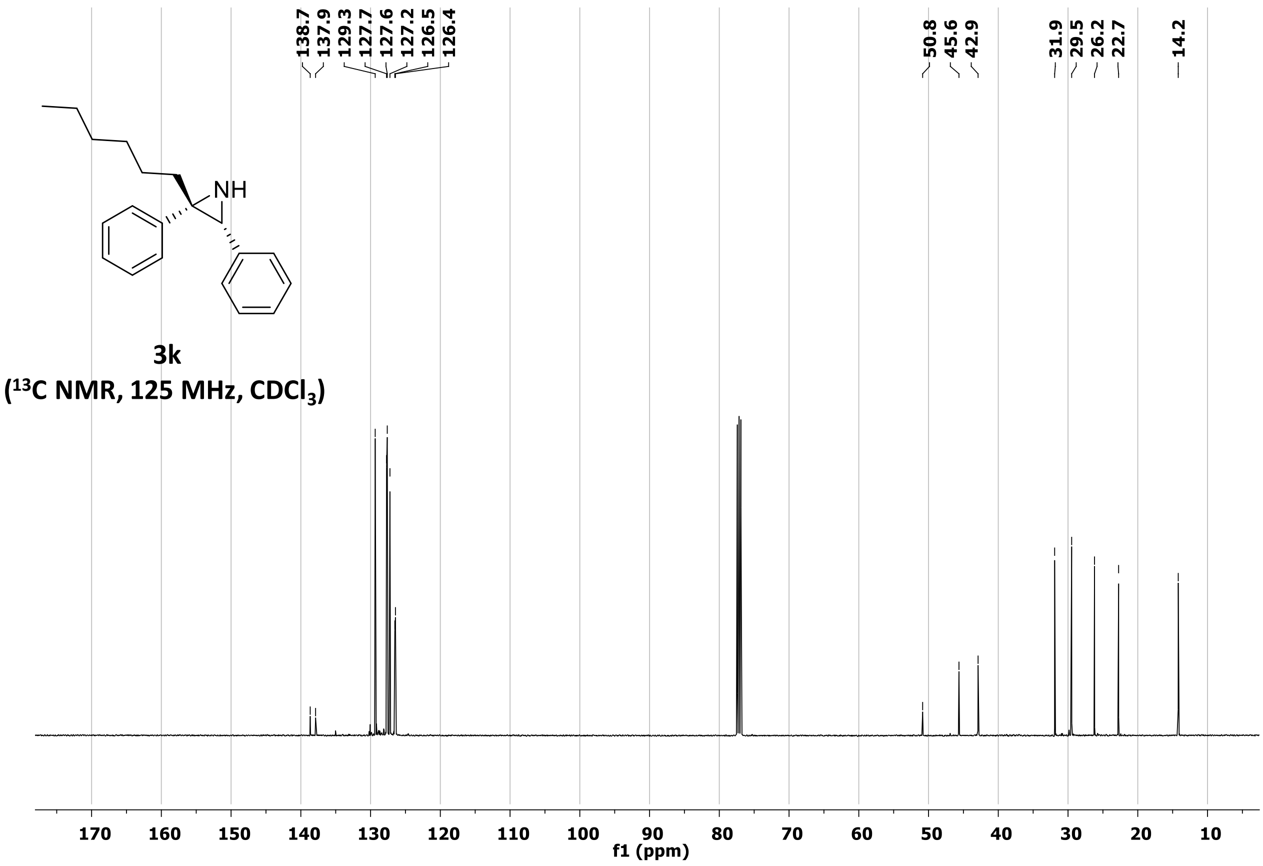


**3k**
**($^{13}$C NMR, 125 MHz, $CDCl_3$)**

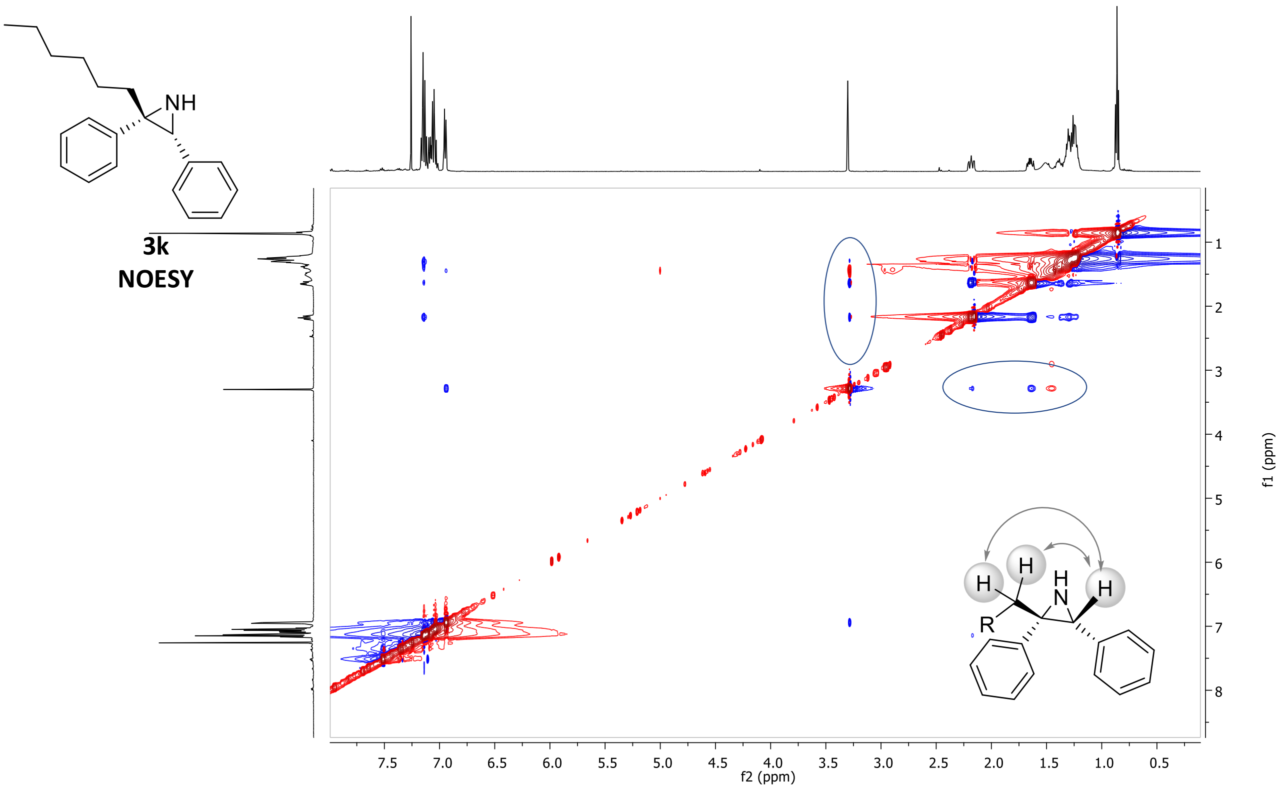

**3k**
**NOESY**

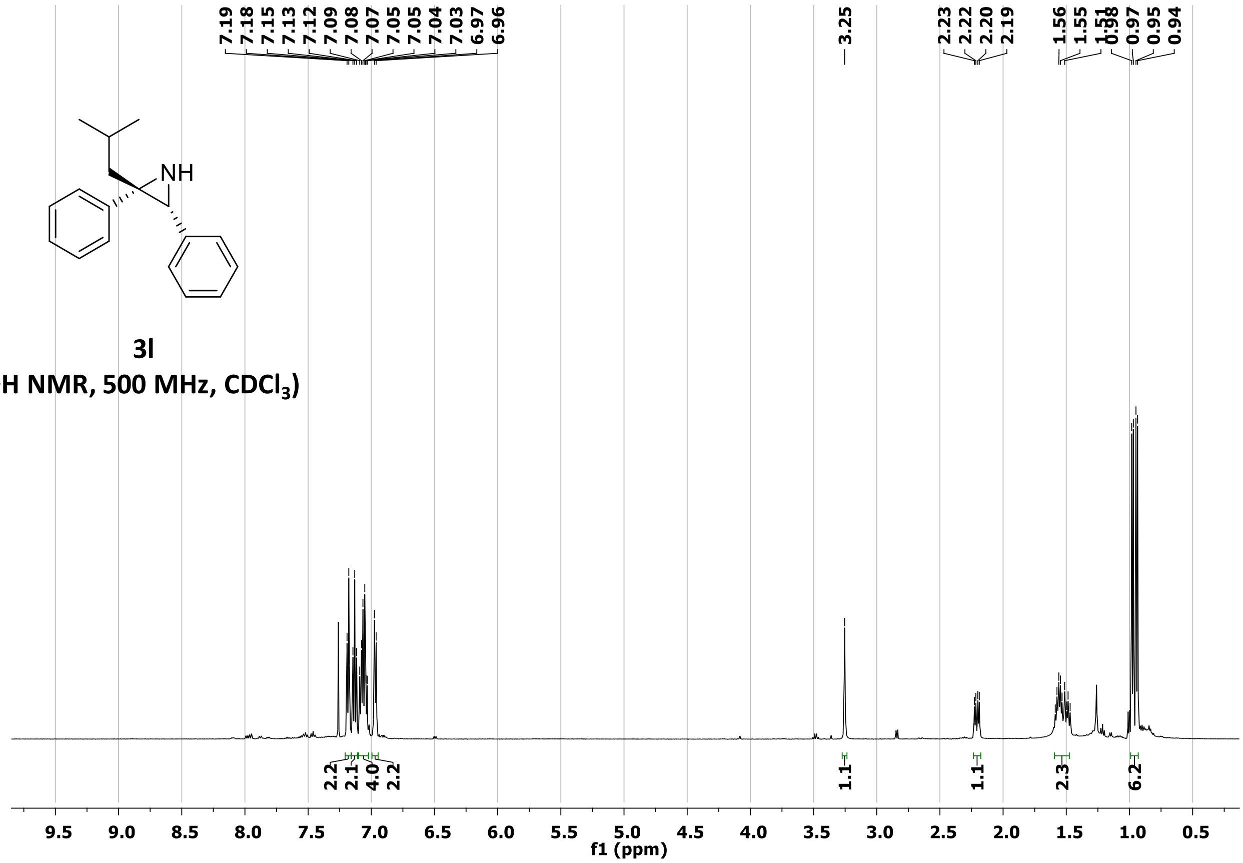

**3l**
**($^1$H NMR, 500 MHz, $CDCl_3$)**

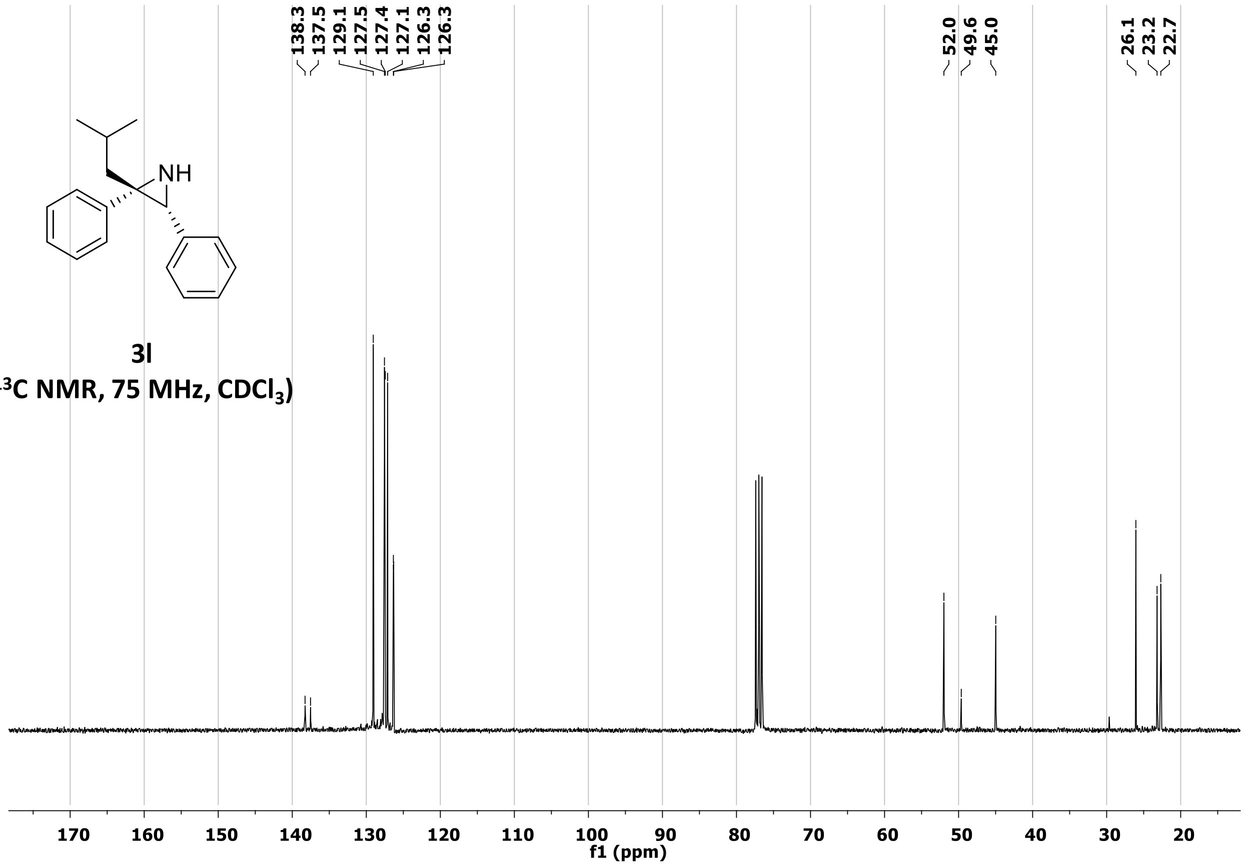


3l
($^{13}$C NMR, 75 MHz, $CDCl_3$)

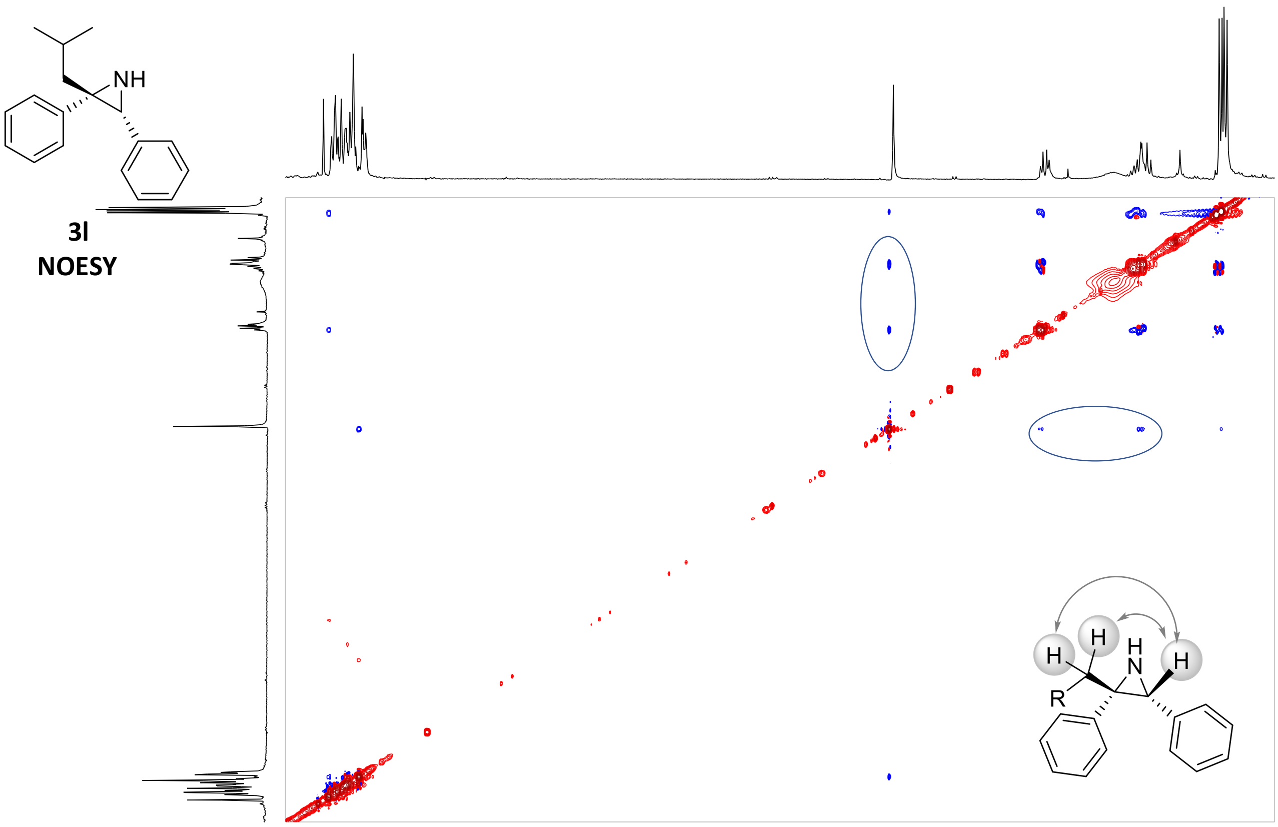


3l
NOESY

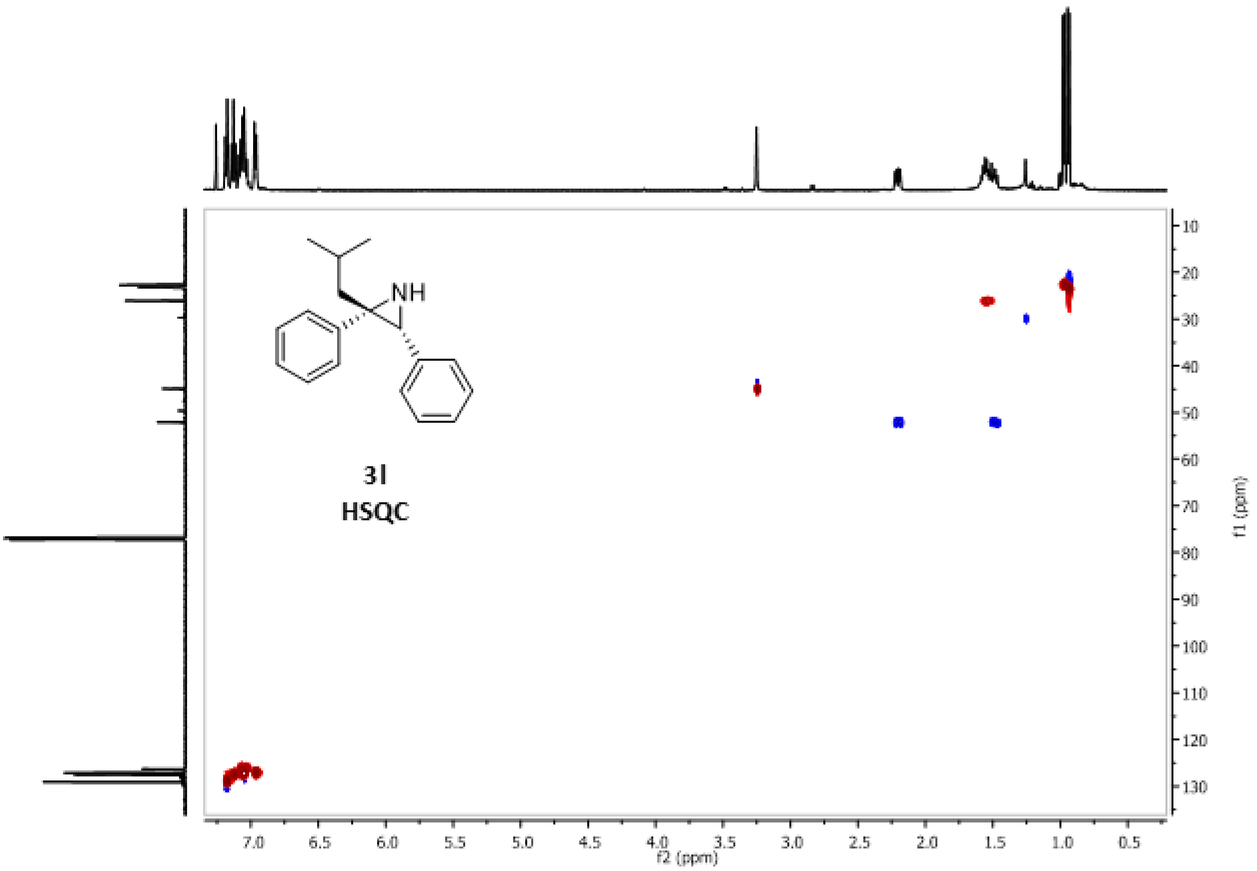


## 6. References


1. Terent'ev, A. O.; Krylov, I. B.; Kokorekin, V. A.; Nikishin, G. I. *Synt. Commun.* **2008**, 38 (21), 3797–3809.

2. Cen, J.; Li, J.; Zhang, Y.; Zhu, Z.; Yang, S.; Jiang, H. *Org. Lett.* **2018**, 20 (15), 4434–4438.

3. Xiang, L.; Niu, Y.; Pang, X.; Yang, X.; Yan, R. *Chem. Commun*. **2015**, 51 (30), 6598–6600.

4. Wu, S.-W.; Liu, F. *Org. Lett.* **2016**, 18 (15), 3642–3645.

5. Li, Z.; Huo, T.; Li, L.; Feng, S.; Wang, Q.; Zhang, Z.; Pang, S.; Zhang, Z.; Wang, P.; Zhang, Z. *Org. Lett.* **2018**, 20 (24), 7762–7766.

6. Li, X.; Liao, S.; Wang, Z.; Zhang, L. *Org. Lett.* **2017**, 19 (14), 3687–3690.

7. Zhou, W.; Zhang, M.; Li, H.; Chen, W. *Org. Lett.* **2016**, 19 (1), 10–13.

8. Khlebnikov, A. F.; Novikov, M. S.; Petrovskii, P. P.; Stoeckli-Evans, H. *J. Org. Chem.* **2011**, 76 (13), 5384–5391.

9. Li, J.; Huang, W.; Chen, J.; He, L.; Cheng, X.; Li, G. *Angew. Chem. Int. Ed.* **2018**, 57 (20), 5695–5698.
